# Self‐Photosensitizing Cobalt Complexes for Photocatalytic CO_2_ Reduction Coupled with CH_3_OH Oxidation

**DOI:** 10.1002/anie.202506060

**Published:** 2025-04-27

**Authors:** Ji‐Hong Zhang, Zhao‐Ming Ge, Di‐Chang Zhong, Jing‐Lin Zuo, Marc Robert, Tong‐Bu Lu

**Affiliations:** ^1^ Institute for New Energy Materials and Low Carbon Technologies School of Materials Science and Engineering Tianjin University of Technology Tianjin 300384 China; ^2^ Key Laboratory of Jiangxi Province for Persistent Pollutants Control and Resources Recycle Nanchang Hangkong University Nanchang 330063 China; ^3^ School of Chemistry & Chemical Engineering State Key Lab Coordination Chemistry Nanjing University Nanjing 210023 China; ^4^ Institut Parisien de Chimie Moléculaire Sorbonne Université, CNRS, IPCM Paris F‐75005 France; ^5^ Institut Universitaire de France (IUF) Paris F‐75005 France

**Keywords:** CO_2_ reduction, Homogeneous catalyst, Methanol oxidation, Non‐noble metal complex, Self‐photosensitizing

## Abstract

The use of metal complexes as homogeneous molecular catalysts has attracted considerable attention regarding photocatalytic CO_2_ reduction. Enhancing these complexes with photosensitivity and photooxidation capabilities, aiming to create multifunctional molecular devices, presents significant challenges. In response to these challenges, we successfully designed and synthesized three innovative metal complexes. The complexes demonstrate a remarkable ability to perform CO_2_ photoreduction in tandem with methanol photooxidation, allowing for the simultaneous production of formic acid without requiring additional photosensitizers and electron sacrificial reductants. An optimal turnover number (TON) value of 855 was obtained under simulated sunlight. Even under natural sunlight, the TON can reach 207, much higher than the value of the physical mixture of the photocatalytic reductive and oxidative moieties. Spectroscopic studies and density functional theory (DFT) calculations revealed that integrating reduction and oxidation sites in one molecular catalyst can promote charge transfer kinetics and enhance activity for CO_2_ reduction and methanol oxidation. This is the first report that non‐noble metal homogeneous catalysts can simultaneously possess photosensitivity, photoreduction, and photo‐oxidation functions, offering new insights into designing homogeneous catalysts for artificial photosynthesis.

## Graphical abstract

Self‐photosensitizing non‐noble metal complexes achieve CO_2_ photoreduction coupled with CH_3_OH photooxidation, simultaneously synthesizing formic acid in the absence of any additional photosensitizers and electron sacrificial reductants.

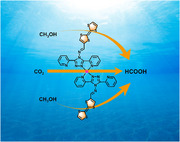

## Introduction

**Scheme 1:**
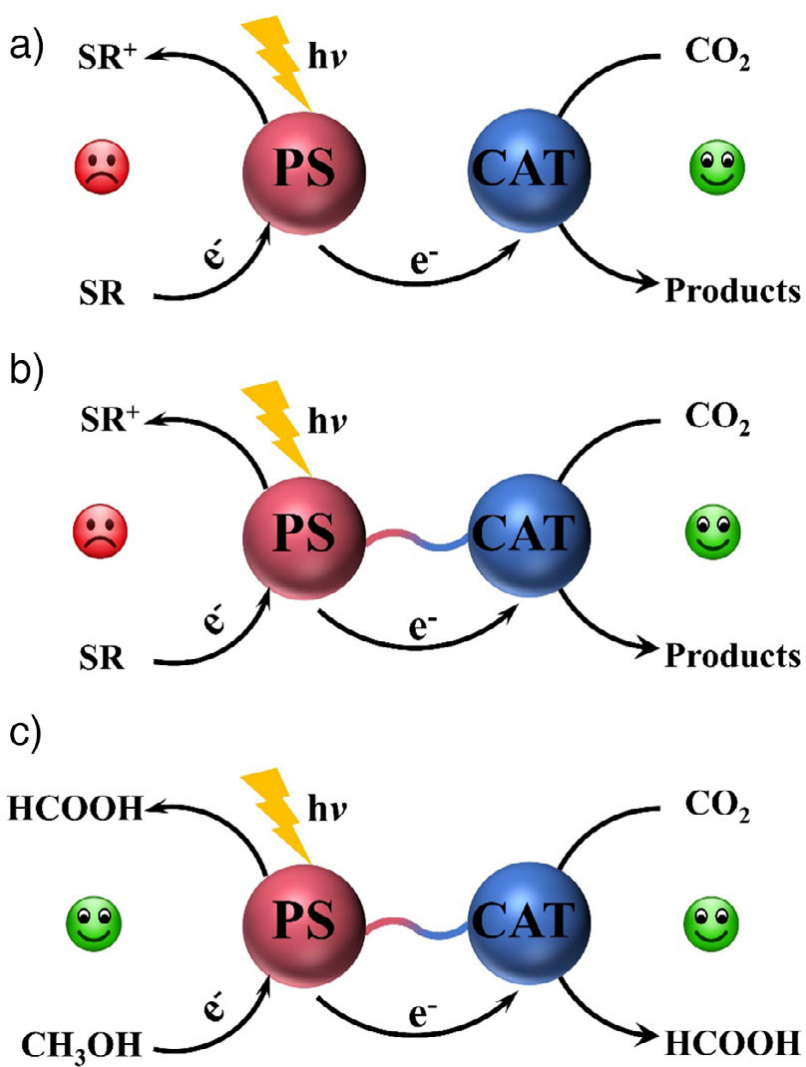
Schematic representations of homogeneous photocatalytic CO_2_‐reduction systems: a) Conventional system; b) covalently bonded PS (photosensitizer) and CAT (catalyst) system; c) covalently bonded PS and CAT system without added external SR (sacrificial reductant) as reported in this work.

The global focus on achieving carbon neutrality has intensified in response to the energy crisis and environmental problems stemming from the significant surge in CO_2_ emissions because of the excessive combustion of fossil fuels.^[^ ^1^, ^2^, ^3^, ^4^ ^]^ Reduction of CO_2_ to value‐added chemicals by photocatalysis is recognized as one of the most promising technologies for sustainable carbon recycling.^[^ ^5^, ^6^, ^7^, ^8^, ^9^, ^10^, ^11^, ^12^ ^]^ Conventional homogeneous photocatalytic systems for CO_2_ reduction usually involve a photosensitizer (PS), a catalyst (CAT), and a sacrificial reductant (SR; Scheme 1a).^[^ ^13^, ^14^, ^15^, ^16^, ^17^, ^18^, ^19^, ^20^, ^21^, ^22^, ^23^, ^24^, ^25^, ^26^, ^27^, ^28^ ^]^ Driven by light, the photosensitizer plays an important role in transferring reducing equivalents from the sacrificial reductant to the catalytic centre in the catalyst. The latter coordinates CO_2_ and reduces it with the help of protons as cocatalysts. In these systems, electron transfers occur upon diffusional collisions between these components, leading to low efficiency and limitations by the diffusional process itself and not by the intrinsic activity of the catalyst.

To accelerate intermolecular electron transfer from photosensitizer to catalyst, covalently linking the photosensitive and catalytic moiety has been conceived and evidenced as an effective approach (Scheme 1b).^[^ ^29^, ^30^, ^31^, ^32^, ^33^, ^34^, ^35^, ^36^, ^37^, ^38^, ^39^, ^40^ ^]^ For instance, Nakajima group developed a binuclear Ru(II)‐Re(I) complex by connecting a Ru photosensitive unit with a Re catalytic unit, which can serve as a photocatalyst to reduce CO_2_ to CO, with a turnover number (TON) value of over 1000.^[^ ^32^ ^]^ Kamada et al. reported an efficient tetradentate PNNP‐type Ir photocatalyst, which achieved a TON of 2560 for CO_2_ reduction to HCOOH and CO in the presence of 1,3‐dimethyl‐2‐phenyl‐2,3‐dihydro‐1*H*‐benzo[*d*]imidazole (BIH) as sacrificial reductant.^[^ ^41^ ^]^ Very recently, Yuan and co‐workers synthesized a multimetallic Fe_2_Na_3_ purpurin complex, which can serve as a self‐photosensitizing catalyst for CO_2_ reduction, achieving a TON value of 2625 and a selectivity of 91% to CO in 120 h, with BIH as a sacrificial reductant.^[^ ^42^ ^]^ Remarkably, significant progress has been made in accelerating electron transfer between photosensitizer and catalyst in homogeneous photocatalytic CO_2_ reduction. Nevertheless, noble‐metal catalysts/photosensitizers and/or sacrificial reductants are necessary for these photocatalytic systems. In particular, costly sacrificial reductants, often amines such as BIH, triethanolamine (TEOA), triethylamine (TEA), and 1‐benzyl‐1,4‐dihydronicotinamide (BNAH) should be avoided. Alternative organic compounds that can be oxidized to high value‐added products may be used instead and should get more attention (Scheme 1c). To the best of our knowledge, homogeneous photocatalytic systems for CO_2_ reduction based on non‐noble‐metal catalysts/photosensitizers in the absence of sacrificial reductants have not been reported to date. Thus, it would be meaningful to develop homogeneous multifunctional photocatalysts integrating photosensitive unit, catalytic reductive and catalytic oxidative moieties, so as to achieve the coupling of CO_2_ reduction with a useful organic oxidation reaction.

**Figure 1:**
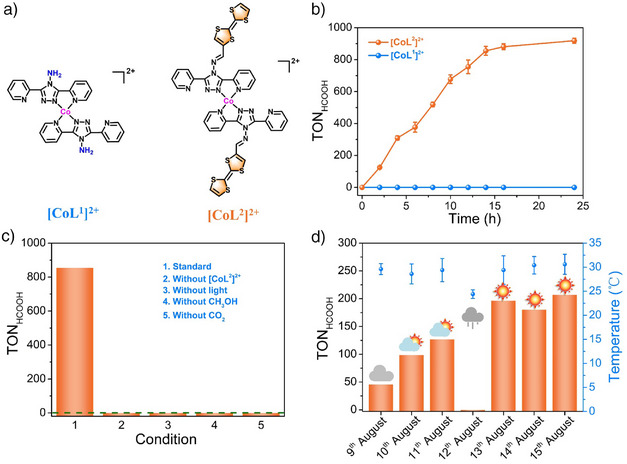
a) Chemical structures of homogeneous catalysts of [CoL^1^]^2+^ and [CoL^2^]^2+^. b) Time‐dependent HCOOH generation from the photocatalytic CO_2_ reduction coupled with CH_3_OH oxidation using [CoL^1^]^2+^ or [CoL^2^]^2+^ (The error bars are standard deviations calculated from the results of three parallel experiments). c) Control experiments for the photocatalytic CO_2_ reduction coupled with CH_3_OH oxidation using [CoL^2^]^2+^. d) Photocatalytic performance of [CoL^2^]^2+^ for the photocatalytic CO_2_ reduction coupled with CH_3_OH oxidation driven by sunlight, the error bars represent the temperature range of photocatalytic set‐up during catalysis under the sunlight irradiation from 9:00 am to 4:00 pm (Date: 9–15/8/2023; Position: TJUT, Xiqing district, Tianjin). Catalytic conditions: Catalysts (1 µM), 5 mL CO_2_‐saturated CH_3_OH/H_2_O (*v*:*v* = 4:1) solution, and 300 W Xe lamp (320 < *λ* < 780 nm; light intensity: 200 mW cm^−2^), irradiation time 14 h, and temperature *T* = 25 °C.

Electron‐rich tetrathiafulvalene (TTF) is a powerful electron donor with good photon‐absorption properties.^[^ ^43^, ^44^, ^45^ ^]^ After donating electrons, the resulting cation radical TTF^•+^ possesses a strong oxidation capacity. A covalently bonded TTF with a non‐noble metal complex may thus act as a multifunctional catalyst achieving the coupling of CO_2_ photoreduction with an organic oxidation reaction. Herein, on the basis of chemical modification of a mononuclear [CoL^1^]^2+^ complex (L^1^ = 4‐amino‐3,5‐bis(pyridine‐2‐yl)‐1,2,4‐triazole) with TTF groups, we obtained an example of non‐noble metal complex of [CoL^2^]^2+^ (L^2^ = (*E*)‐1‐([2,2′‐bi(1,3‐dithiolylidene)]‐4‐yl)‐*N*‐(3,5‐di(pyridine‐2‐yl)‐4*H*‐1,2,4‐triazol‐4‐yl) methenamine), which contain a mononuclear Co(II) catalytic reduction moiety, and two TTF moieties playing the role of photosensitizer (Figure 1a). Photocatalytic experiments show that [CoL^2^]^2+^ complex possesses activity for CO_2_ reduction and CH_3_OH oxidation, simultaneously synthesizing formic acid (HCOOH), and achieving a high turnover number (TON) value of 855 for HCOOH in the absence of additional photosensitizers or sacrificial reductants under laboratory light source. It surpasses the performance of most reported non‐noble metal self‐photosensitizing molecular catalysts and even outperforms noble‐metal self‐photosensitizing molecular catalysts (Table S1). Moreover, it was found that natural sunlight could also drive this catalytic system to produce HCOOH, with a TON value up to 207. Spectroscopic experiments and density functional theory (DFT) calculations revealed that integrating oxidation and reduction moieties in one complex promotes charge transfer kinetics and enhances the activity for CO_2_ reduction and CH_3_OH oxidation. Using the same protocol, the resulting [NiL^2^]^2+^ and [CuL^2^]^+^ also show activity for CO_2_ photoreduction and CH_3_OH photo‐oxidation, illustrating the generality of this approach.

## Results and Discussion

The ligands L^1^ and L^2^ were synthesized by modified literature methods (see details in Supporting Information, Figures S1–S6).^[^ ^46^, ^47^, ^48^ ^]^ Subsequently, [CoL^1^]^2+^ and [CoL^2^]^2+^ were obtained by the reaction of Co(H_33_C_16_SO_3_)_2_(H_2_O)_2_ and Co(NO_3_)_2_·6H_2_O with corresponding ligands at room temperature, respectively. Their structures were characterized by IR spectroscopy, elemental analysis, UV–vis absorption spectroscopies, liquid chromatography‐mass spectrometry (LC‐MS), X‐ray photoelectron spectroscopic (XPS), and X‐ray absorption spectroscopy (XAS) (Figures S7–S18). UV–vis absorption spectra reveal a sharp peak at 288 nm (*ε*_max_ = 42 024 M^−1^ cm^−1^) for [CoL^1^]^2+^ (Figure S9). The UV–vis spectrum of TTF displays three absorption peaks at 305, 362, and 448 nm (*ε* = 9712, 1631, and 261 M^−1^ cm^−1^, respectively, Figure S4). In the case of [CoL^2^]^2+^, a maximum peak at 297 nm (*ε*_max_ = 51 819 M^−1^ cm^−1^) and a broad band spanning from 378 to 634 nm are observed (Figure S10), suggesting a redshift in the absorption peak of [CoL^2^]^2+^ following the incorporation of TTF. The results of LC‐MS measurements for [CoL^1^]^2+^ and [CoL^2^]^2+^ in CH_3_OH show ion peaks at *m/z* = 840.36 and 1024.92, corresponding to the species of [[CoL^1^]^2+^+H_33_C_16_SO_3_^−^]^+^ and [[CoL^2^]^2+^+NO_3_^−^]^+^, respectively (Figures S11–S14). The XPS measurements showed that the binding energy peaks for Co 2p_1/2_ and Co 2p_3/2_ appear at 796.62 and 780.78 eV, respectively, indicating the chemical valence of cobalt in[CoL^2^]^2+^ is +2 (Figure S15).^[^ ^34^ ^]^ Similarly, the oxidation state of cobalt in [CoL^1^]^2+^ was also determined to be +2 (Figure S16). Furthermore, X‐ray absorption near‐edge structure (XANES) analysis reveals that the absorption edge of [CoL^2^]^2+^ closely resembles that of CoO (Figure S17), further corroborating the +2 valence state of cobalt in [CoL^2^]^2+^, consistent with the XPS and LC‐MS findings. Moreover, fitting results indicate that the simulated Co Fourier transform‐extended X‐ray absorption fine structure (FT‐EXAFS) spectra based on our architectural model are in good agreement with the experimental results (Figure S18). Taken together, all these observations suggest the successful syntheses of [CoL^1^]^2+^ and [CoL^2^]^2+^, which are stable in CH_3_OH solution.

The experiments of photocatalytic CO_2_ reduction with CH_3_OH oxidation were performed with a 300 W Xe lamp (320 < *λ* < 780 nm), using [CoL^1^]^2+^ or [CoL^2^]^2+^ as a photocatalyst under CO_2_ atmosphere in 5 mL CH_3_OH/H_2_O (*v*:*v* = 4:1) solution, without additional photosensitizers and sacrificial reductants (see the Supporting Information). The possibly generated gaseous and liquid products were detected and quantified by gas chromatography (Figure S19) and ion chromatography (Figure S20), respectively. The results show that for [CoL^1^]^2+^, negligible gaseous and liquid products were detected during the photocatalytic process, while for [CoL^2^]^2+^, a large amount of HCOOH liquid product was observed. Specifically, as shown in Figures 1b, S21, after illumination for 14 h, no HCOOH was detected in the photocatalytic reaction over 1 µM of [CoL^1^]^2+^. Under similar conditions, a significant amount of HCOOH can be generated and detected over 1 µM of [CoL^2^]^2+^, corresponding to a TON value of 855 (two‐thirds of the formic acid was produced comes from CO_2_ reduction and one‐third from CH_3_OH oxidation) (Figures 1b, S21). The apparent quantum yield (AQY) for HCOOH production was determined to be 0.24%, based on the photocatalytic result using an LED light during a 10 h (*λ* = 365 nm) experiment (Figure S22). Besides HCOOH, trace amount of HCHO was also detected (Figures S23, S24), which is the intermediate product during the process of CH_3_OH oxidation to HCOOH.^[^ ^49^ ^]^ Despite the quantum yield for HCOOH production is higher than most reported self‐photosensitive homogeneous catalysts,^[^ ^29^, ^30^, ^31^, ^32^, ^33^, ^34^, ^35^, ^36^, ^37^, ^38^, ^39^, ^40^ ^]^ the value also indicates that large number of photons have not been utilized. Dynamic light scattering (DLS) measurements showed that there were no particles in the catalytic system using [CoL^2^]^2+^ after 14 h irradiation (Figure S25). The result of photocatalytic CO_2_ reduction coupled with CH_3_OH oxidation in the presence of mercury shows that the amount of HCOOH generated is almost equal to that without mercury, suggesting that the catalytic reaction is homogeneous (Figure S26).^[^ ^15^ ^]^ However, the rate of HCOOH generation after 14 h decreased. The results of UV–vis spectrum of the reaction solution after catalysis for 14 h indicate that the decline in catalytic activity is primarily due to the photodegradation of [CoL^2^]^2+^ (Figures S27, S28).

It is interesting to observe that [CoL^1^]^2+^ and [CoL^2^]^2+^ show completely different photocatalytic activity in coupling CO_2_ reduction with CH_3_OH oxidation. Considering that the only structural difference between [CoL^1^]^2+^ and [CoL^2^]^2+^ is that there are two TTF groups covalently bonded in [CoL^2^]^2+^, we speculate that TTF plays a significant role in this comproportionation reaction. To confirm this speculation, further photocatalytic reaction over the physical mixture of [CoL^1^]^2+^ and TTF ([CoL^1^]^2+^&TTF) was performed. It was found that 0.90 µmol of HCOOH was detected (Figure S21). Although the amount of HCOOH generated by [CoL^1^]^2+^&TTF with molar ratio of 1:2 is much lower than that of [CoL^2^]^2+^ in which TTF is covalently bonded to [CoL^1^]^2+^, this result still demonstrates the important role of TTF in this coupling reduction and oxidation reaction. Further increase of the TTF amount, the HCOOH yield of [CoL^1^]^2+^ and TTF catalytic systems increased (Figure S28). When the molar ratio of [CoL^1^]^2+^ to TTF was 1:50, a maximum amount of HCOOH was generated. However, upon further increasing the TTF amount, a decline in the yield of HCOOH was observed, which may be due to the excessive TTF hindering the light absorption and utilization.^[^ ^50^ ^]^ Besides, trace amounts of HCOOH were detected with L^2^ as catalyst (Figure S21), which also confirms the role of TTF in the photocatalytic process. The covalent bonding of TTF with [CoL^1^]^2+^ through a Schiff base reaction results in the creation of [CoL^2^]^2+^, a compound with enhanced capabilities. This new complex exhibits photosensitive properties and can perform both photoreduction and photo‐oxidation functions. By integrating these multiple functions and satisfying the requirements for photocatalytic reactions, [CoL^2^]^2+^ demonstrates exceptional photocatalytic performance in coupling CO_2_ reduction with CH_3_OH oxidation, simultaneously synthesizing HCOOH. The different activity of [CoL^2^]^2+^ and [CoL^1^]^2+^&TTF also highlights that the covalent bonding is beneficial for accelerating the electron transfer between TTF and the Co(II) catalytic center, which has been observed in other heterogeneously photocatalytic systems with molecular catalysts.^[^ ^44^, ^51^, ^52^, ^53^, ^54^ ^]^

To further explore this strategy based on ligand L^2^, we synthesized the Ni(II) complex ([NiL^2^]^2+^) and the Cu(I) complex ([CuL^2^]^+^) by a similar synthetic procedure (Figure S29). The IR spectroscopy, elemental analysis, UV–vis absorption spectroscopies, LC‐MS, XPS, and XAS demonstrated the successful syntheses of [NiL^2^]^2+^ and [CuL^2^]^+^ (Figures S30–S41). The UV–vis spectrum of [NiL^2^]^2+^ and [CuL^2^]^+^ also displays a maximum peak at 296.5 nm (ε_max_ = 51 158 and 58 056 M^−1^ cm^−1^, respectively) and a broad band spanning 378 to 634 nm (Figures S32, S33), which is similar to [CoL^2^]^2+^. The Ni 2p XPS spectra in [NiL^2^]^2+^ show two main characteristic peaks at 873.32 and 855.75 eV, which are assigned to the Ni 2p_1/2_ and Ni 2p_3/2_, respectively, indicating the chemical valence of nickel in [NiL^2^]^2+^ is +2 (Figure S38).^[^ ^34^ ^]^ The Auger electron spectroscopy (AES) of the Cu LMM only showed a peak at 570.30 eV assignable to Cu(I) species (Figure S39), suggesting the oxidation valence of copper in [CuL^2^]^+^ is +1.^[^ ^55^ ^]^ Photocatalytic experiments showed that as expected, both [NiL^2^]^2+^ and [CuL^2^]^+^ are photosensitive, and can achieve CO_2_ photoreduction coupled with CH_3_OH photooxidation, to simultaneously synthesize HCOOH, corresponding to TON values of 405 and 62, respectively (Figure S42). A trace amount of intermediate product HCHO was also detected (Figure S43). The apparent quantum yields of [NiL^2^]^2+^ and [CuL^2^]^+^ to HCOOH were determined to be 0.13% and 0.0051%, respectively (Figure S44). The above results suggest that the introduction of TTF into active metal complexes is really an effective approach to developing non‐noble catalysts for artificial photosynthesis.

A series of control experiments with [CoL^2^]^2+^ as a catalyst were carried out to thoroughly investigate this photocatalytic reaction of coupling CO_2_ reduction with CH_3_OH oxidation to HCOOH. First, the photocatalytic reactions were performed in the absence of [CoL^2^]^2+^, CH_3_OH, or illumination, no HCOOH was detected (Figure 1c), suggesting that [CoL^2^]^2+^, CH_3_OH, and light are all indispensable for the photocatalytic reaction to occur. Second, the photocatalytic reaction was carried out using Ar instead of CO_2_. The result shows that no HCOOH was generated either (Figure 1c), indicating that CO_2_ is the possible carbon source for HCOOH production. This conclusion was directly evidenced by the isotopic labeling experiment with ^13^CO_2_ instead of CO_2_ (see discussion below). No H_2_ was detected under Ar atmosphere. It may be attributed to the affinity between the cobalt site and the hydrogen atom, which significantly diminishes the likelihood of hydrogen–hydrogen binding, thereby inhibiting H_2_ evolution.^[^ ^56^ ^]^ Third, the ratio of CH_3_OH/H_2_O (*v*:*v*) was explored. It was found that the photocatalytic activity showed a volcano‐type with the ratio of CH_3_OH/H_2_O (Figure S45). When the ratio of CH_3_OH/H_2_O is 4:1, a maximum amount of HCOOH was obtained. Fourth, it was found that the yield of HCOOH increased with the concentration of the catalyst. At a catalyst concentration of 5 µM, the TON remained as high as 310 (Figure S46). Upon scaling up the catalytic system (from 5 to 30 mL total volume), the yield of HCOOH reached 20.1 µmol, consistent with a TON of 671 (Figure S47).

The excellent photocatalytic activity of [CoL^2^]^2+^ for coupling CO_2_ reduction with CH_3_OH oxidation was further evaluated under outdoor natural sunlight illumination at ambient conditions. The results show that the sunlight can also drive the coupling reactions of CO_2_ reduction and CH_3_OH oxidation over [CoL^2^]^2+^, with a maximal TON value of 207 to HCOOH upon illumination for 8 h by natural sunlight on 15th August, 2023 (Figures 1d, S48). Although the TON value for HCOOH (using [CoL^2^]^2+^) under sunlight is lower than that under the laboratory light source, it illustrates the excellent activity of [CoL^2^]^2+^ in photocatalytic CO_2_ reduction coupled with CH_3_OH oxidation.

**Figure 2:**
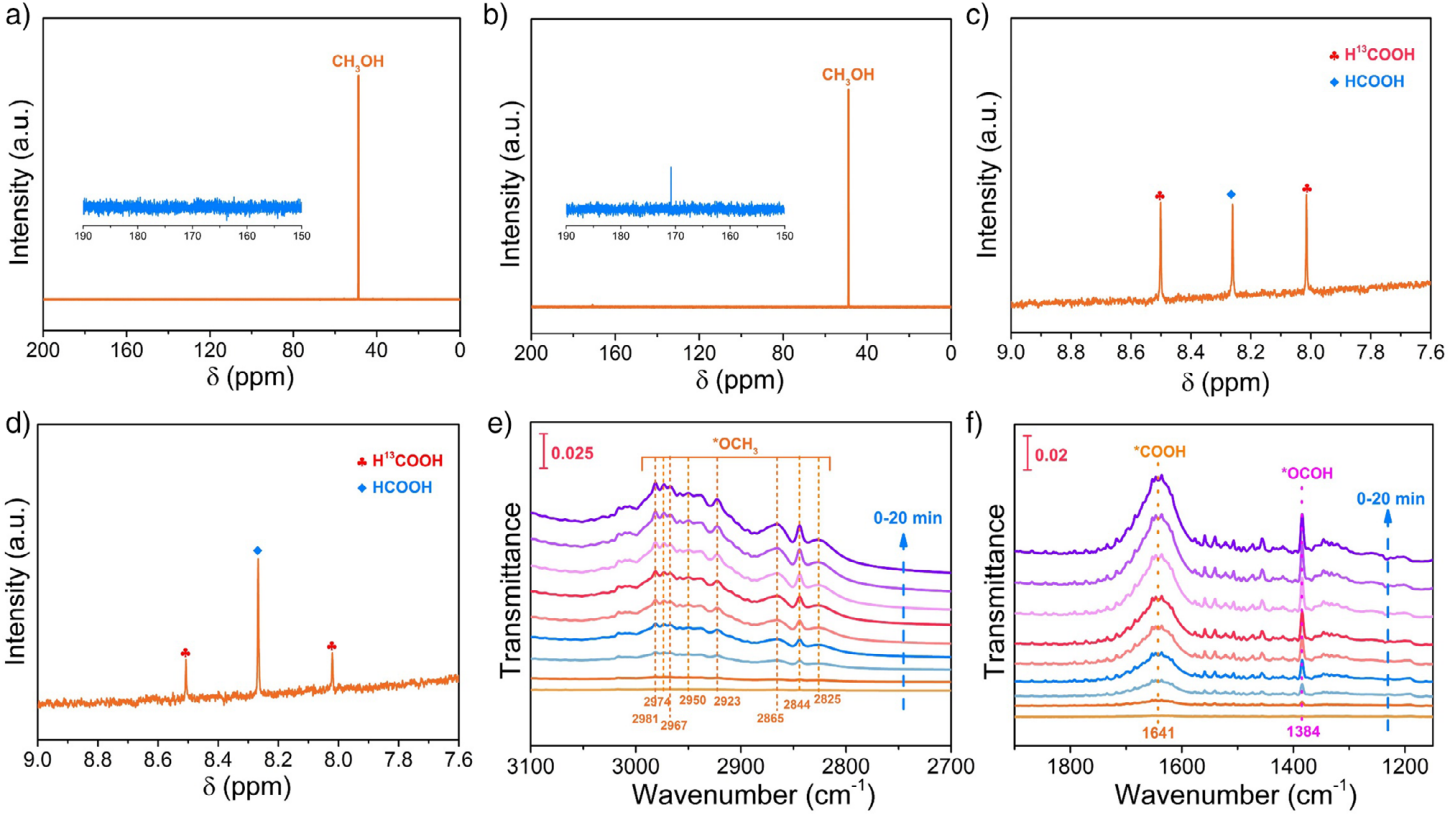
a) ^13^C NMR spectra of products generated in the photocatalytic system containing CO_2_ and CH_3_OH by [CoL^2^]^2+^; b) ^13^C NMR and c) ^1^H NMR spectra of products generated in the photocatalytic system containing ^13^CO_2_ and CH_3_OH by [CoL^2^]^2+^; d) ^1^H NMR spectra of products generated in the photocatalytic system containing CO_2_ and ^13^CH_3_OH by [CoL^2^]^2+^. Operando FTIR spectra of [CoL^2^]^2+^ during the photocatalytic CO_2_ reduction coupled with CH_3_OH oxidation, showing the characteristic absorption peaks of reaction intermediates with the increase of illumination time, e) 2700–3100 cm^−1^ and f) 1150–1900 cm^−1^.

To further demonstrate the coupling of CO_2_ photoreduction and CH_3_OH photooxidation, photocatalytic reaction with [CoL^2^]^2+^ under Ar was performed in the presence of 10 mM AgNO_3_. The result shows that 2.29 µmol HCOOH was generated (Figure S49). It should be noted that as mentioned above, no formic acid was formed in the control photocatalytic experiment under argon (Figure 1c). Thus, the generation of HCOOH in the presence of AgNO_3_ should be attributed to the CH_3_OH oxidation, in which the Ag^+^ serves as electron scavenger, benefiting to the CH_3_OH oxidation by the positive TTF moiety. This result shows that the half‐reaction of CH_3_OH photooxidation could be achieved by [CoL^2^]^2+^. On the other hand, the photocatalytic CO_2_ reduction reaction by [CoL^2^]^2+^ was further carried out using TEOA instead of CH_3_OH as the electron sacrificial reductant. A large amount of HCOOH was also generated and observed, indicating that the half‐reaction of CO_2_ photoreduction could also be realized by [CoL^2^]^2+^ (Figure S49). Isotopic labeling experiments were further conducted to confirm the origin of the generated HCOOH. As shown in Figure 2, in the presence of ^13^CO_2_ and CH_3_OH system, the ^13^C NMR spectrum shows the H^13^COOH signal at 170.8 ppm,^[^ ^57^, ^58^ ^]^ confirming that HCOOH originates from CO_2_ reduction (Figure 2a,b). In addition, the ^1^H NMR spectrum displays two splitted peaks at *δ* = 8.01 and 8.50 ppm respectively (Figure 2c), which can be assigned to the H^13^COOH generated from ^13^CO_2_ reduction.^[^ ^58^ ^]^ Meanwhile, a typical peak at *δ* = 8.25 ppm appears, which can be identified as HCOOH originating from CH_3_OH oxidation. Moreover, the integrated area ratio of peaks belonging to H^13^COOH and HCOOH is about 2:1, which is consistent with the stoichiometric ratio (CO_2_ + 2e^−^ + 2H^+^ → HCOOH; CH_3_OH + H_2_O + 4h^+^ → 4H^+^ + HCOOH). To further verify the product of CH_3_OH oxidation, the photocatalytic experiment by [CoL^2^]^2+^ with CO_2_ and ^13^CH_3_OH as feedstocks was performed. As shown in Figure 2d, both the doublet associated with H^13^COOH and singlet assigned to HCOOH can also be observed in the ^1^H NMR spectrum, and the integrated area ratio of peaks originated from CO_2_ reduction and CH_3_OH oxidation can be calculated to be about 2:1. The results of isotopic labeling experiments clearly show that the generated HCOOH is from both CO_2_ reduction and CH_3_OH oxidation, which also confirms that CO_2_ photoreduction and CH_3_OH photooxidation are simultaneously achieved by [CoL^2^]^2+^.

The reaction intermediates during CO_2_ reduction coupled with CH_3_OH oxidation using [CoL^2^]^2+^ were monitored by Fourier transform infrared (FTIR) spectroscopy. As shown in Figure 2e,f, new peaks appeared under photoirradiation, and their intensities gradually increased with the illumination time from 0 to 20 min. The peaks at 2981, 2974, 2967, 2950, 2923, 2865, 2844 and 2825 cm^−1^ may be assigned to the stretching and bending vibration of C─H in *OCH_3_, deciphering a multistep dehydrogenation process of the CH_3_OH oxidation over [CoL^2^]^2+^.^[^ ^59^ ^]^ In addition, the reaction intermediates in the process of CO_2_ reduction over [CoL^2^]^2+^ were also monitored. The peak located at 1641 cm^−1^ can be attributed to a *COOH intermediate (C atom binding to the Co site),^[^ ^60^ ^]^ which indicates the CO_2_ undergoes first proton‐coupled electron transfer (PCET) process to form the *COOH intermediate. In addition, another gradually enhanced peak at 1384 cm^−1^ can be assigned to the *OCOH,^[^ ^61^ ^]^ implying the *COOH transforms to *OCOH (O atom binding to the Co site) through isomerization, the latter being a key intermediate in CO_2_‐to‐HCOOH conversion. Of note, no peaks related to either *OCH_3_, *COOH, or *OCOH intermediate were detected in the absence of CH_3_OH or CO_2_ (Figures S50, S51). These FTIR results add support to the conclusion that photocatalytic coupling reaction of CO_2_ reduction with CH_3_OH oxidation occurs with [CoL^2^]^2+^ as catalyst.

In order to get insights in the reaction mechanism, the electrochemical behaviors of [CoL^1^]^2+^ and [CoL^2^]^2+^ were investigated by cyclic voltammetry (CV) in CH_3_OH/H_2_O (*v*:*v* = 4:1) under the Ar and CO_2_ atmosphere. As shown in Figure S52, irreversible reduction peaks at −1.0 V versus (vs.) normal hydrogen electrode (NHE) for [CoL^1^]^2+^, and −0.71 versus NHE for [CoL^2^]^2+^ were observed, which corresponds to the reduction of Co^II^ to Co^I^. These values are more negative than the theoretical CO_2_ reduction potential (CO_2_/HCOOH, −0.61 V vs. NHE). In addition, the catalytic current for [CoL^1^]^2+^ and [CoL^2^]^2+^ in the presence of CO_2_ is obviously enhanced compared to those in Ar, manifesting their potential catalytic activity for CO_2_ reduction. Furthermore, [CoL^2^]^2+^ exhibits two redox waves assignable to the redox processes of TTF/TTF^+^ and TTF^+^/TTF^2+^ at +0.92 and +1.23 V, respectively (Figure S53), which is more positive than the theoretical oxidation potential of CH_3_OH to HCOOH (0.10 V vs. NHE).^[^ ^62^ ^]^ These redox properties imply that it is thermodynamically feasible for [CoL^2^]^2+^ to produce HCOOH via CO_2_ reduction and CH_3_OH oxidation.

**Figure 3:**
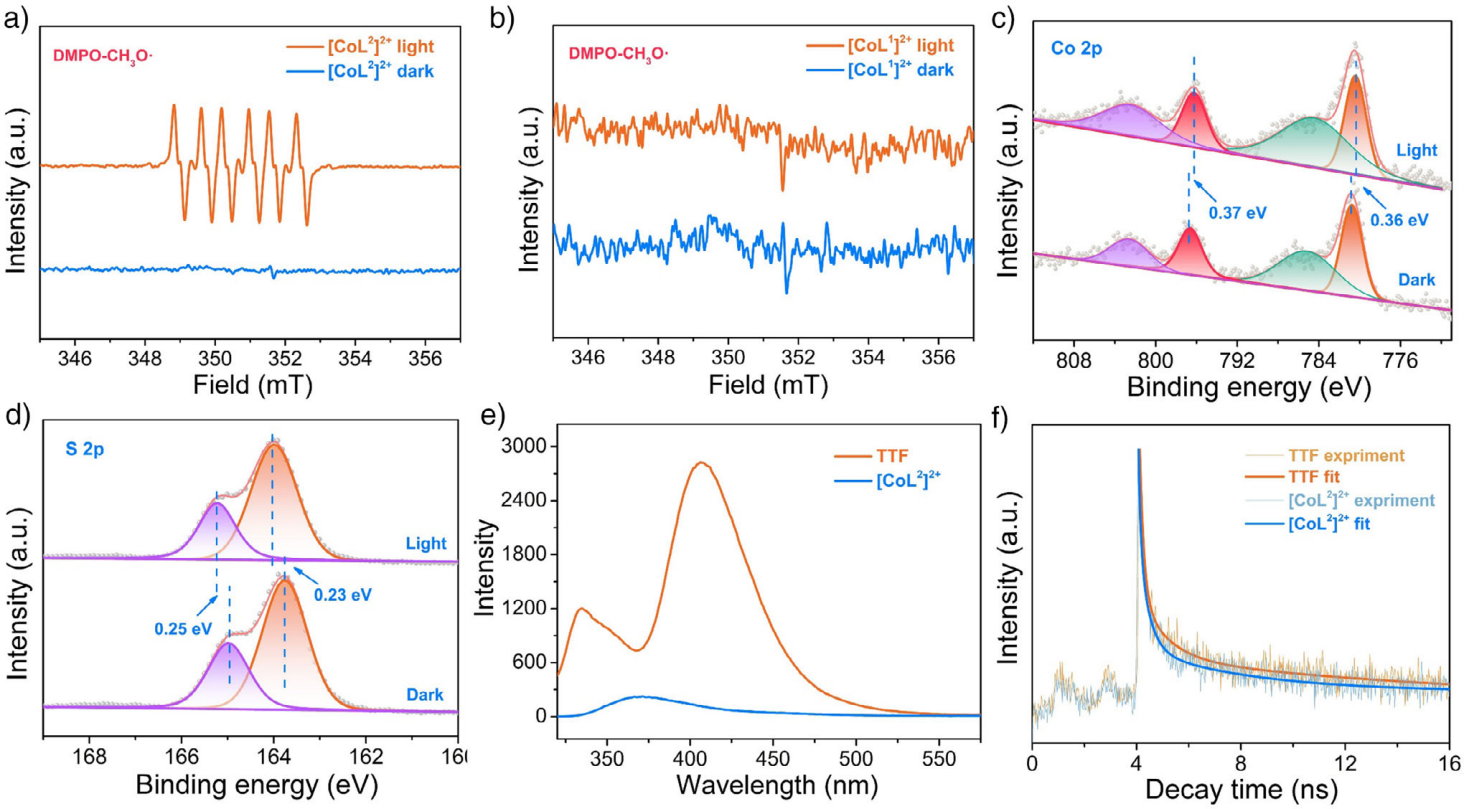
EPR signals of a) [CoL^2^]^2+^ and b) [CoL^1^]^2+^ under the dark and light illumination in the presence of DMPO as the spin‐trapping reagents; high‐resolution XPS spectra of c) Co 2p and d) S 2p in [CoL^2^]^2+^ in dark and upon illumination by a Xe lamp; e) PL spectra of TTF (100 µM) and [CoL^2^]^2+^ (100 µM) in CH_3_OH solution; f) the time‐resolved photoluminescence (TRPL) spectra of TTF and [CoL^2^]^2+^.

Electron paramagnetic resonance (EPR) of [CoL^2^]^2+^ was performed to better uncover the electron‐transfer process. The EPR spectrum of [CoL^2^]^2+^ shows a strong signal at *g* = 2.13 in the dark, which is ascribed to the signal of paramagnetic Co(II) (Figure S54).^[^ ^63^ ^]^ Upon light illumination, the signal of Co(II) displays evident decrease, indicating that part of Co(II) accepts an electron from TTF moiety to form diamagnetic Co(I) (Table S2).^[^ ^64^, ^65^ ^]^ In addition, the EPR measurement was further performed in CH_3_OH to detect radical species during photocatalytic experiments using 5,5‐dimethyl‐1‐pyrroline *N*‐oxide (DMPO) used as a spin‐trap agent. As shown in Figure 3a, a typical six characteristic signals for DMPO‐CH_3_O**·** originating from CH_3_OH oxidation were markedly observed with [CoL^2^]^2+^ upon illumination,^[^ ^66^ ^]^ while no signal of DMPO‐CH_3_O**·** can be detected with [CoL^1^]^2+^ (Figure 3b), indicating that the methanol oxidation process occurs due to the introduced group of TTF moiety. The electron transfer pathway in [CoL^2^]^2+^ was further investigated by in‐situ light‐illuminated XPS measurements. As shown in Figure 3c,d, the [CoL^2^]^2+^ exhibited two peaks at 780.78 and 796.62 eV in dark conditions, which can be assigned to the Co 2p_3/2_ and Co 2p_1/2_, respectively.^[^ ^67^ ^]^ Upon light illumination, the Co 2p binding energy displayed a slightly negative shift (0.36–0.37 eV; Figures 3c, S55), suggesting an increase in the electron density at the Co, which is consistent with the EPR result. Meanwhile, the binding energy of S 2p_3/2_ and S 2p_1/2_ at 163.76 and 164.98 eV respectively with [CoL^2^]^2+^ in dark conditions underwent markedly positive shift (0.23–0.25 eV) upon light illumination (Figures 3d, S55),^[^ ^52^ ^]^ which indicates a decrease in the electron density on the TTF groups. The results of the XPS and EPR measurements provide solid evidence that the electron migrates from the TTF groups to Co(II) in [CoL^2^]^2+^, and the Co(II) and TTF serve as reduction and oxidation sites, respectively.^[^ ^68^ ^]^ To reveal more details about the catalytic mechanism of [CoL^2^]^2+^ in photochemical CO_2_ reduction, luminescence quenching experiments of the excited state TTF* were performed with the addition of [CoL^1^]^2+^ or CH_3_OH. As shown in Figure S56, TTF exhibits strong fluorescence at 412 nm upon excitation at 300 nm. The fluorescent intensity of TTF dramatically decreased with the gradual addition of [CoL^1^]^2+^. By contrast, the addition of CH_3_OH did not induce significant quenching to the excited TTF*. These observations illustrate that the quenched mode of TTF* can be assigned to an oxidative quenching mechanism.

The photoluminescence (PL) and time‐resolved photoluminescence (TRPL) spectra were further performed with [CoL^2^]^2+^. As shown in Figure 3e, the PL spectrum of TTF exhibits a strong characteristic peak at 406 nm, while the luminescence intensity of [CoL^2^]^2+^ is significantly smaller than that of TTF. This indicates that the [CoL^2^]^2+^ can competitively promote charge transfer from its excited state. Additionally, the TRPL spectra revealed a reduced average exciton lifetime of 2.5 ns for TTF to 0.9 ns for [CoL^2^]^2+^, also illustrating that [CoL^2^]^2+^ can accelerate the charge transfer (Figure 3f). The UV–vis absorption spectra of TTF show an absorption peak at 310 nm, and after irradiation for 14 h with an Xe lamp, photodegradation of TTF was observed (Figure S57). However, the [CoL^2^]^2+^ shows better robustness, as the main absorption peak of [CoL^2^]^2+^ can still be observed after 14 h illumination (Figure S27). The enhanced durability of TTF when inserted in [CoL^2^]^2+^ can be attributed to the rapid electron transfer from TTF to Co(II) center, and further reduction by CH_3_OH, which avoids overoxidation of TTF itself. Furthermore, the HOMO‐LUMO gaps of [CoL^1^]^2+^ and [CoL^2^]^2+^ were calculated to be 3.17 and 0.84 eV, respectively (Figures S58, S59), indicating that the introduction of the TTF group reduces the HOMO‐LUMO gap, thereby accelerating electron transfer during the photocatalytic production of HCOOH.^[^ ^69^, ^70^ ^]^ A similar trend was observed in [NiL^2^]^2+^ and [CuL^2^]^+^ (Figures S60, S61). Moreover, the CV of [NiL^2^]^2+^ and [CuL^2^]^+^ shows a primary reduction peak at −0.77 and −0.94 V versus NHE (Figure S62), respectively. These values are more negative than [CoL^2^]^2+^ (‐0.71 V vs. NHE), illustrating that [CoL^2^]^2+^ possesses higher photocatalytic activity over [NiL^2^]^2+^ and [CuL^2^]^+^,^[^ ^71^ ^]^ which well supports the experimental results.

Based on the above results, a reaction scheme for the photocatalytic CO_2_ reduction coupled with CH_3_OH oxidation using [CoL^2^]^2+^ as catalyst can be proposed (Figure S63). The photoactive TTF moiety in [CoL^2^]^2+^ is first activated under light illumination to an excited state TTF*, which is oxidatively quenched by electron transfer to the Co(II) center. The resulting Co(I) further binds the CO_2_ molecule. The CO_2_ molecule is thus activated to *CO_2_, which undergoes a PCET process to form *COOH intermediate. The *COOH isomerizes and finally generates HCOOH. At the same time, the positive TTF moiety activates the O─H bond of CH_3_OH to generate *OCH_3_ intermediate, which undergoes multistep dehydrogenation and oxygen‐coupled processes to generate the oxidation product of HCOOH.

**Figure 4:**
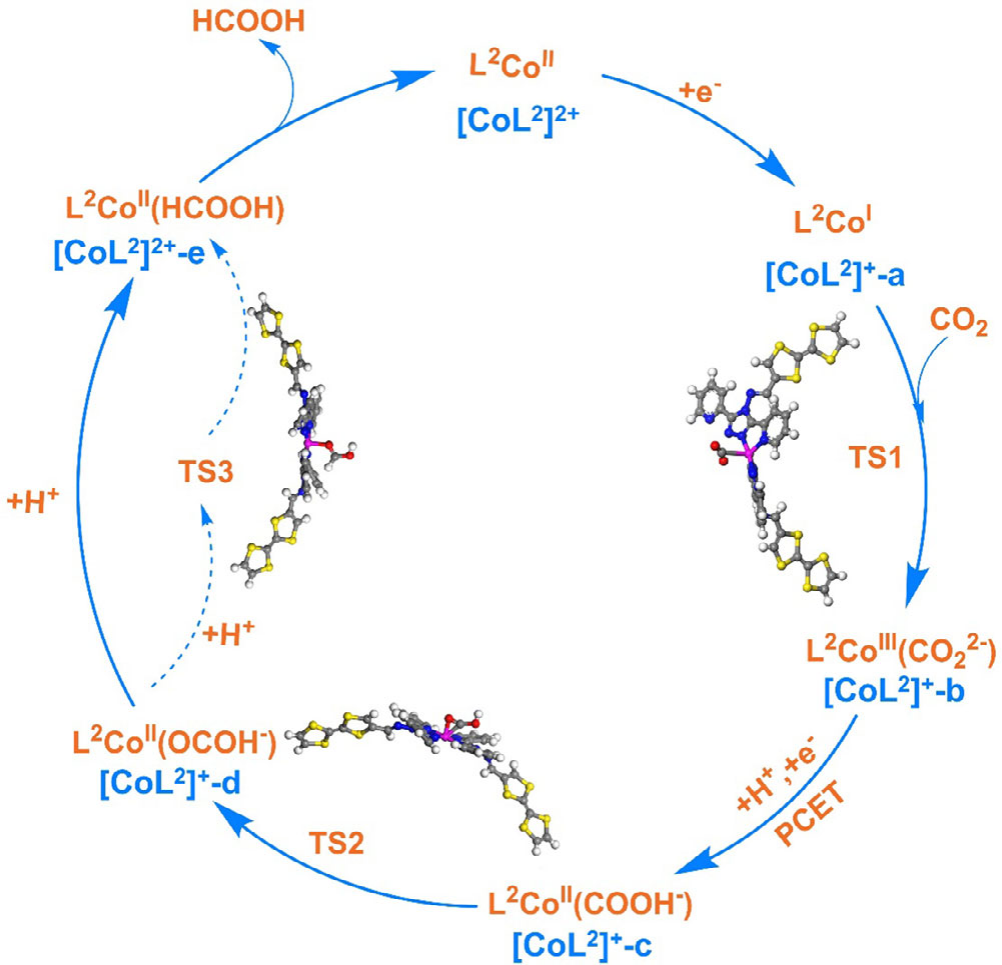
Proposed catalytic mechanism for the reduction of CO_2_ to HCOOH with [CoL^2^]^2+^.

The catalytic mechanism of [CoL^2^]^2+^ for photocatalytic CO_2_ reduction to HCOOH has been further investigated at a molecular level by DFT calculations (see the Supporting Information, Figures S64, S65). After detailed calculations, a reasonable reaction pathway has been presented in Figure 4a. First, [CoL^2^]^2+^ obtains one electron to generate [CoL^2^]^+^‐a, during which the Co^II^ in [CoL^2^]^2+^ is reduced to Co^I^. Second, the Co^I^ in [CoL^2^]^+^‐a captures a CO_2_, and then the CO_2_ accepts 2e^−^ from Co^I^ to be reduced to CO_2_^2−^ through the transition state of TS1 to generate [CoL^2^]^+^‐b. The energy barrier for this transition state is 10.61 kcal mol^−1^ (Figures S64, S65). Third, [CoL^2^]^+^‐b goes through a PCET process to generate [CoL^2^]^+^‐c. Fourth, the ‐COOH in [CoL^2^]^+^‐c transforms to ─OCHO through the transition state of TS2 to generate [CoL^2^]^+^‐d. The energy barrier calculated for TS2 is 10.61 kcal mol^−1^. Finally, [CoL^2^]^+^‐d is protonated to yield [CoL^2^]^2+^‐e via transition state of TS3. The energy barrier calculated for TS3 is 8.76 kcal mol^−1^. After the release of HCOOH in [CoL^2^]^2+^‐e, [CoL^2^]^2+^ is regenerated and the photocatalytic cycle restarts. This mechanism is similar to that reported by Chen et al.^[^ ^72^ ^]^

## Conclusion

In summary, we designed innovative self‐photosensitizing non‐noble single metal complexes, which achieve the photocatalytic CO_2_ reduction coupled with CH_3_OH oxidation to simultaneously synthesize formic acid, without the addition of any photosensitizers and sacrificial reductants. The spectroscopic studies and DFT calculations revealed that integrating reduction and oxidation moieties in one complex promotes charge transfer kinetics, thus enhancing the activity of CO_2_ reduction coupled with CH_3_OH oxidation. This work opens new perspectives toward the development of molecular catalysts for artificial photosynthesis.

## Conflict of Interests

The authors declare no conflict of interest.

## Supporting information



Supporting Information

## Data Availability

The data that support the findings of this study are available from the corresponding author upon reasonable request.
