# Supplementary material for: Self‐Photosensitizing Cobalt Complexes for Photocatalytic CO2 Reduction Coupled with CH3OH Oxidation
Source: Angew Chem Int Ed Engl. 2025 Apr 27;64(26):e202506060. doi: 10.1002/anie.202506060 (PMC12184315; doi:10.1002/anie.202506060)
Supplement: Supplementary file 1 — Supporting Information [file ANIE-64-e202506060-s001.doc]

Supporting Information
©Wiley-VCH 2024
69451 Weinheim, Germany

**Self-photosensitizing Cobalt Complexes for Photocatalytic CO_2_ Reduction Coupled with CH_3_OH Oxidation**

**Ji-Hong Zhang,^1,2,#^ Zhao-Ming Ge,^1,#^ Di-Chang Zhong,^1^****^,*^ Jing-Lin Zuo,^3^ Marc Robert,^4,5,*^ Tong-Bu Lu^1,*^**

**Section 1. Materials and Instrumentation**

All chemicals and materials were commercially obtained and used without further purification. Solvents were dried and distilled before used for ligand synthesis. The purity of N_2_, Ar and CO_2_ were 99.999%. UV-Vis absorption spectra were recorded with a U-3900 UV/VIS spectrophotometer (Hitachi). NMR spectra were recorded on a Bruker 400 MHz instrument. Steady-state photoluminescence (PL) spectra were measured using a F-4600 Fluorescence spectrophotometer (Hitachi). Time-resolved photoluminescence decays were detected with a FLS-1000 steady state and transient state fluorescence spectrometer (Edinburgh Instruments Ltd.). X-ray photoelectron spectroscopy (XPS) measurements were carried out by an ESCALAB250Xi X-ray photoelectron spectrometer with Al Kα as the excitation source (Thermo scientific). Gas chromatograph measurements were analyzed in a GC-2014 Gas Chromatograph instrument (SHIMADZU) equipped with TCD and FID dual detectors. Ion chromatography (IC) was taken on a DX-600 chromatographic instrument (Dionex). Electron paramagnetic resonance (EPR) spectra were measured on EMXplus-6/1 (Bruker, Germany).

**Section 2. Experimental Section**

**Synthesis of 4-Amino-3,5-bis(pyridin-2-yl)-1,2,4-triazole (L^1^)**

L^1^ was synthesized according to the literature procedure^1,2^. A mixture of 2-cyanopyridine (10.4 g, 0.1 mol), hydrazine dihydrochloride (10.5 g, 0.1 mol) and hydrazine hydrate (15 g, 0.3 mol) in diethylene glycol (50 mL) was heated at 130 ℃ with stirring under nitrogen for 6 hours. After cooling, the reaction mixture was diluted with distilled water (100 mL). The precipitate was obtained and filtered, washed with water, and recrystallized in ethanol. Yield: 80%. ^1^H NMR (400 MHz, DMSO-*d_6_*): δ (ppm): 8.77 (d, J = 4.3 Hz, 2H), 8.23 (d, J = 7.9 Hz, 2H), 8.07 (t, J = 7.8 Hz, 2H), 7.83 (s, 2H), 7.62-7.54 (m, 2H).

**Synthesis of** **TTF-CHO**

TTF-CHO was synthesized according to the literature method^3^. Tetrathiafulvalene (TTF, 1.00 g, 4.9 mmol) was dissolved in anhydrous THF (60 mL) under an inert atmosphere. The reaction mixture was cooled to -78℃ with an N_2_ bath, then lithium diisopropylamide (LDA, 3.13 mL, 1.8m) was added and stirred for 90 min to obtain the lithiated TTF derivative. After that, anhydrous N,N-dimethylformamide (DMF, 1.20 mL, 15.5 mmol) was added to the solution, which was further stirred for 2 h. The mixture was then acidified with hydrochloric acid (2 M), extracted with CH_2_Cl_2_, and washed with water. The organic phase was dried with anhydrous magnesium sulfate, filtered, and the solvent was evaporated under reduced pressure. The product was purified by column chromatography on silica gel using a mixture of hexane and CH_2_Cl_2_ as eluent (1:1). 0.79 g (69%) of the final product were obtained after solvent evaporation. ^1^H NMR (400 MHz, CDCl_3_): δ (ppm): 9.48 (s, 1H); 7.43 (s, 1H); 6.34 (s, 2H).

**Synthesis of (*E*)-1-(****[2,2'-bi(1,3-dithiolylidene)]-4-yl)-*N*-(3,5-di(pyridin-2-yl)-4*H*-1,2,4-triazol-4-yl) methanimine (L^2^)**

To a stirred methanol solution (20 mL) containing L^1^ (238 mg, 1 mmol) and TTF-CHO (278 mg, 1.2 mmol), acetic acid (200 μL) was added under argon. The reaction mixture was refluxed for 12 h and then cooled to room temperature. The solvent was evaporated under reduced pressure, washed with cold methanol and then recrystallized from hot ethanol. Yield: 90%. ^1^H NMR (400 MHz, CDCl_3_): δ (ppm): 8.77 (s, 1H), 8.67 (d, J = 4.8 Hz, 2H), 8.24 (d, J = 8.2 Hz, 2H), 7.92 (t, J = 7.9 Hz, 2H), 7.43 (d, J = 6.1 Hz, 2H), 7.22 (s, 1H), 6.34 (s, 2H).

**Synthesis of [CoL^1^]^2+^**

[CoL^1^]^2+^ was synthesized according to the literature method^4^. Sodium hexadecane sulfonate (0.657 g, 2.00 mmol) was dissolved in hot distilled water (150 mL; 70 ℃), giving a colorless solution, to which solid cobalt(II) chloride hexahydrate (0.238 g, 1.00 mmol) was added, resulting in a small amount of pale pink precipitate. The mixture was further heated at 70 ℃ for 15 minutes and then cooled to 4 ℃, resulting in a large amount of pale pink precipitate. The precipitate was isolated by filtration, washed with cold distilled water (20 mL) and dried thoroughly over phosphorus pentoxide in vacuum. The Co^II^(H_2_O)_2_(C_16_SO_3_)_2_ were obtained. The Co^II^(OH_2_)_2_(C_16_SO_3_)_2_ (106 mg, 0.15 mmol) and L^1^ (71 mg, 0.30 mmol) were dissolved in methanol (8 mL), giving a clear pale orange solution. This was left to slowly evaporate, affording large pale orange block crystals, which were isolated by filtration, washed with ice-cold methanol (2 mL) and dried in a vacuum oven. Yield: 80%. C_58_H_94_CoN_12_O_8_S_2_: calc. C 57.55, H 7.83, N 13.89; found C 56.78, H 7.86, N 13.72.

**Synthesis of [CoL^2^]^2+^**

A CH_3_CH_2_OH solution (4 mL) of Co(NO_3_)_2_·6H_2_O (29.1 mg, 0.1 mmol) was added dropwise to a CH_2_Cl_2_ solution (4 mL) of L^2^ (90.4 mg, 0.2 mmol). Then, the mixture was stirred at room temperature for 2 h. The products were obtained by filtration, washed with CH_2_Cl_2_ and dried in a vacuum oven. Yield: 75%. C_38_H_26_CoN_14_O_7_S_8_: calc. C 41.26, H 2.37, N 17.73; found C 40.68, H 2.49, N 17.50.

**Synthesis of** **[NiL^2^]^2+^ and [CuL^2^]^+^**

The procedures for preparing [NiL^2^]^2+^ and [CuL^2^]^+^ were similar to that of [CoL^2^]^2+^, except using Ni(NO_3_)_2_·6H_2_O and Cu(NO_3_)_2_·3H_2_O instead of Co(NO_3_)_2_·6H_2_O, respectively. Yield: ~83%. C_38_H_26_NiN_14_O_7_S_8_: calc. C 41.27, H 2.37, N 17.73; found C 40.62, H 2.51, N 17.46. C_38_H_26_CuN_13_O_4_S_8_: calc. C 43.52, H 2.50, N 17.36; found C 42.76, H 2.63, N 17.03.

**Section 3. Photocatalytic CO_2_ reduction and CH_3_OH oxidation experiments.**

The photocatalytic reaction of CO_2_ reduction concurrently with CH_3_OH oxidation was performed in a 17.5 mL quartz reactor containing a catalyst, and 5 mL CH_3_OH/H_2_O solution (*v/v* = 4:1). The mixture was bubbled with argon for 30 min and then with CO_2_ for another 30 min. The photocatalytic reaction was initiated upon irradiation by a 300 W Xe lamp (320 < λ < 780 nm, Microsolar300, Beijing Perfectlight), with the light intensity of 200 mW cm^-2^. The generated gases were analyzed by a gas chromatography, and the possible products in the solution were analyzed by ion chromatography. Calibration curves for formate ions were established before each analysis.

**Section 4. Quantum yield determination for CO_2_ reduction.**

A mixed solution of CH_3_OH/H_2_O (*v/v* = 4:1, 5 mL) containing [CoL^2^]2+/[NiL^2^]2+/[CuL^2^]^+^ (1μM) was irradiated at 298 K using a LED light (λ = 365 nm, 100 mW·cm^-2^, irradiation area of 0.4 cm^2^). The generated HCOOH was analyzed and quantified by ion chromatography. Quantum yield was calculated according to the following equation:

*Φ*_HCOOH_ = [2 × (2/3) × (number of the produced molecules)/(number of incident photons)] × 100%.

**Section 5. Electrochemical measurements.**

Electrochemical experiments were performed with an electrochemical workstation CHI 760E, using a glassy carbon working electrode (0.07 cm^2^, a Pt wire auxiliary electrode, and an Ag/AgNO_3_ (0.1 M) reference electrode in 1 μM NBu_4_PF_6_ CH_3_OH/H_2_O solution (*v/v* = 4:1). The glassy carbon electrode was polished with 0.3 and 0.05 μm Al_2_O_3_ slurry for 3 min to obtain a mirror surface, followed by sonication in distilled water for ~30 seconds to remove debris and was thoroughly rinsed with Milli-Q ultrapure water. The electrolyte solution was saturated by bubbling with Ar or CO_2_ for 15 min prior to each experiment.

**Section 6. HCHO** **detection methods.**

HCHO was analyzed through the colorimetric method. Typically, ammonium acetate (25 g), acetic acid (1 mL) and pentane-2,4-dione (0.2 mL) were dissolved in 100 mL water to form a color-developing reagent solution. Then 1 mL of the reaction liquid was mixed with 1 mL reagent solution. The mixed solution was placed in the water bath (50 °C) for 20 min, followed by measuring its UV-Vis absorption spectrum to detect quantitatively the concentration of HCHO based on the standard curve (Figure S20).

**Section 7. Operando FTIR Spectroscopy Measurements.**

Operando FTIR spectra were performed at room temperature on a Nicolet iS50 FT-IR (Thermo scientific) spectrometer equipped with an MCT detector cooled with liquid nitrogen, and one of the in-situ pool (CRCP-7070-CLT) was purchased from Tianjin Xianquan. During the test, UV light was used as excitation light. 100 mg of KBr and 1 mg of [CoL^2^]^2+^ were mixed and thoroughly ground under a baking lamp, and finally pressed into thin slice using for testing. Before in situ FTIR measurement, the apparatus was degassed and filled with CO_2_, H_2_O and CH_3_OH vapor for 20 min to remove air (the flow rate of CO_2_ was 10 mL min^-1^). Furthermore, the background peak was recorded after 30 min of system stabilization. Subsequently, the light source was turned on and the Operando FTIR spectrum of the sample was recorded.

**Section 8. In-situ Irradiated X-ray Photoelectron Spectroscopy (ISI-XPS) measurements.**

The samples were uniformly spread out on the silicon after dried at 60 °C for 12 h in an oven, and then characterized by X-ray photoelectron spectrometer (ESCALAB250Xi) with Al Kα as the excitation source and equipped with a 300 W Xe lamp (CEL-HXF300, CEAULICHT). The light source was placed about 50 cm away from the as-prepared samples. All spectra were obtained after being corrected by the C 1s signal of adventitious carbon. The elemental binding energy was fitted and analyzed using XPS Peak Fit 4.1 software, and meantime ensured the same for half peak width of characteristic peaks of the same elements.

**Section 9. In-situ Electron Paramagnetic Resonance (EPR) Measurements.**

A Bruker Electron Paramagnetic Resonance (EPR) spectrometer (Bruker, EMXplus-6/1, Germany) equipped with a 300 W Xe lamp (320 < λ < 780 nm, CEL-HXF300, CEAULICHT) was used for EPR measurements. The following settings were applied: centerfield, 3500 G; sweepwidth, 4500 G; poweratten, 30.0 dB; modulation frequency, 100 kHz; modulation amplitude, 2.0 G; sweep time, 60 s. A capillary tube containing a catalyst containing certain mass of [CoL^2^]^2+^ was placed in the corresponding position of the instrument, and the data was collected after being irradiated for 300 s.

**Section 10. Electron Paramagnetic Resonance (EPR) measurements of methoxy radical detection.**

5,5-dimethyl-1-pyrroline-N-oxide (DMPO) was selected as the trapping agent during the measurements. 5 mL of the methanol solution containing 100 μM [CoL^1^]^2+^ or [CoL^2^]^2+^ was mixed with 100 μL of DMPO trapping agent solution, after being irradiated for 300 s, the mixed solution was characterized by an electron spin resonance spectrometer (EMXplus-6/1, Bruker).

**Section 11. XAFS analysis.**

The EXAFS spectra were obtained by subtracting the post-edge background from the overall absorption and then normalizing with respect to the edge-jump step. Subsequently, the χ(k) data of were Fourier transformed to real (R) space using a hanning windows (dk = 1.0 Å^-1^) to separate the EXAFS contributions from different coordination shells. To obtain the quantitative structural parameters around central atoms, least-squares curve parameter fitting was performed using the ARTEMIS module of IFEFFIT software packages.

The following EXAFS equation was used:

*S_0_^2^* is the amplitude reduction factor, *F_j_(k)* is the effective curved-wave backscattering amplitude, *N_j_* is the number of neighbors in the *j^th^* atomic shell, *R_j_* is the distance between the X-ray absorbing central atom and the atoms in the *j^th^* atomic shell (backscatterer), *λ* is the mean free path in Å, ϕ *_j_(k)* is the phase shift (including the phase shift for each shell and the total central atom phase shift), *σ_j_* is the Debye-Waller parameter of the *j^th^* atomic shell (variation of distances around the average *R_j_*). The functions *F_j_(k)*, *λ* and ϕ *_j_(k)* were calculated with the ab initio code FEFF8.2. The additional details for EXAFS simulations are given below.

The coordination numbers of model samples were fixed as the nominal values. The obtained *S_0_^2^* was fixed in the subsequent fitting. While the internal atomic distances *R*, Debye-Waller factor *σ^2^*, and the edge-energy shift *ΔE_0_* were allowed to run freely.

**Section 12. Density functional theory (DFT) calculations.**

All density functional theory (DFT) calculations were carried out using the Gaussian 09 program^5^. Geometries of intermediates and transition states were optimized using the dispersion-corrected B3LYP-D3 functional with basis set of Lanl2dz for all atoms in the gas phase. Vibrational frequency calculations were performed for all stationary points to confirm if each optimized structure is a local minimum or a transition state structure. All optimized transition state structures have only one imaginary (negative) frequency, and all minima (reactants, products, and intermediates) have no imaginary frequencies.


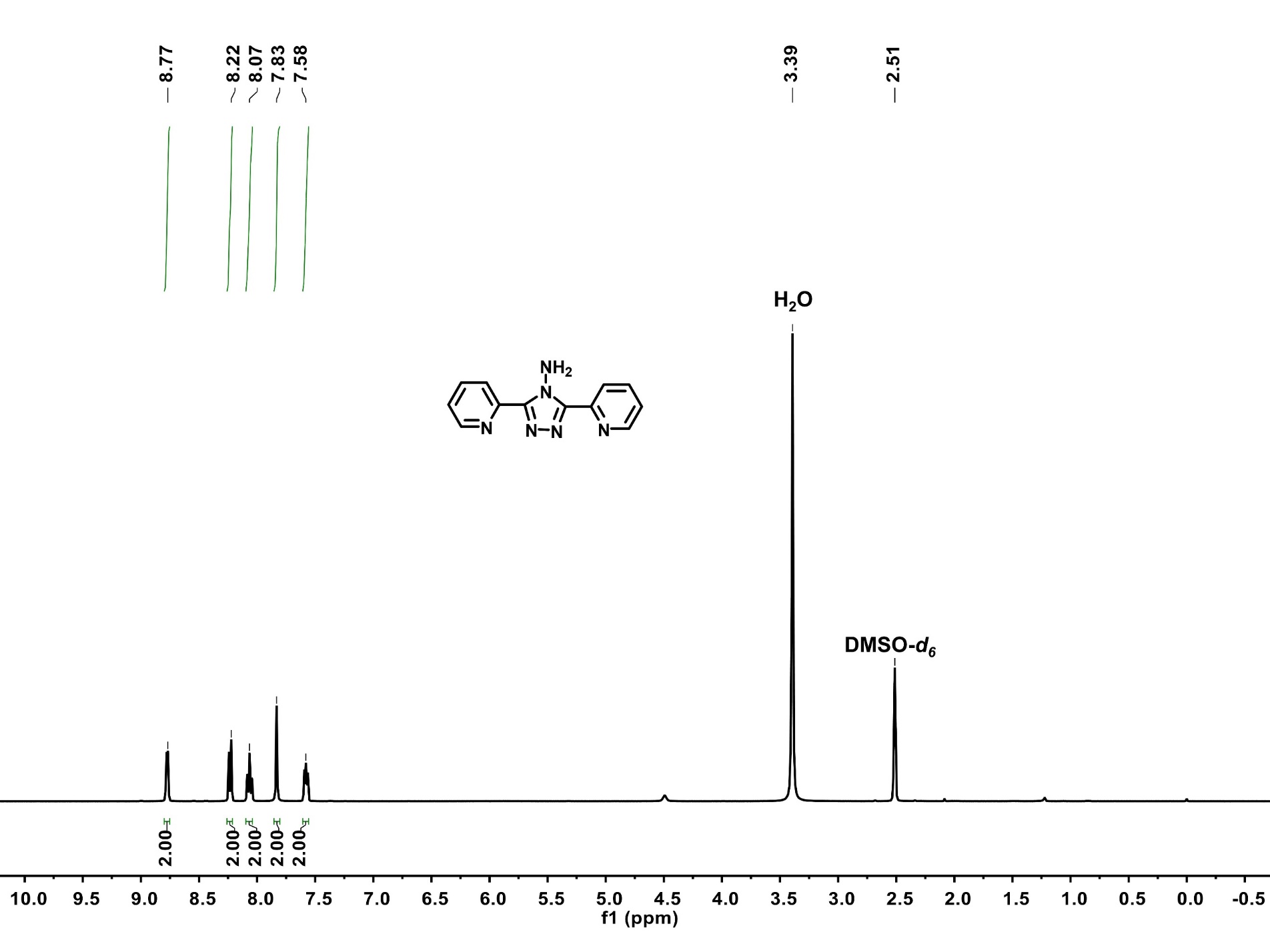


**Figure S1**. ^1^H NMR spectra of L^1^.


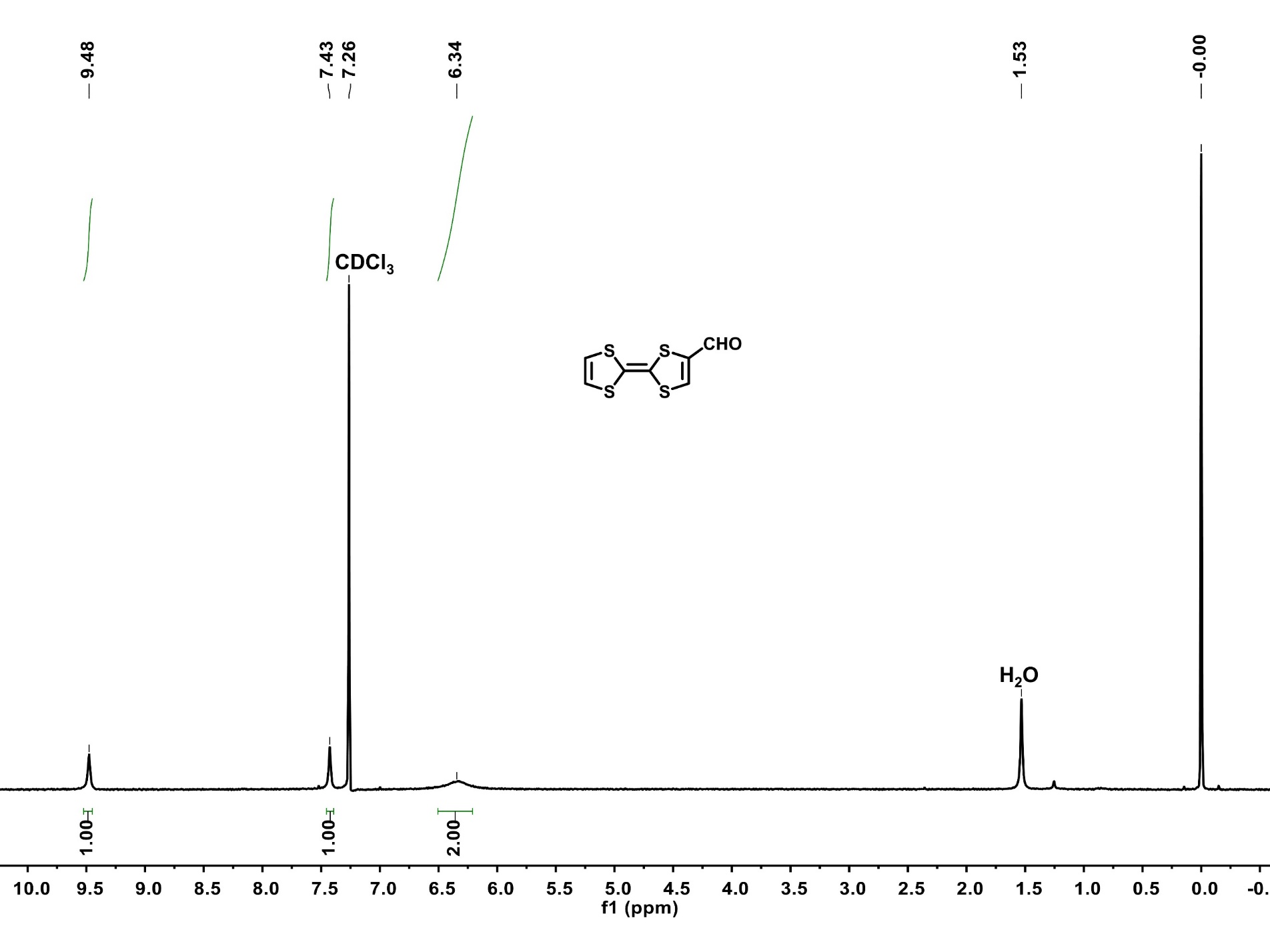


**Figure S2**. ^1^H NMR spectra of TTF-CHO.


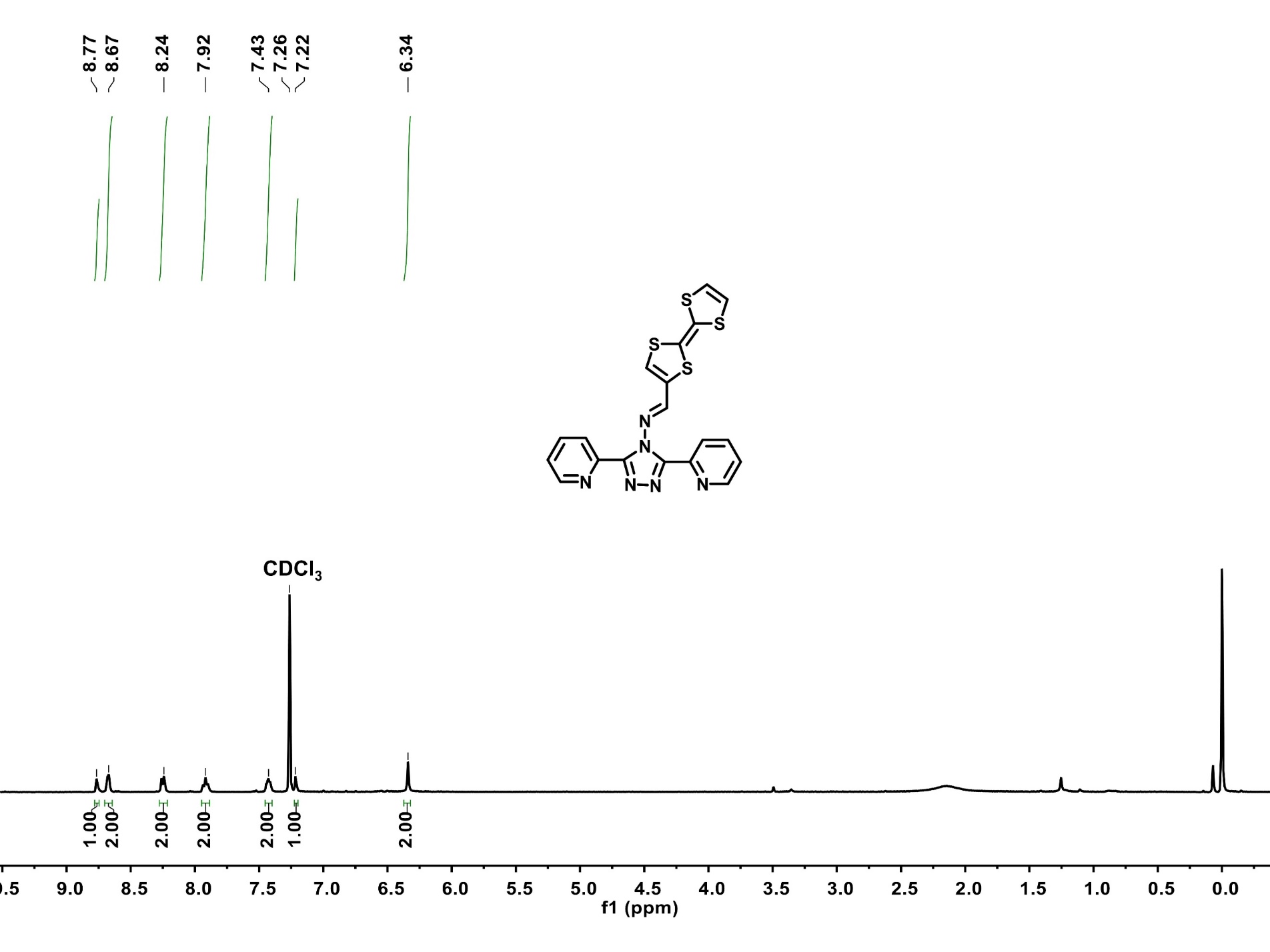


**Figure S3**. ^1^H NMR spectra of L^2^.


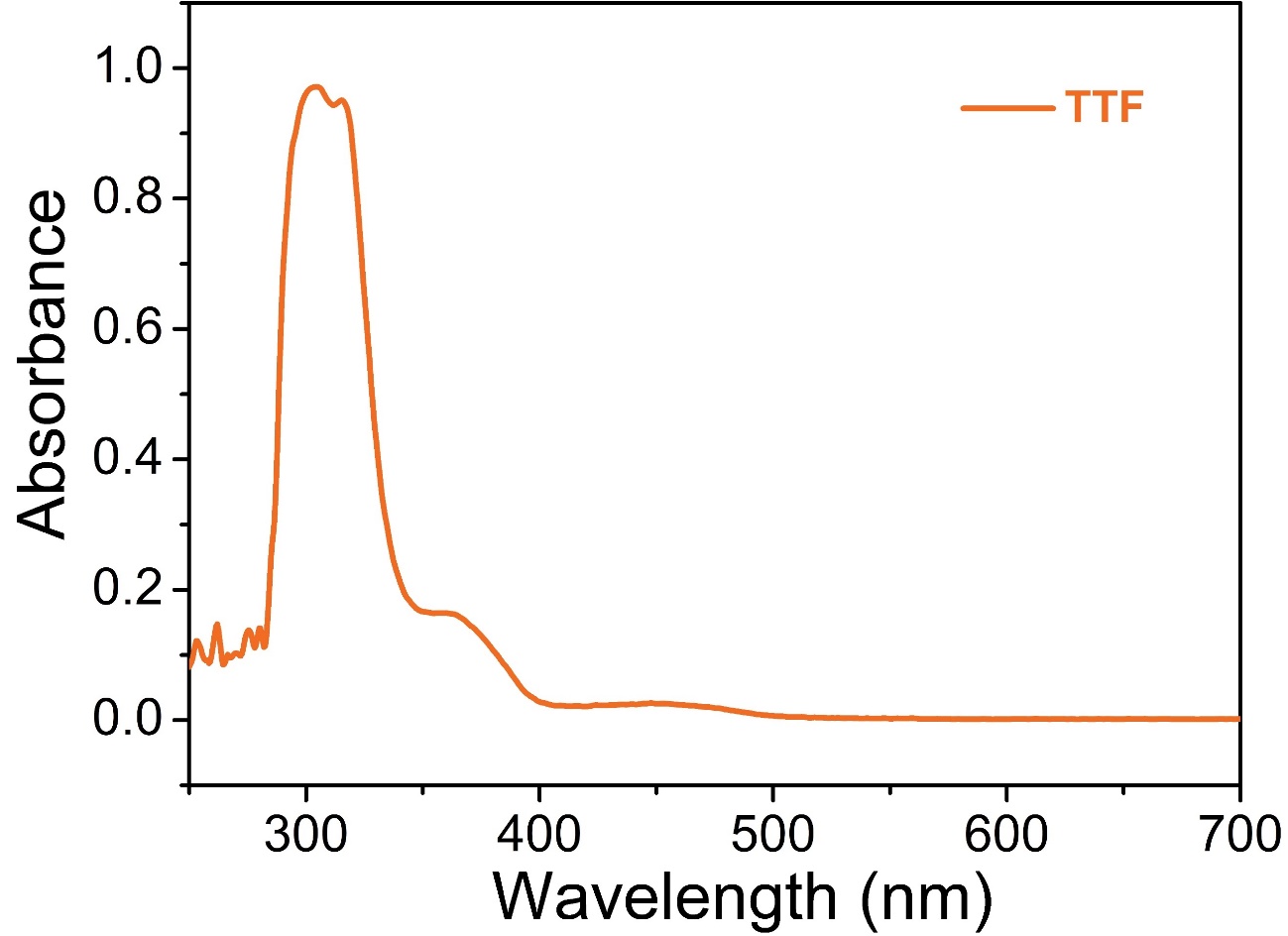


**Figure S4**. UV-vis spectra of TTF (100 μM) in CH_3_OH solution.


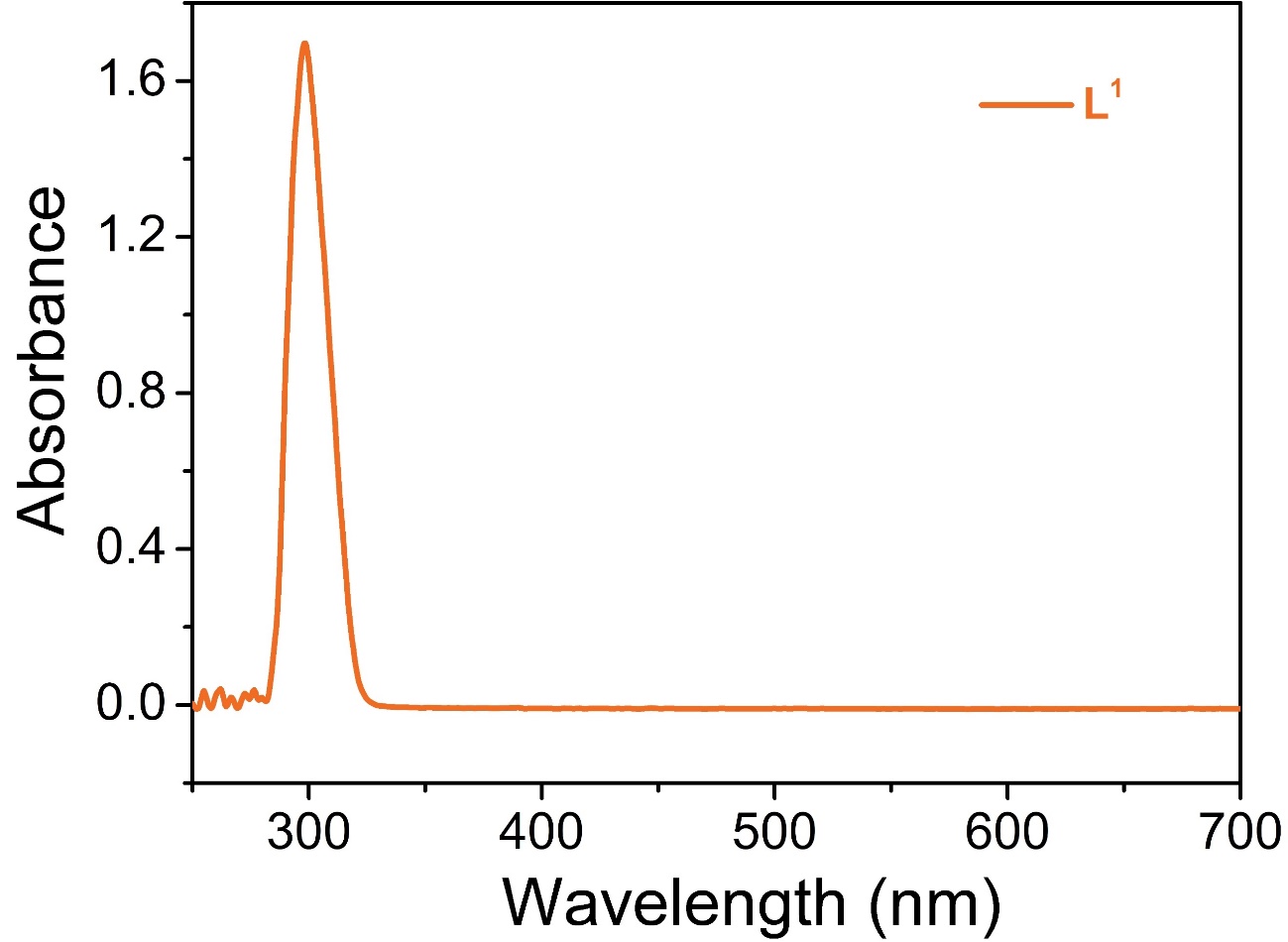


**Figure S5**. UV-vis spectra of L^1^ (100 μM) in CH_3_OH solution.


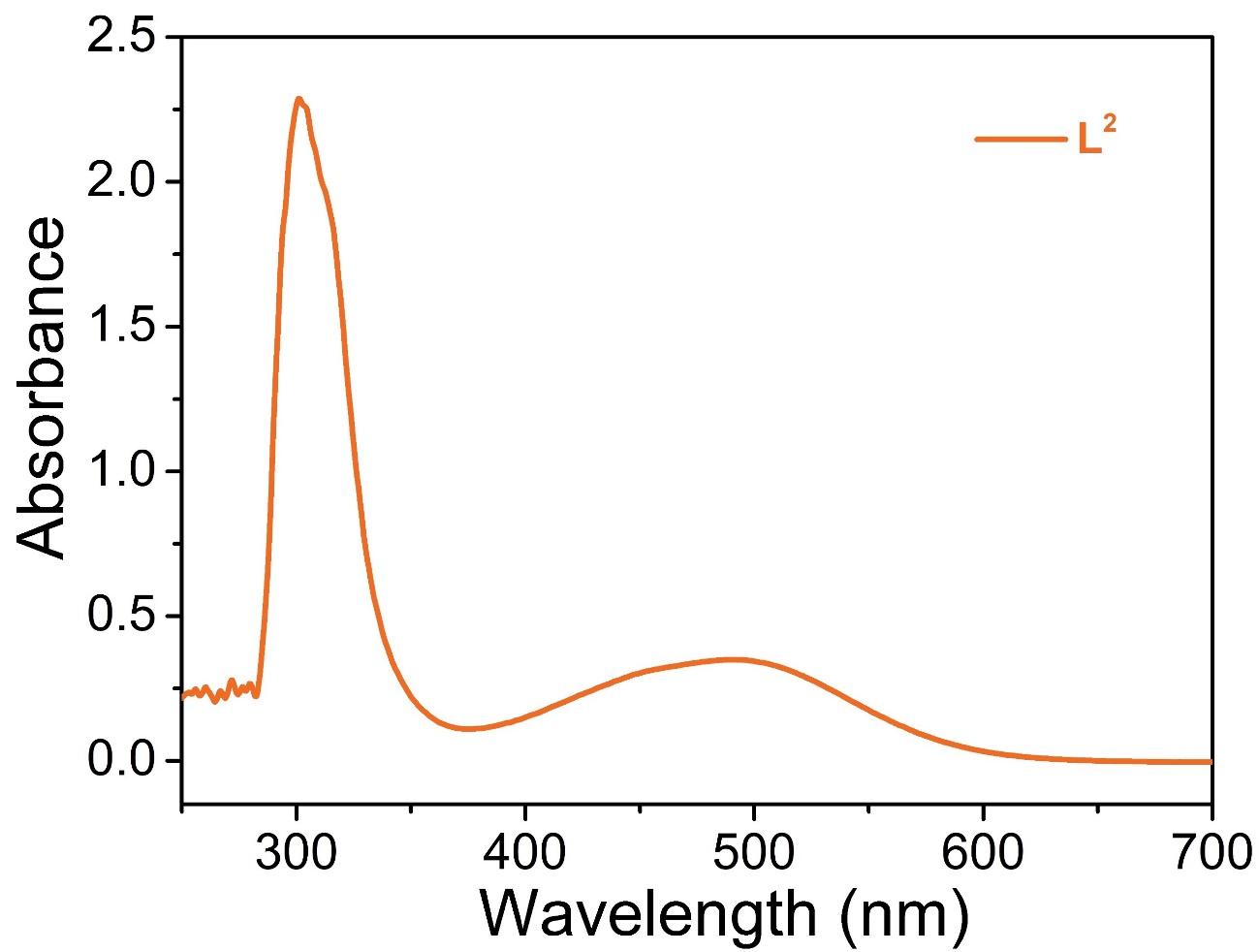


**Figure S6**. UV-vis spectra of L^2^ (100 μM) in CH_3_OH solution.


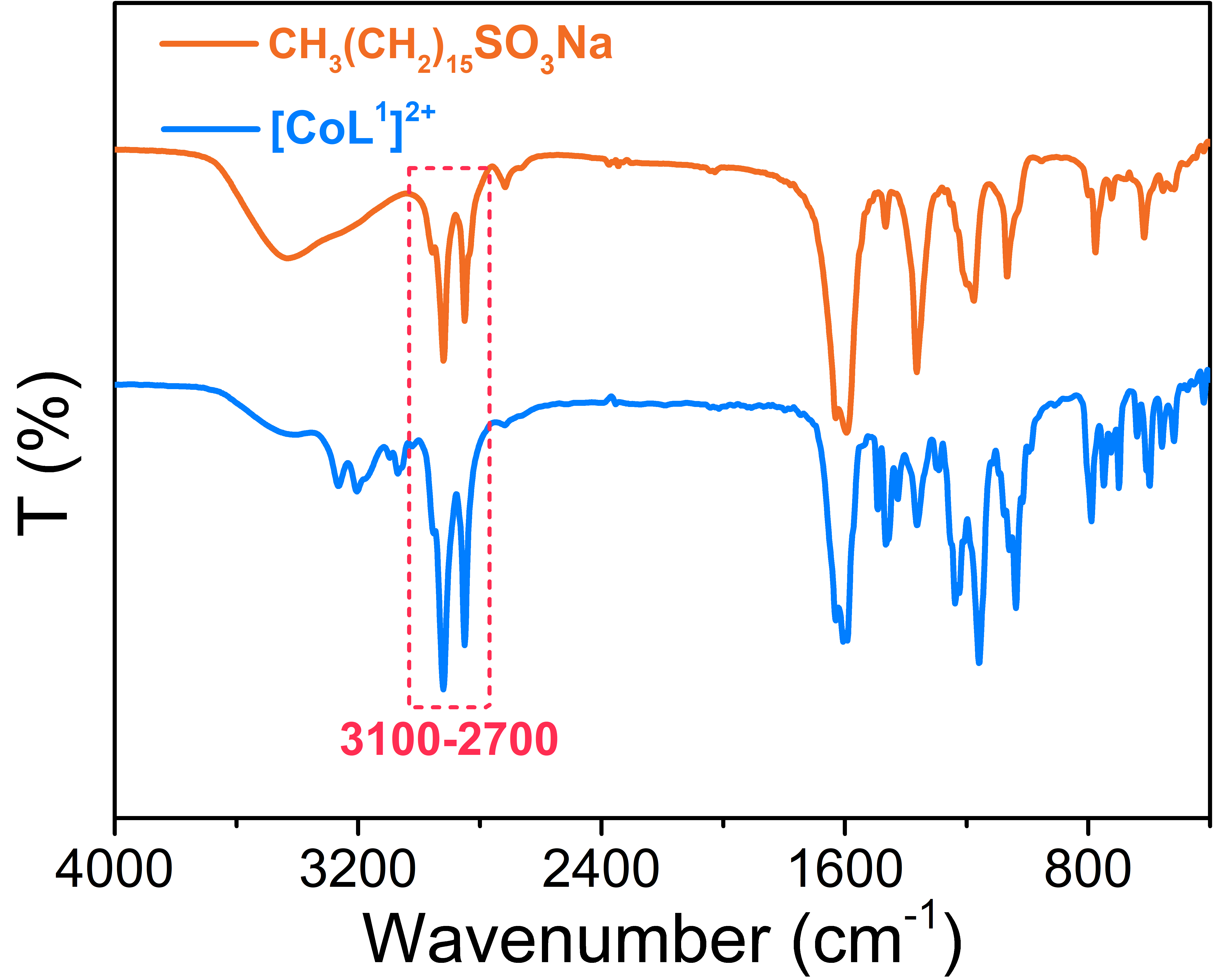


**Figure S7**. IR spectra of [CoL^1^]^2+^.


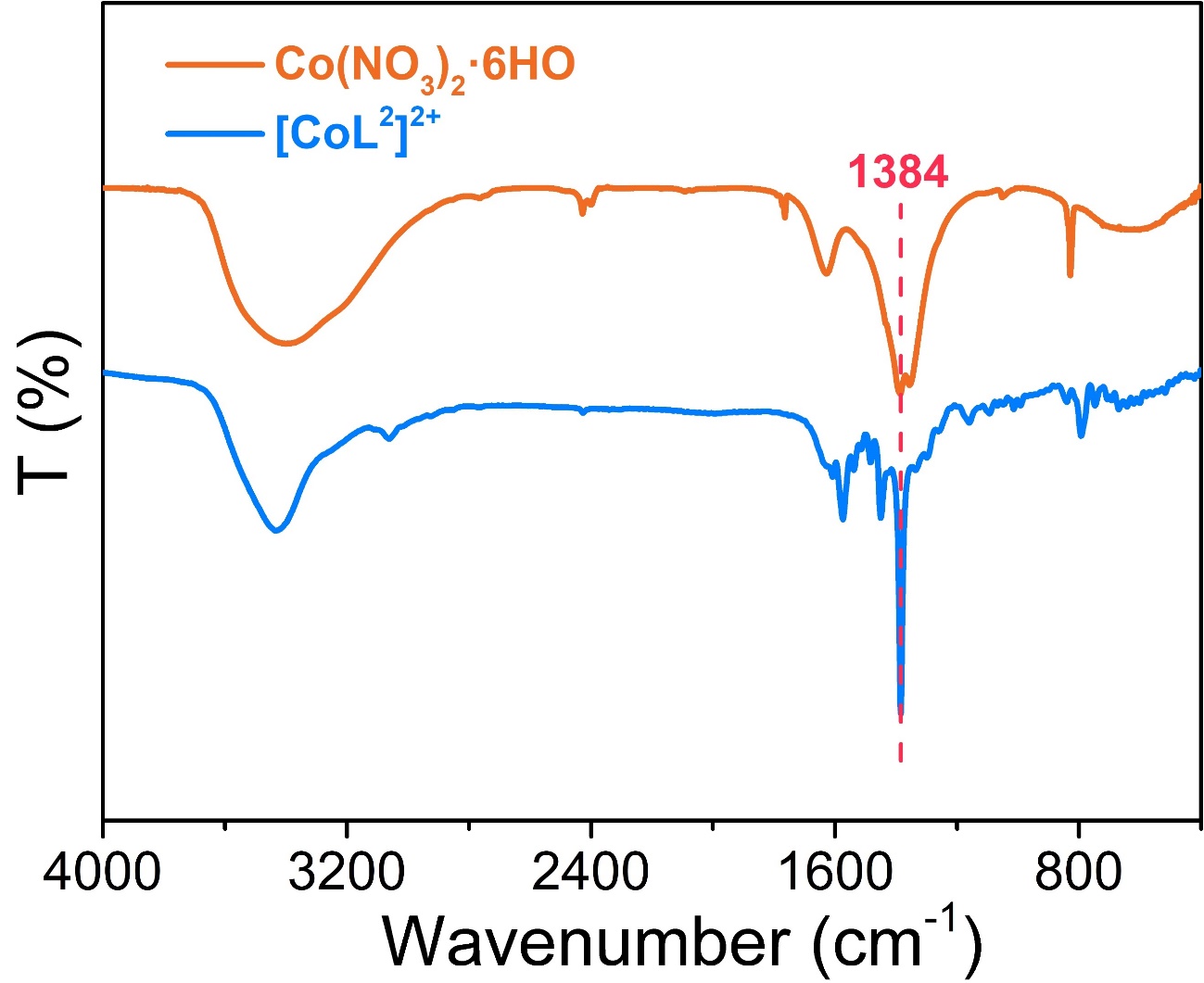


**Figure S8**. IR spectra of [CoL^2^]^2+^.


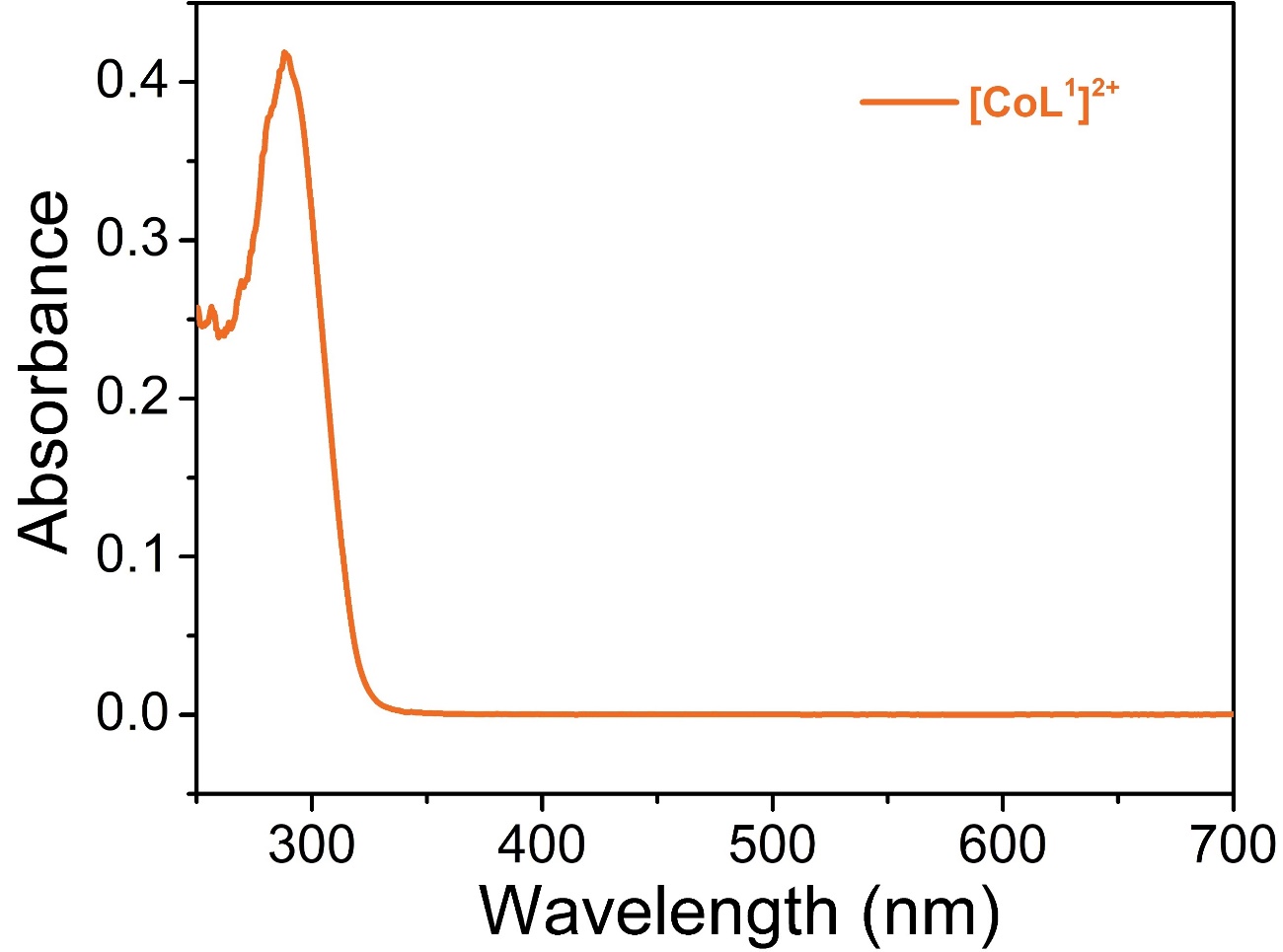


**Figure S9** UV-vis spectra of 10 μM [CoL^1^]^2+^ in CH_3_OH solution.


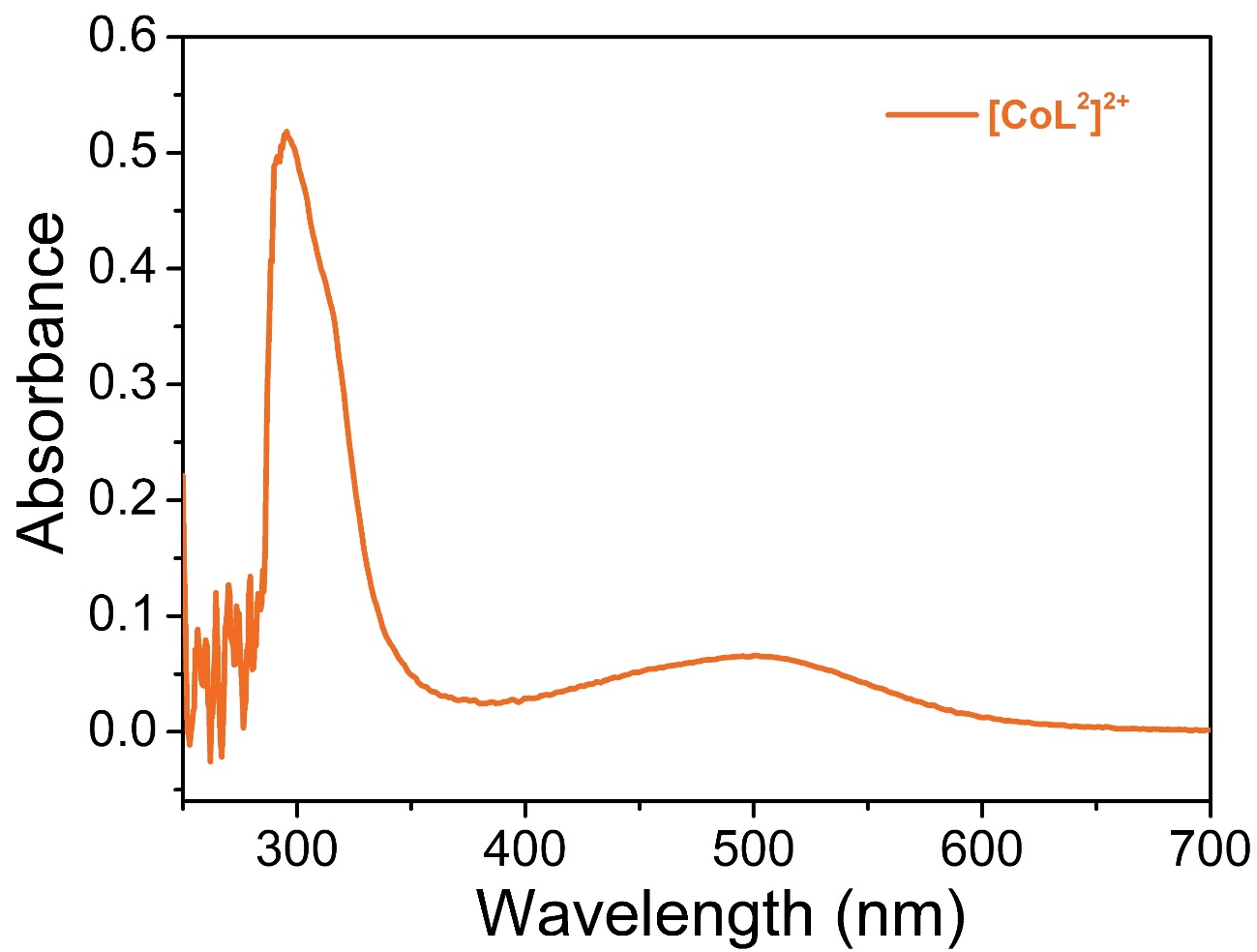


**Figure S10**. UV-vis spectra of 10 μM [CoL^2^]^2+^ in CH_3_OH solution.


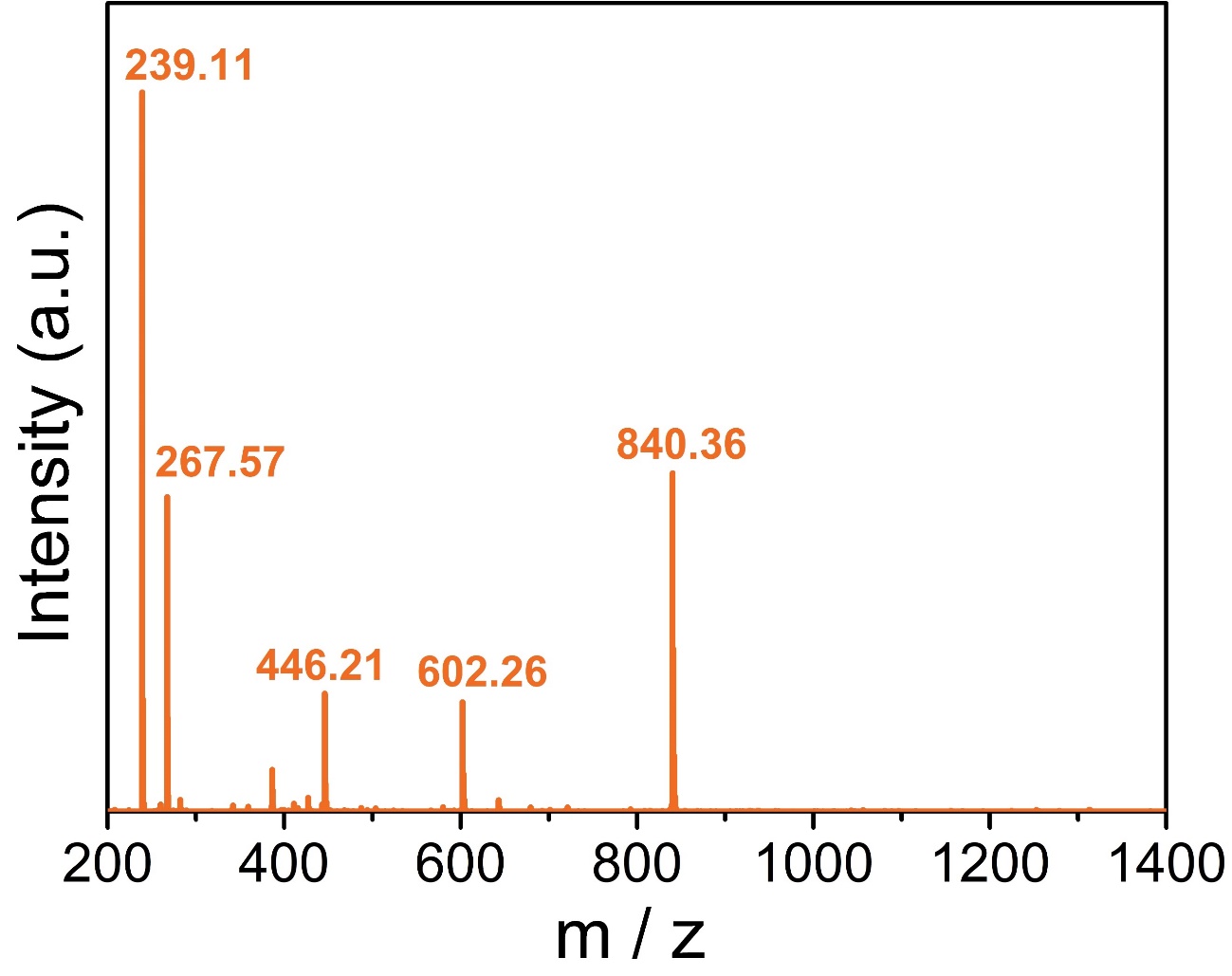


**Figure S11**. LC-MS of [CoL^1^]^2+^ in CH_3_OH. m/z = 239.11 [L^1^+H^+^]^+^; m/z = 267.57 [CoL^1^]^2+^; m/z = 446.21 [Co(H_33_C_16_SO_3_)+2CH_3_OH+H_2_O)]^+^; m/z = 602.26 [[CoL^1^]^2+^+CH_3_OH+H_2_O+OH^-^]^+^; m/z = 840.36 [[CoL^1^]^2+^+H_33_C_16_SO_3_^-^]^+^.


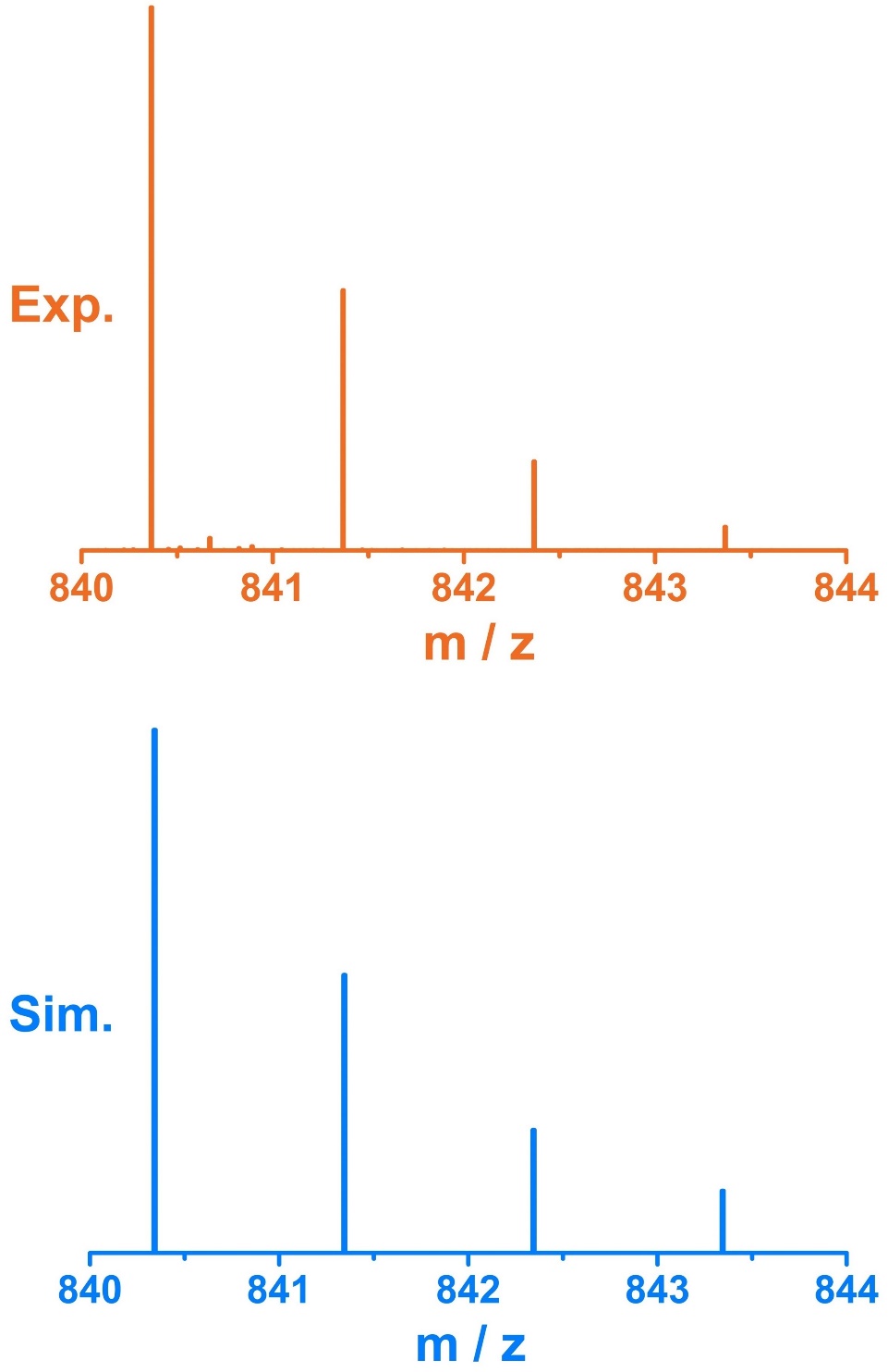


**Figure S12**. Experimental and simulated LC-MS for [CoL^1^]^2+^.


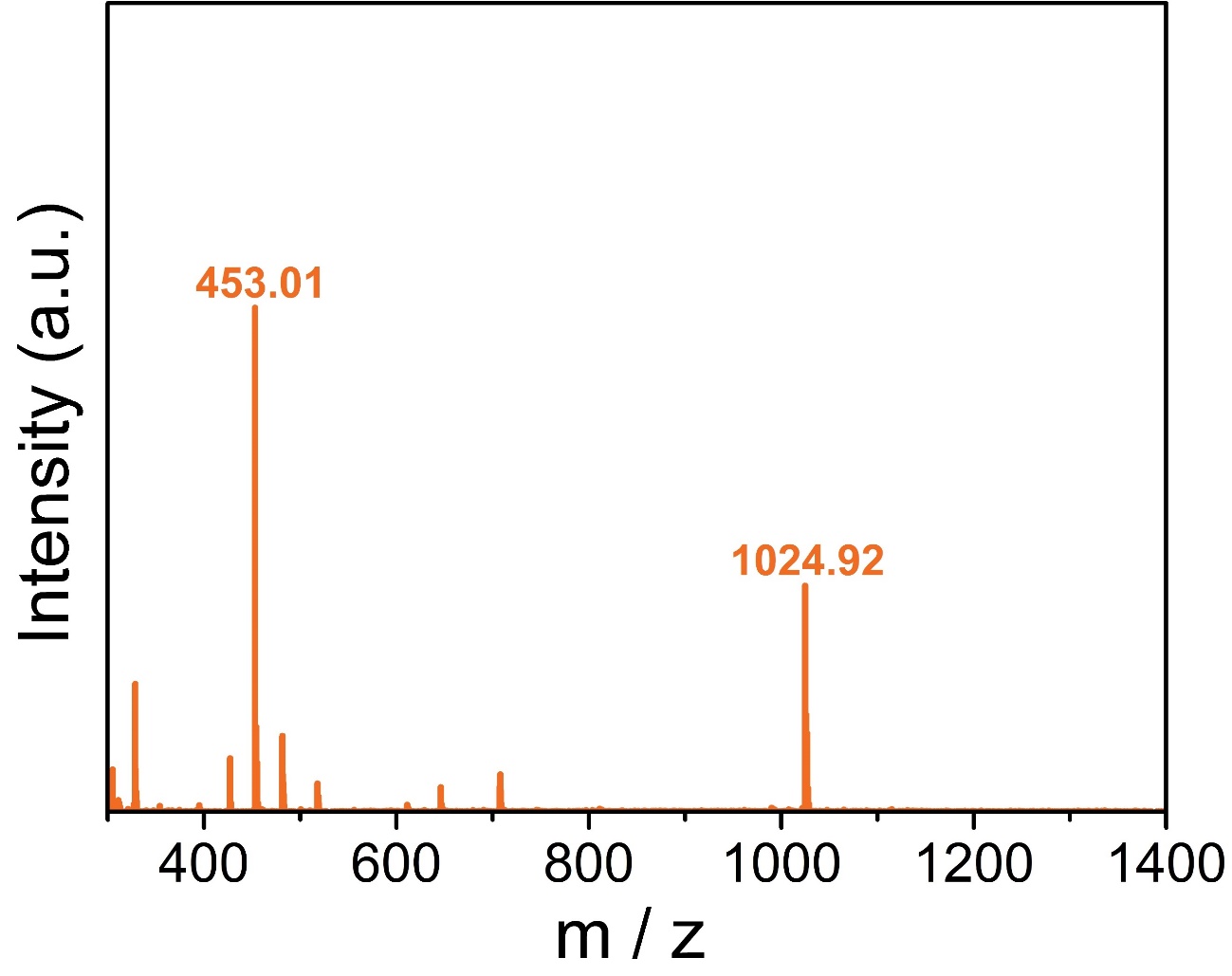


**Figure S13**. LC-MS of [CoL^2^]^2+^ in CH_3_OH. m/z = 453.01 [L^2^+H^+^]^+^; m/z = 1024.92 [[CoL^2^]^2+^+NO_3_^-^]^+^.


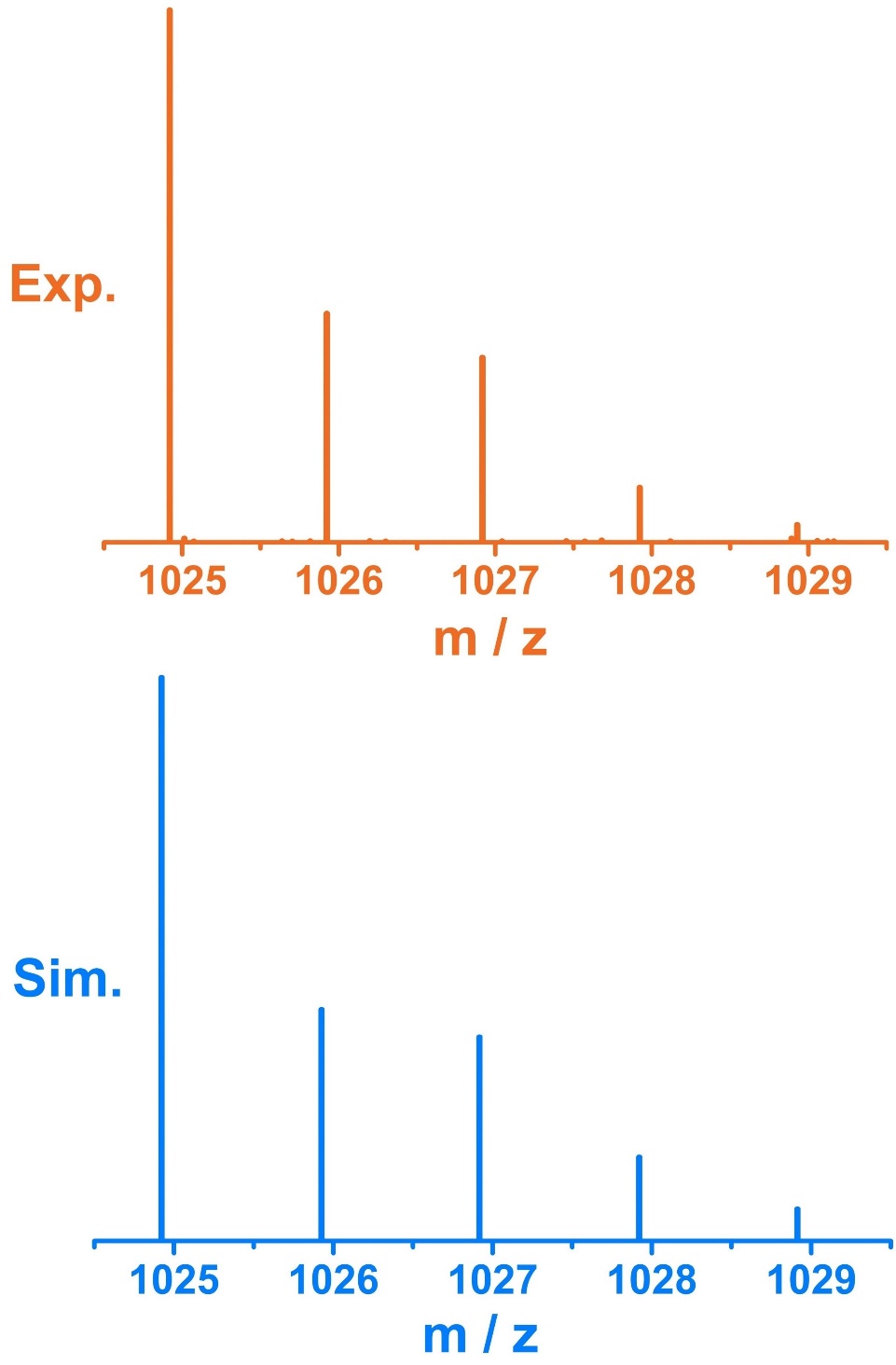


**Figure S14**. Experimental and simulated LC-MS for [CoL^2^]^2+^.


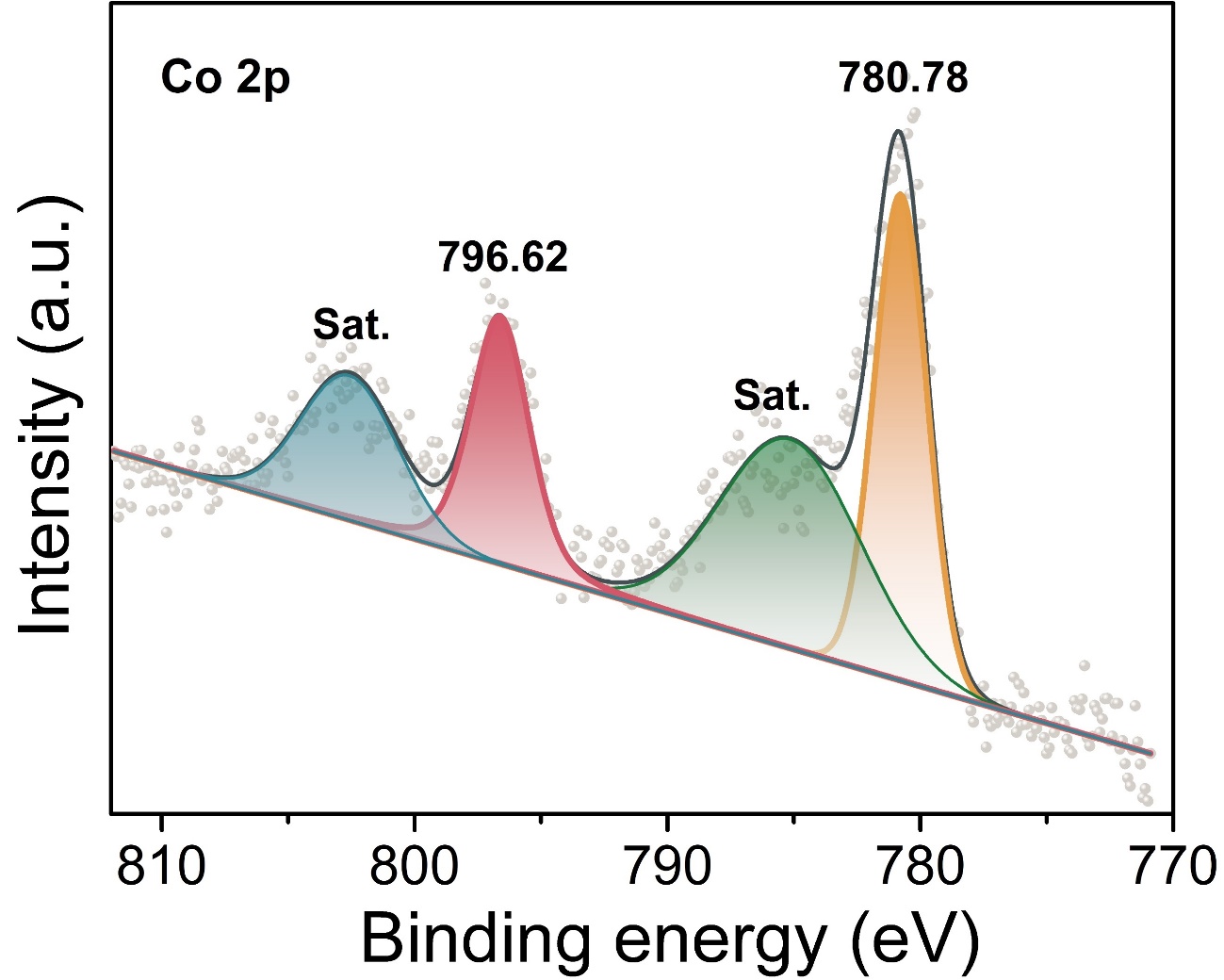


**Figure S15**. Co 2p XPS spectra of [CoL^2^]^2+^.


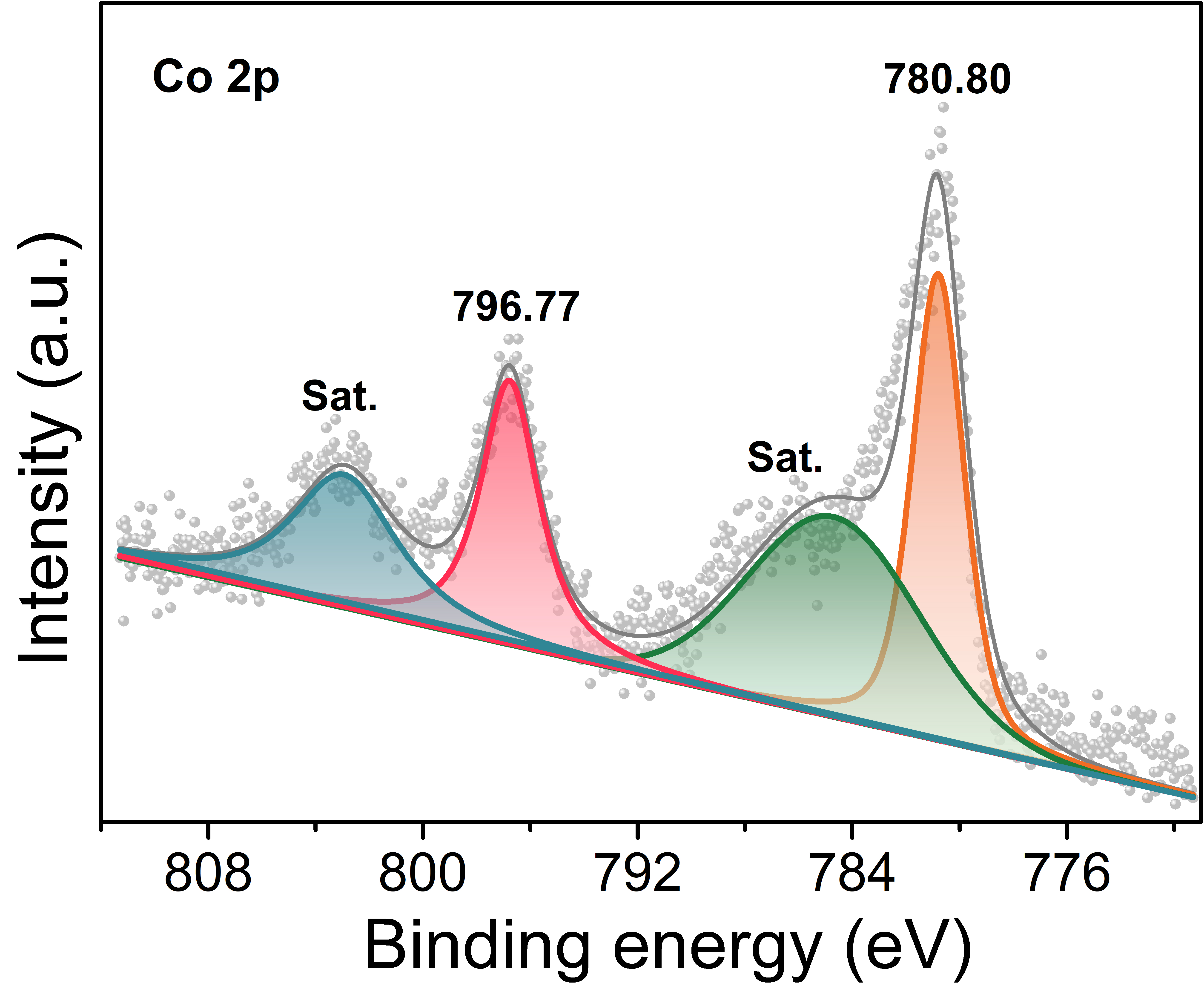


**Figure S16**. Co 2p XPS spectrum of [CoL^1^]^2+^.


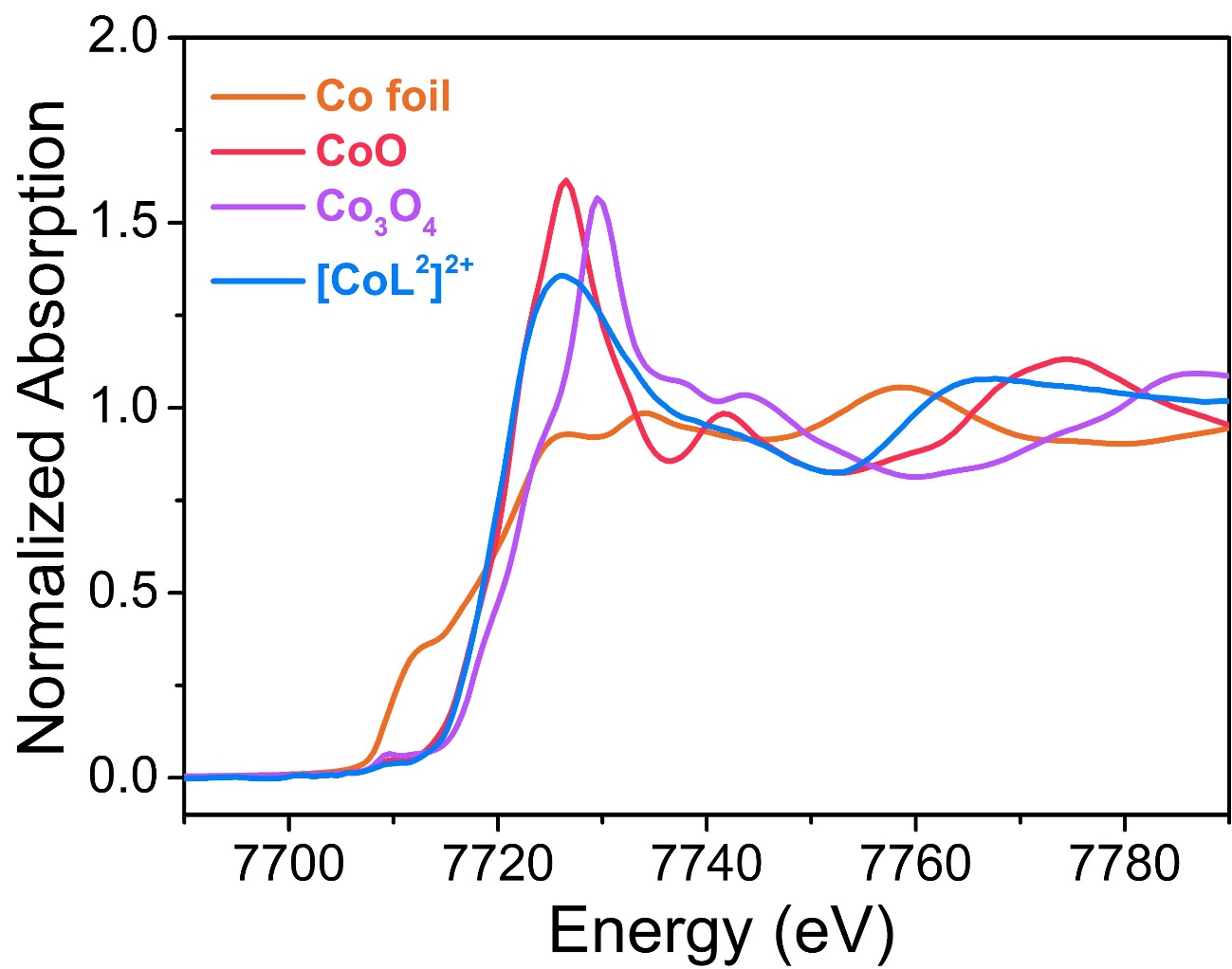


**Figure S17**. Co K-edge XANES spectra of [CoL^2^]^2+^, Co foil, CoO, and Co_3_O_4_.


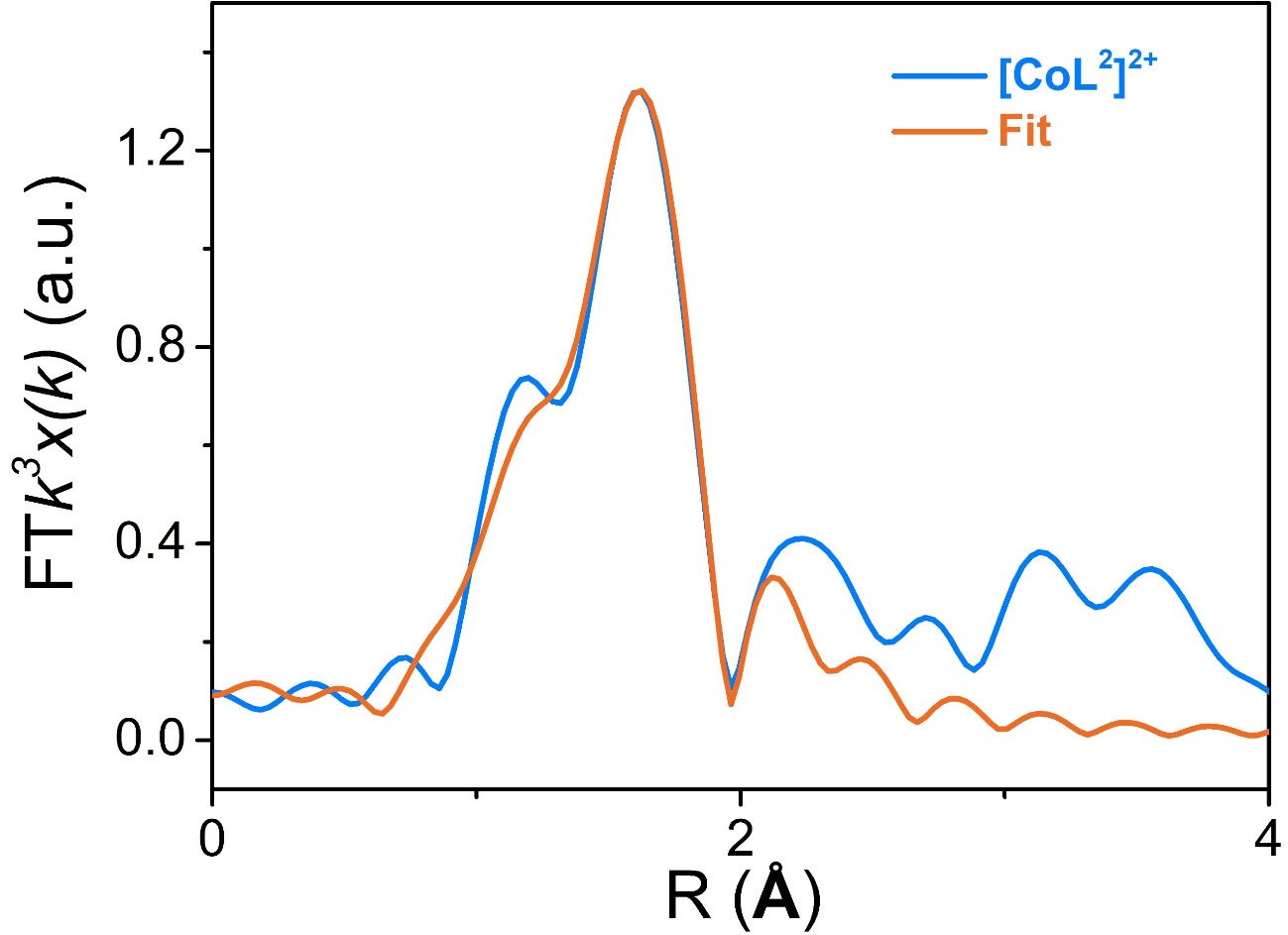


**Figure S18**. Corresponding FT-EXAFS R space fitting result for [CoL^2^]^2+^.


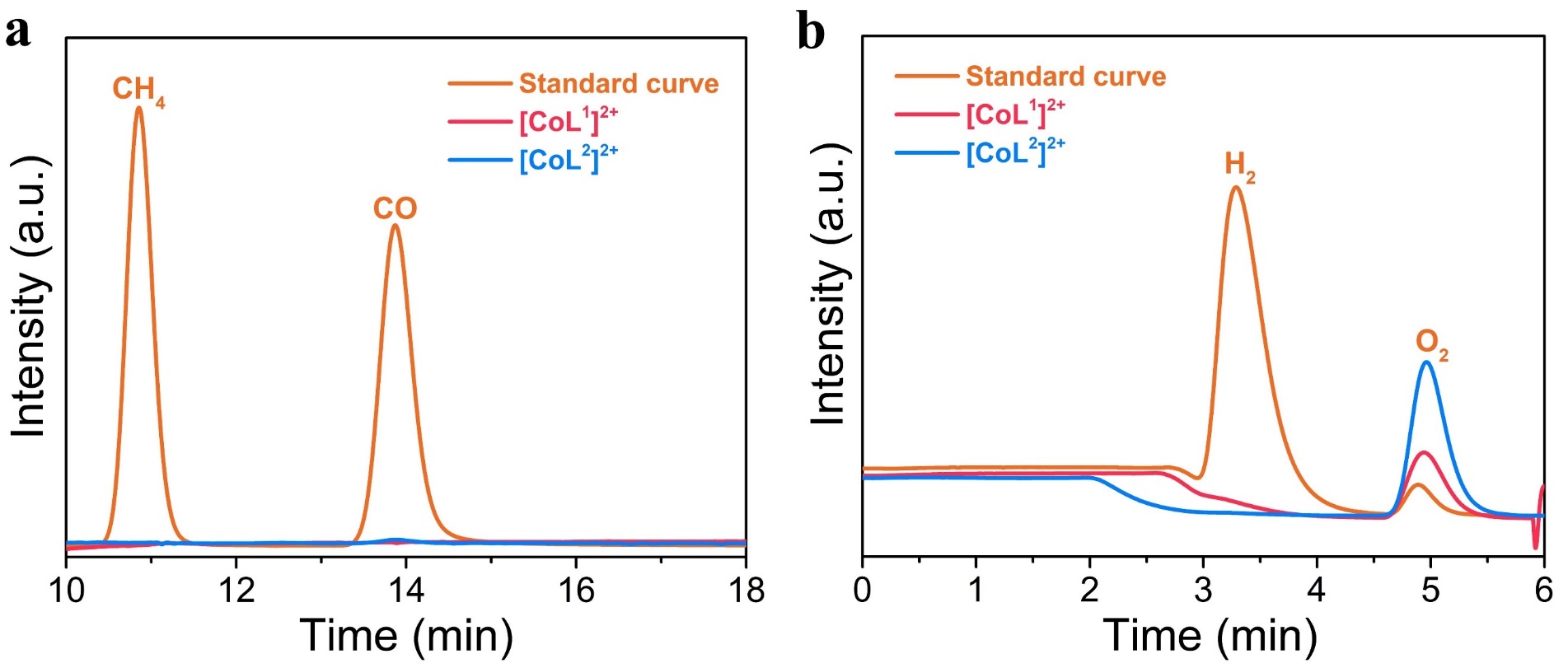


**Figure S19**. Gas chromatograms of products generated from photocatalytic CO_2_ reduction using [CoL^1^]^2+^ and [CoL^2^]^2+^ as the photocatalyst, (a) TCD detector and (b) FID detector. Reaction conditions: photocatalysts (1 μM), 5 mL CO_2_-saturated CH_3_OH/H_2_O (*v/v* = 4:1) solution, 300 W Xe lamp, 14 h, 25 ^o^C.


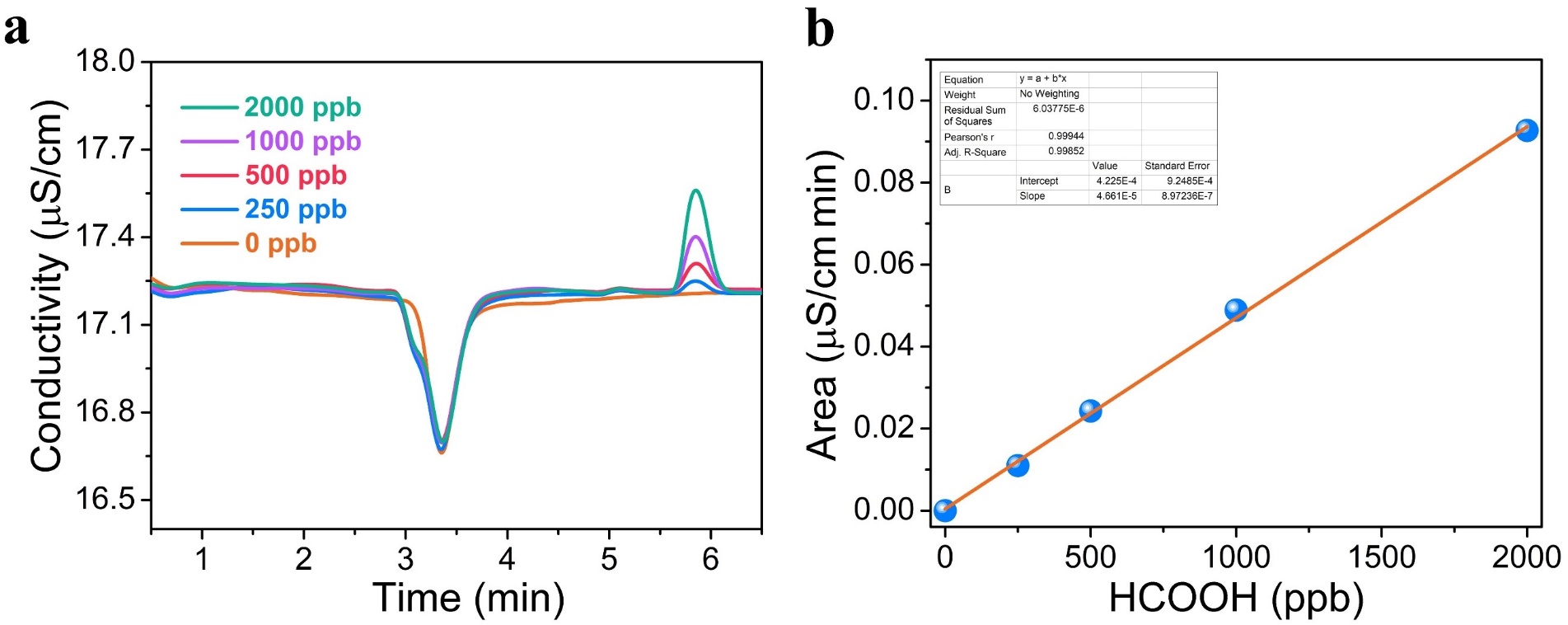


**Figure S20**. (a) Ion chromatography (IC) spectra of HCOOH at different concentrations and (b) IC standard curves for HCOOH.


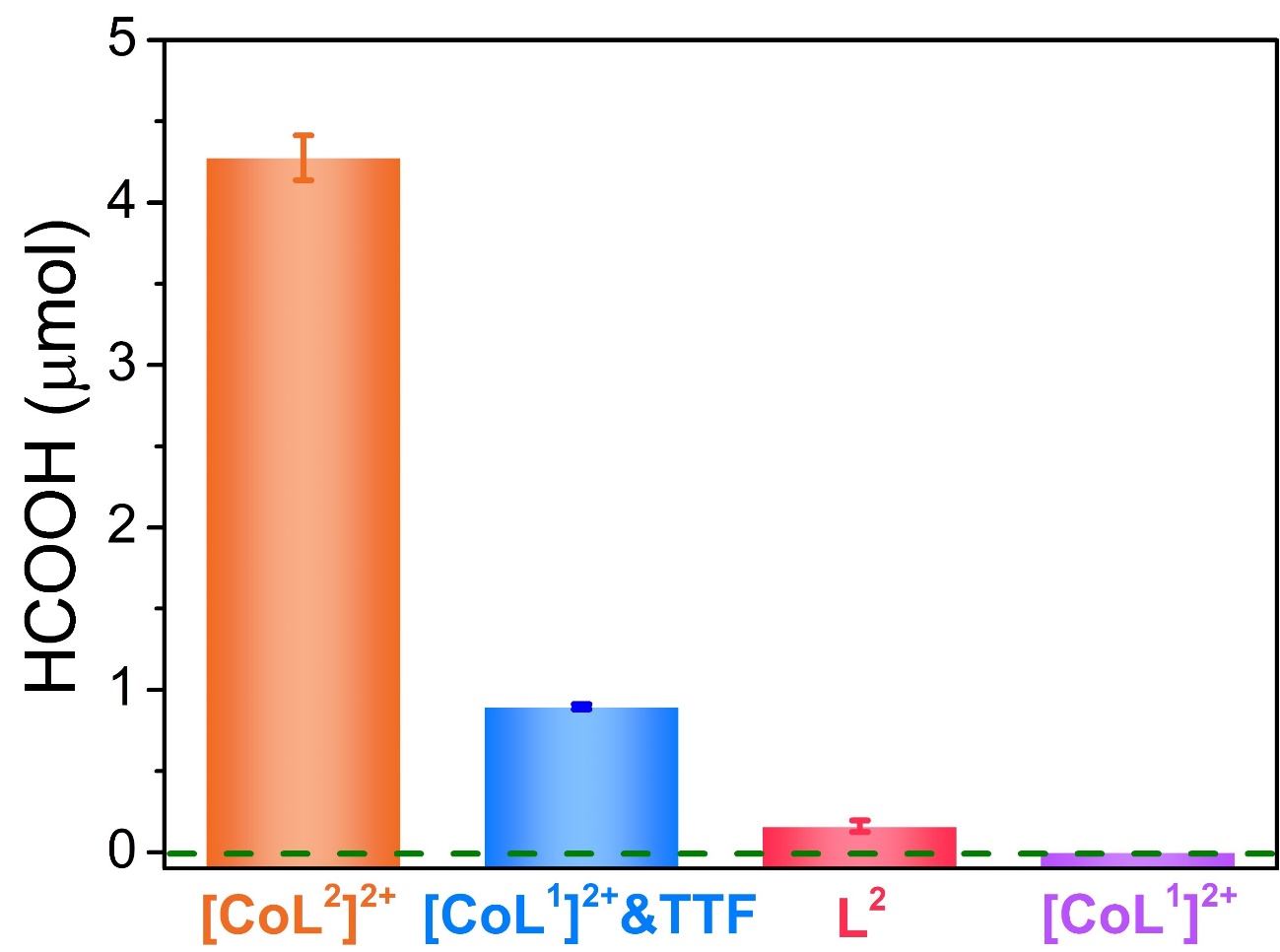


**Figure S21**. Photocatalytic activity of [CoL^2^]^2+^, [CoL^1^]^2+^&TTF, L^2^, and [CoL^1^]^2+^ for CO_2_ reduction and CH_3_OH oxidation. Reaction conditions: [CoL^2^]^2+^ (1 μM) or [[CoL^1^]^2+^ (1 μM)+TTF (2 μM)] or L^2^ (2 μM) or [CoL^1^]^2+^ (1 μM), 5 mL CO_2_-saturated CH_3_OH/H_2_O (*v/v* = 4:1) solution, 300 W Xe lamp (320 < *λ* < 780 nm; light intensity: 200 mW cm^-2^), 14 h, 25 ^o^C (The error bars are standard deviations calculated from the results of three parallel experiments).


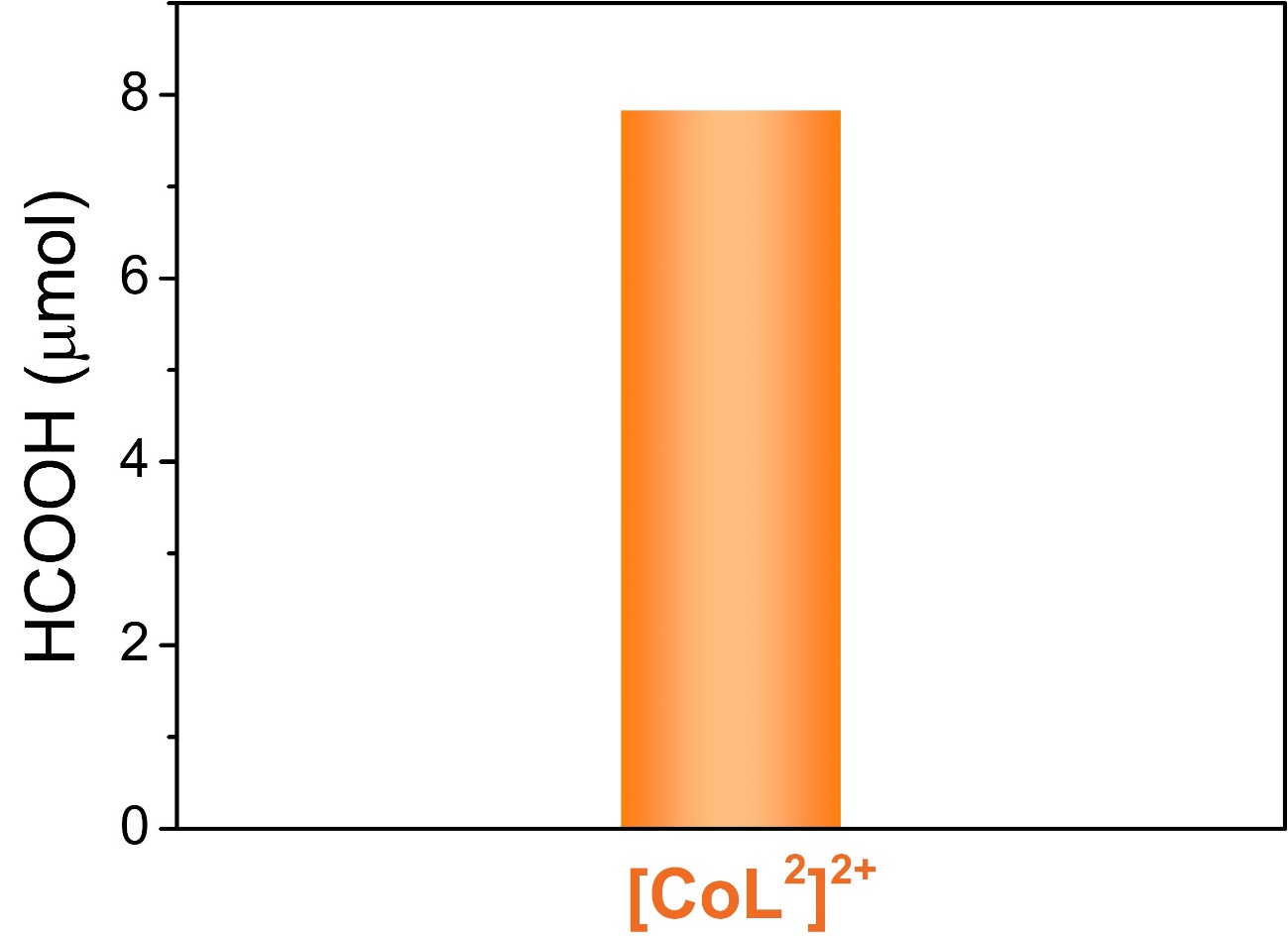


**Figure S22**. TON of HCOOH in photocatalytic CO_2_ reduction coupled with CH_3_OH oxidation by [CoL^2^]^2+^ under an LED light (λ = 365 nm) for 10 h. Conditions: [CoL^2^]^2+^ (1 μM), 5 mL CO_2_-saturated CH_3_OH/H_2_O (*v/v* = 4:1) solution, LED lamp (λ = 365 nm), 10 h, 25 ^o^C.


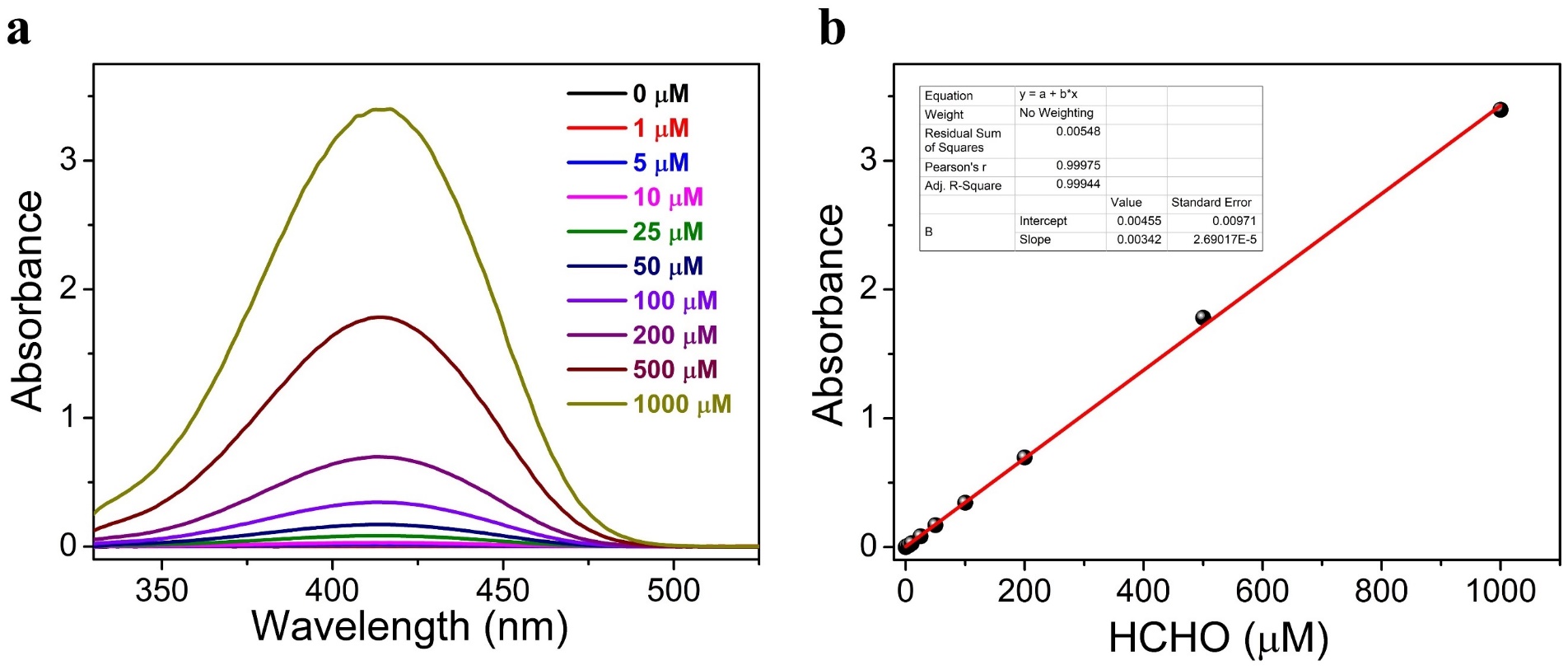


**Figure S23**. (a) UV-vis absorption spectra of HCHO at different concentrations by colorimetric method and (b) standard curves for HCHO.


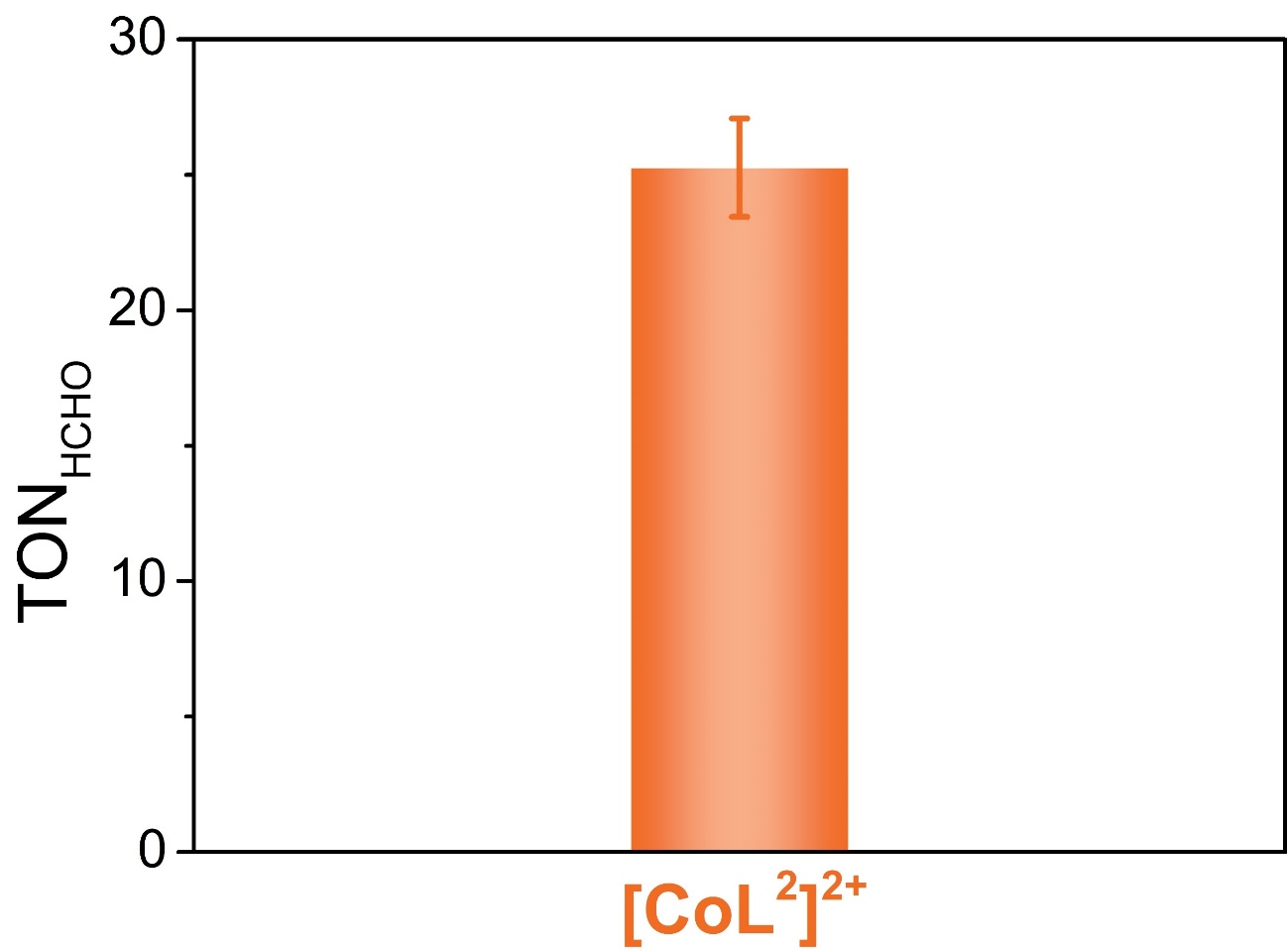


**Figure S24**. TON of HCHO in photocatalytic CO_2_ reduction coupled with CH_3_OH oxidation by [CoL^2^]^2+^. Conditions: [CoL^2^]^2+^ (1 μM), 5 mL CO_2_-saturated CH_3_OH/H_2_O (*v/v* = 4:1) solution, 300 W Xe lamp (320 < *λ* < 780 nm; light intensity: 200 mW cm^-2^), 14 h, 25 ^o^C (The error bars are standard deviations calculated from the results of three parallel experiments).


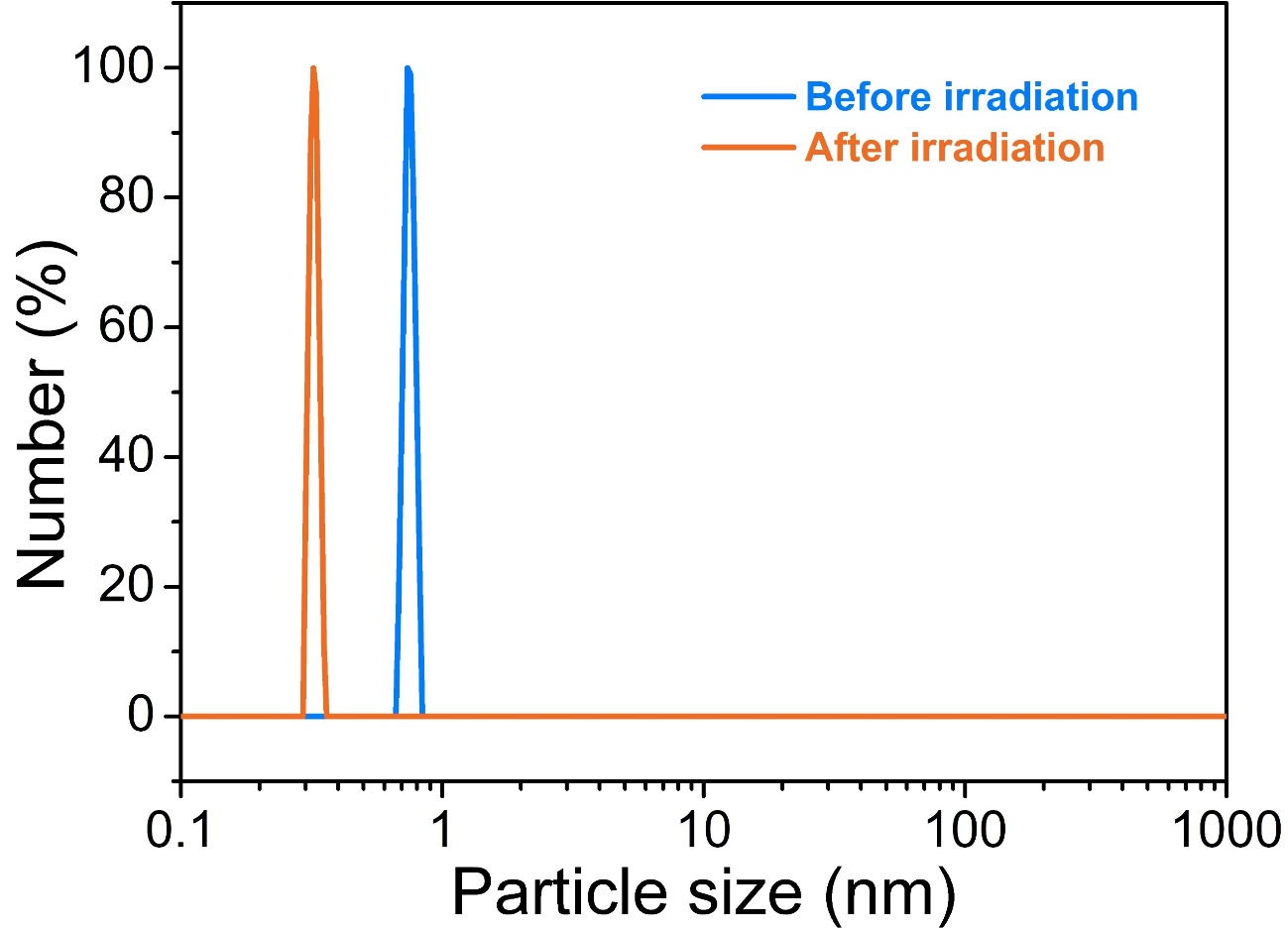


**Figure S25**. Particle size distribution of a CH_3_OH/H_2_O (*v/v* = 4:1) solution containing [CoL^2^]^2+^ (10 μM) before and after irradiation for 14 h, obtained from dynamic light scattering (DLS) measurements.


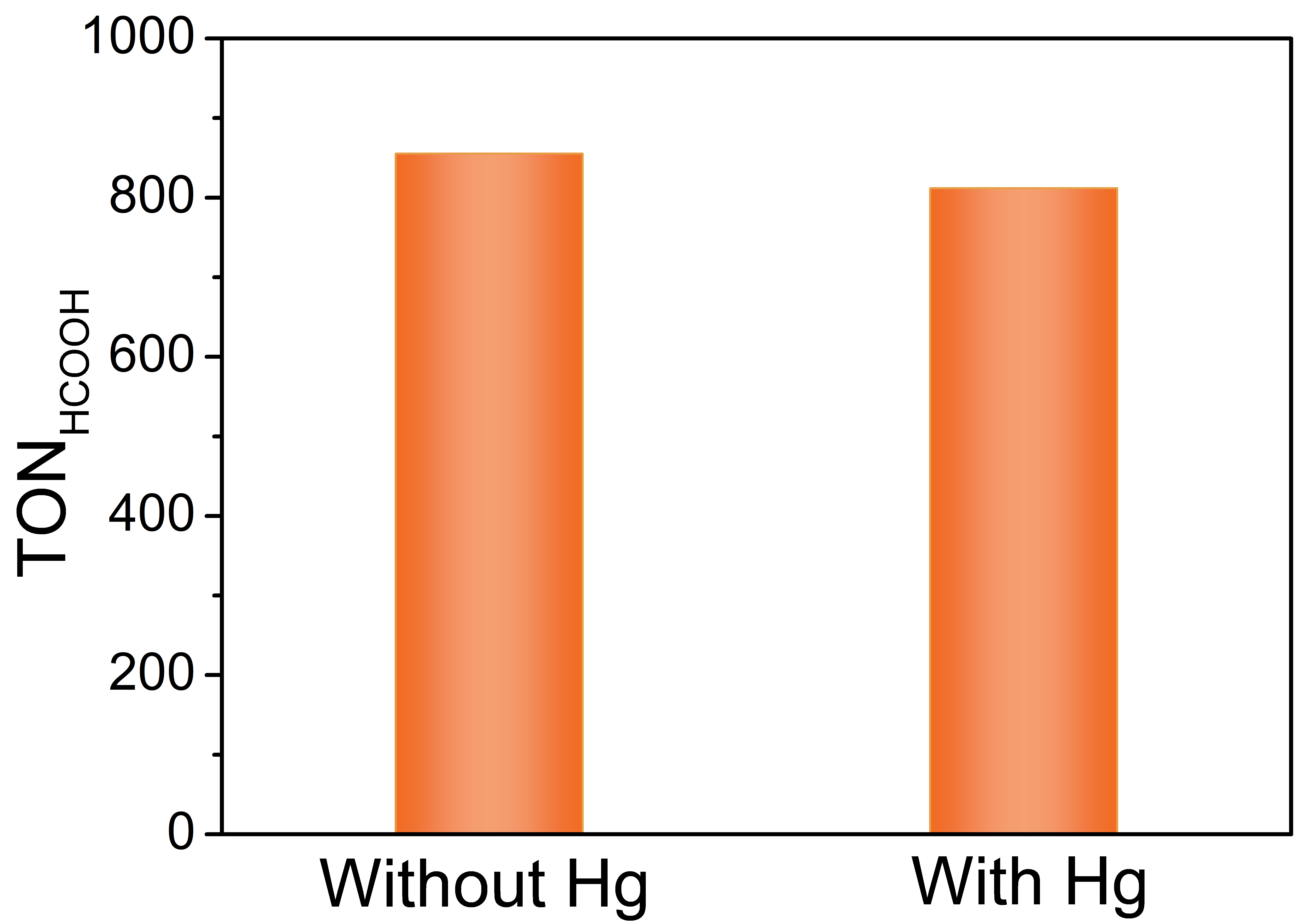


**Figure S26**. Comparison of the photocatalytic CO_2_ reduction coupled with CH_3_OH oxidation performance by [CoL^2^]^2+^ with and without 0.5 mL Hg(0) in a CH_3_OH/H_2_O (*v/v* = 4:1) solution.


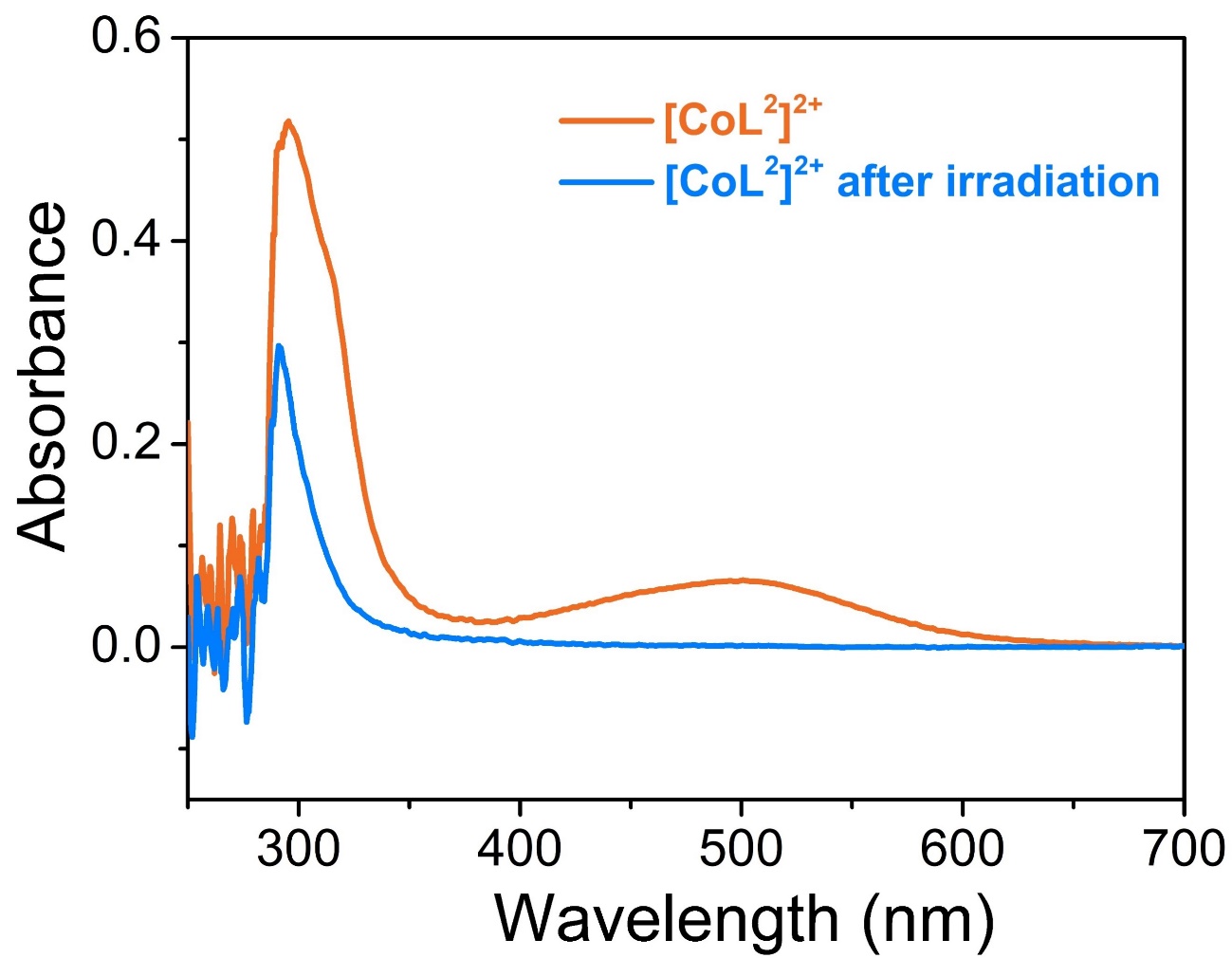


**Figure S27**. UV-vis absorption spectra of [CoL^2^]^2+^ (10 μM) before and after illumination for 14 h in CH_3_OH/H_2_O (*v*/*v* = 4:1) solution.


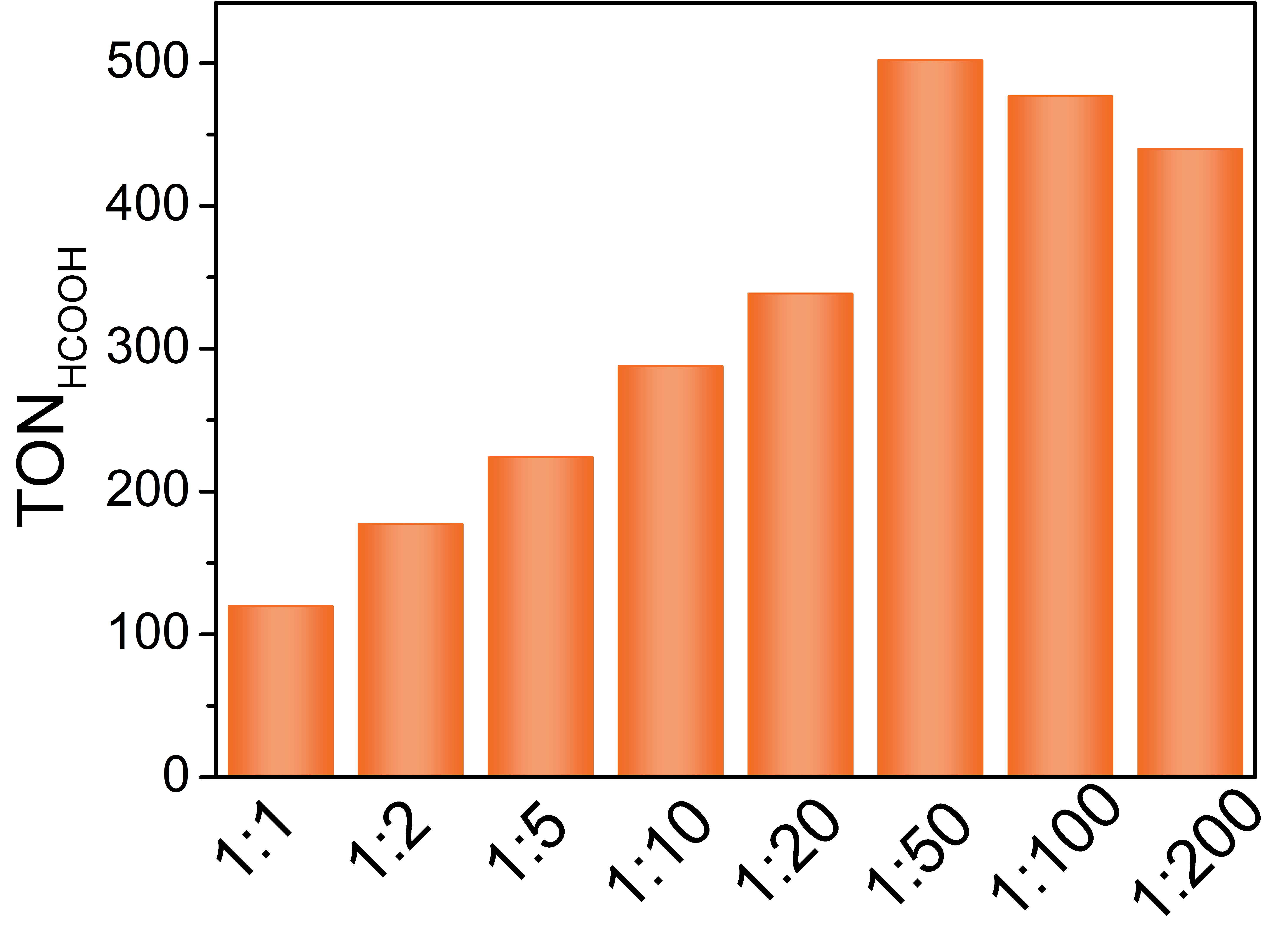


**Figure S28**. Photocatalytic performance of [CoL^1^]^2+^&TTF with different molar ratio of [CoL^1^]^2+^ to TTF (*n*:*n*) in CO_2_ reduction coupled with CH_3_OH oxidation.


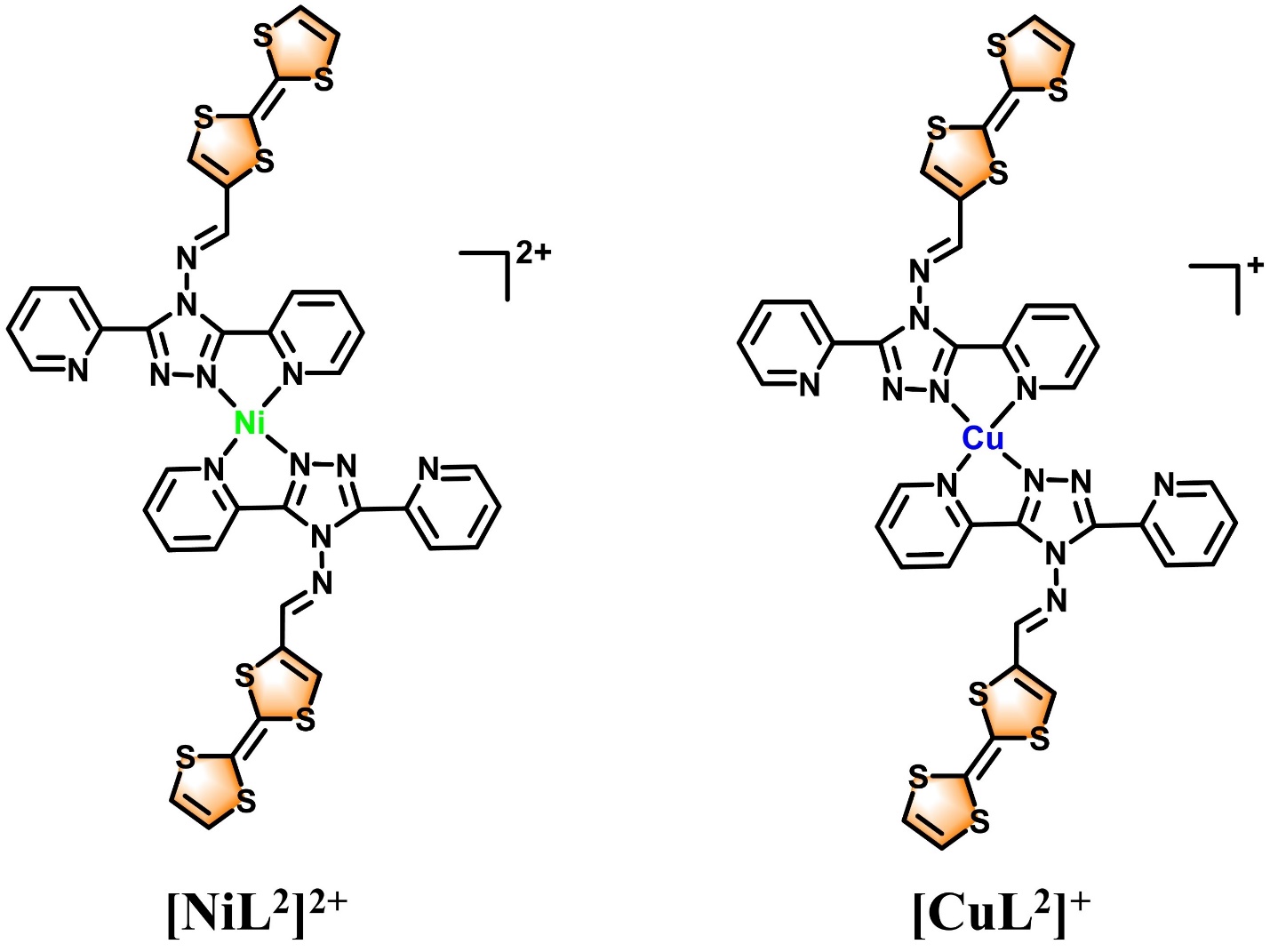


**Figure S29**. Chemical structures of [NiL^2^]^2+^ and [CuL^2^]^+^.


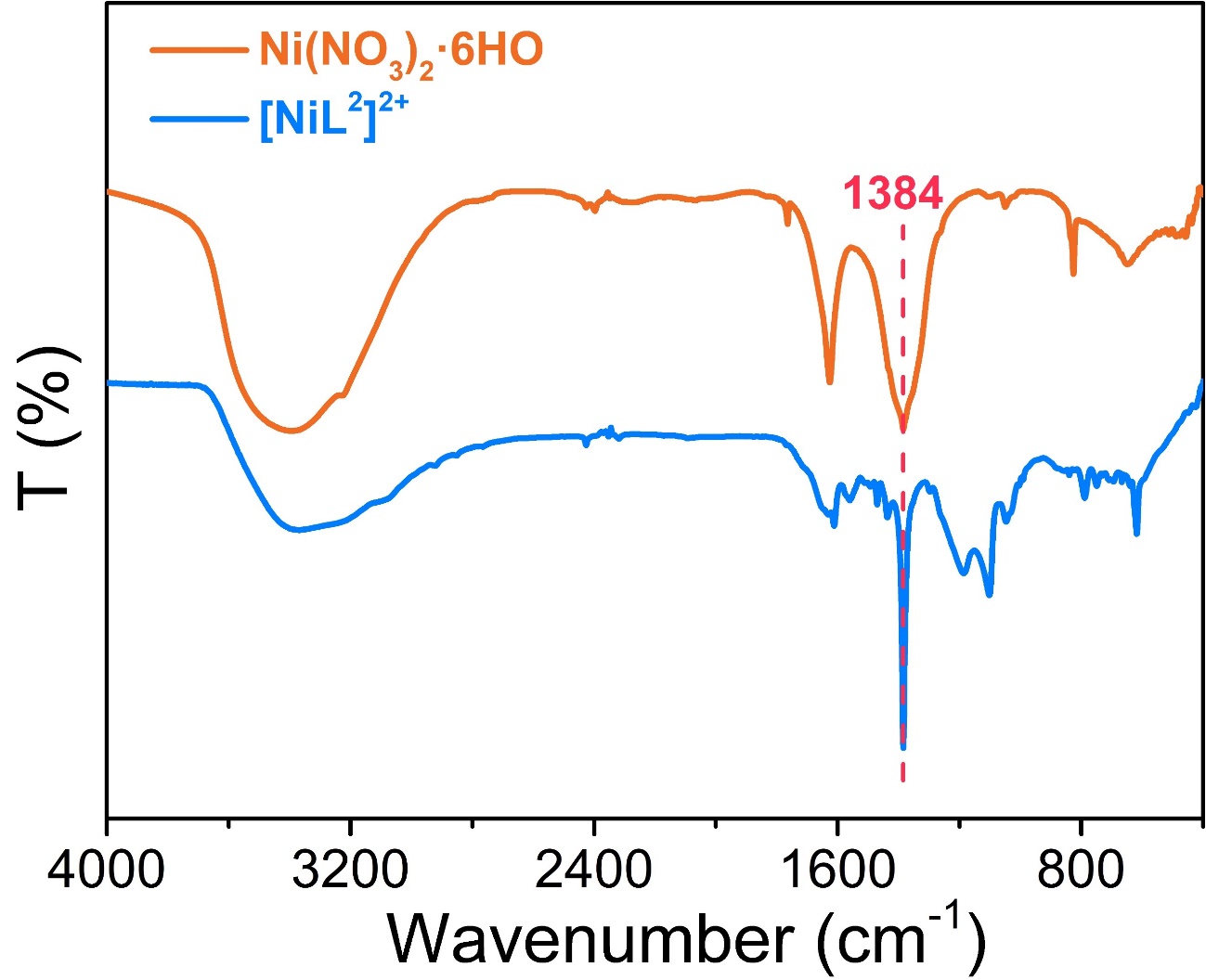


**Figure S30**. IR spectra of [NiL^2^]^2+^.


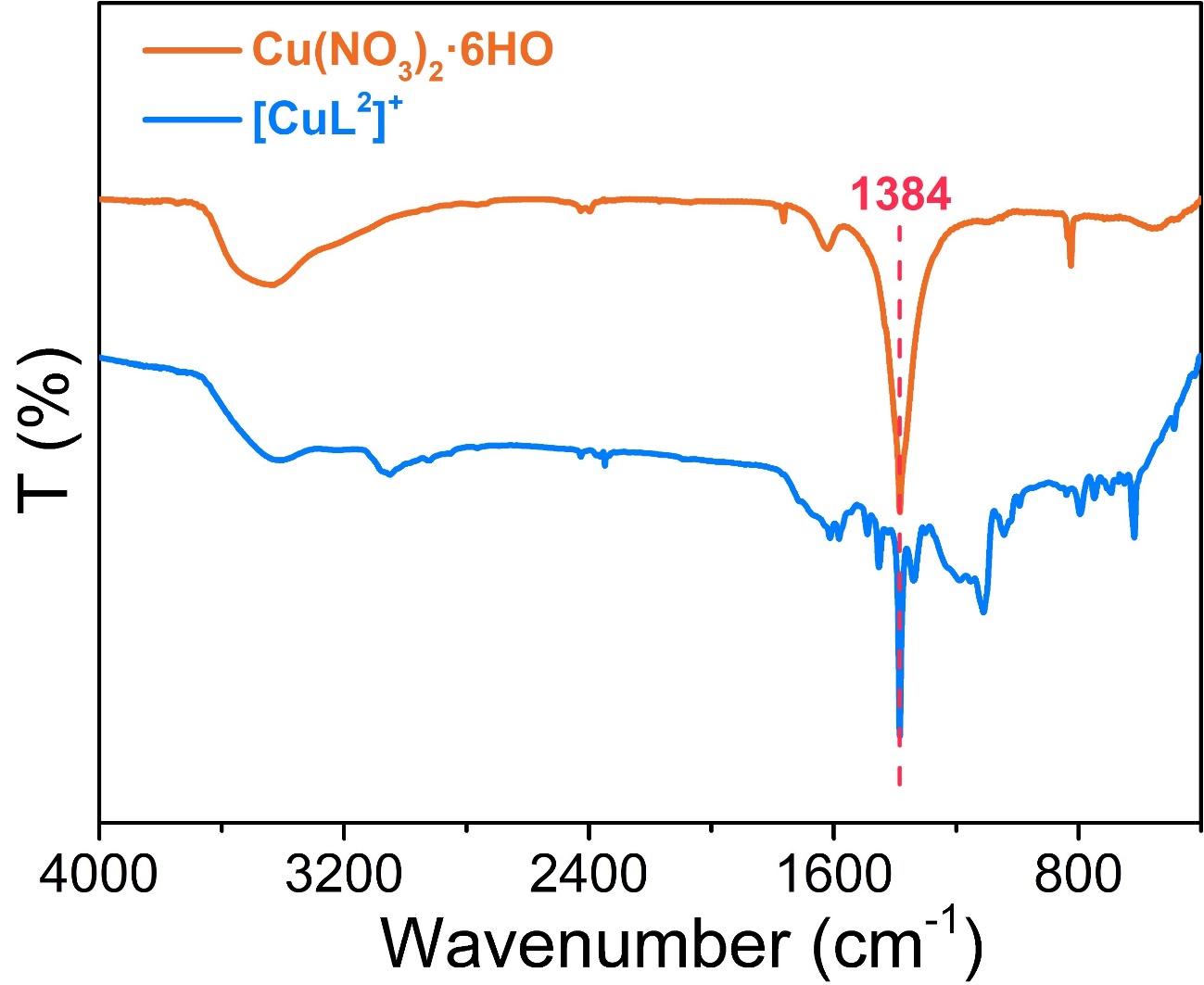


**Figure S31**. IR spectra of [CuL^2^]^+^.


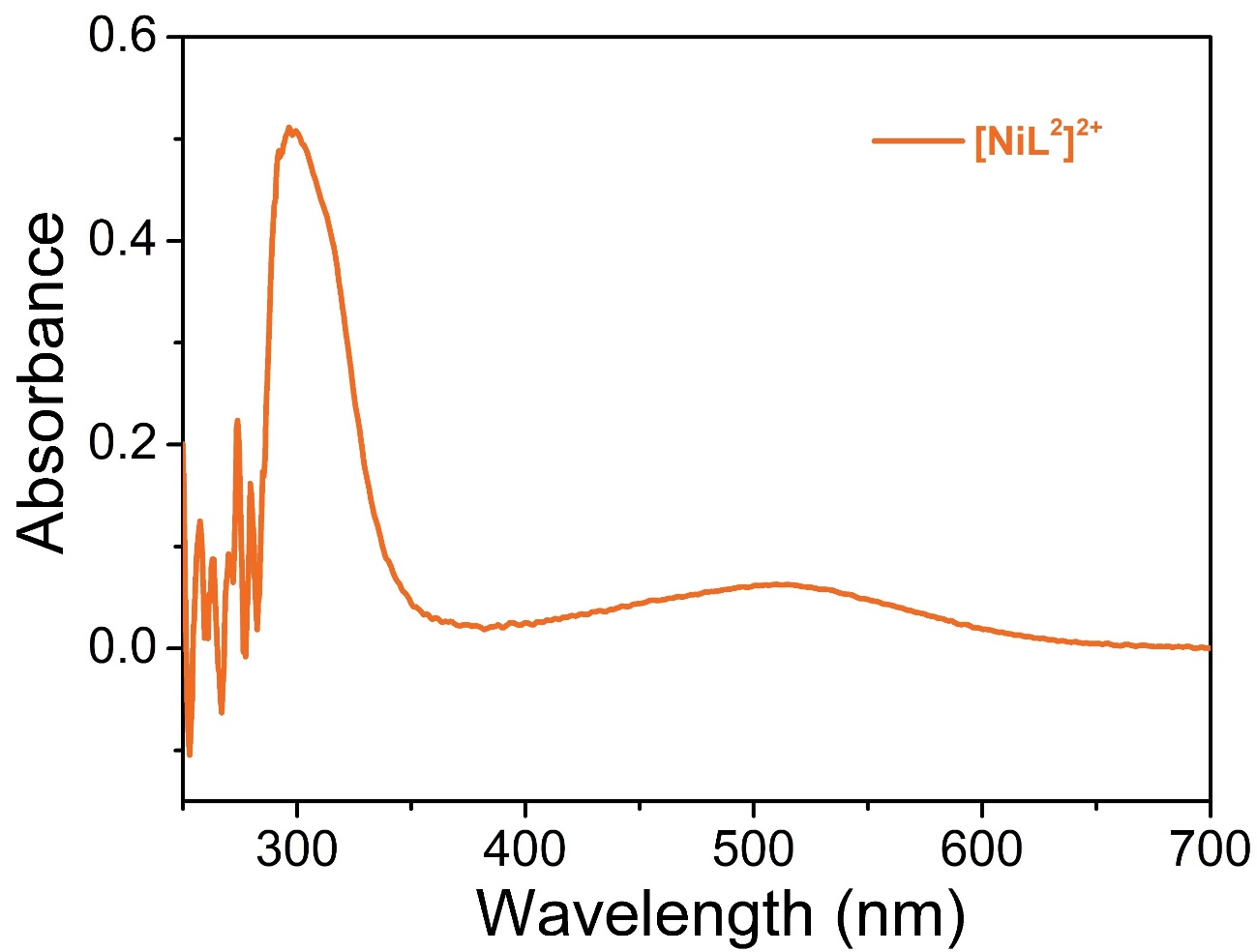


**Figure S32**. UV-vis spectra of 10 μM [NiL^2^]^2+^ in CH_3_OH solution.


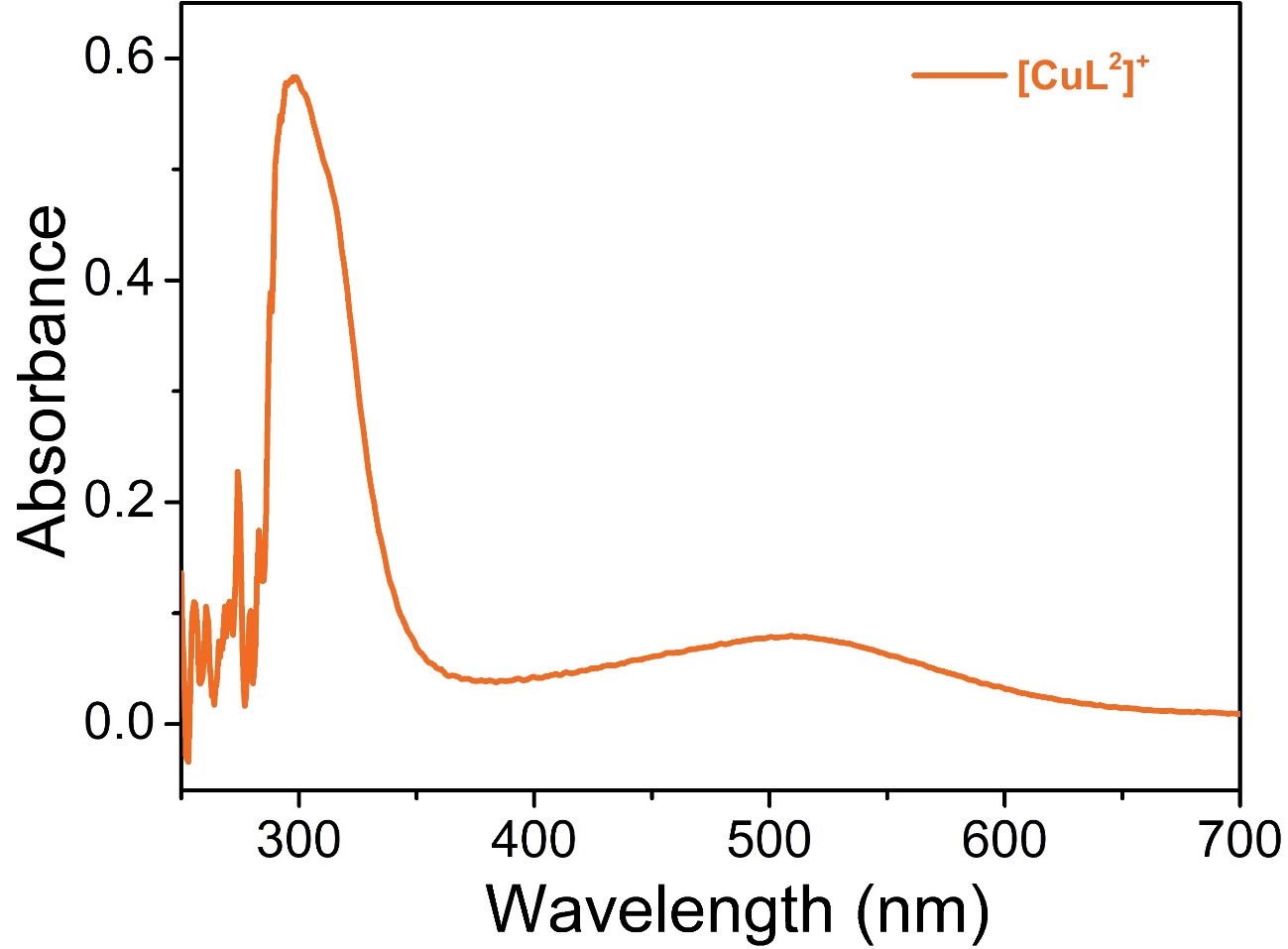


**Figure S33**. UV-vis spectra of 10 μM [CuL^2^]^+^ in CH_3_OH solution.


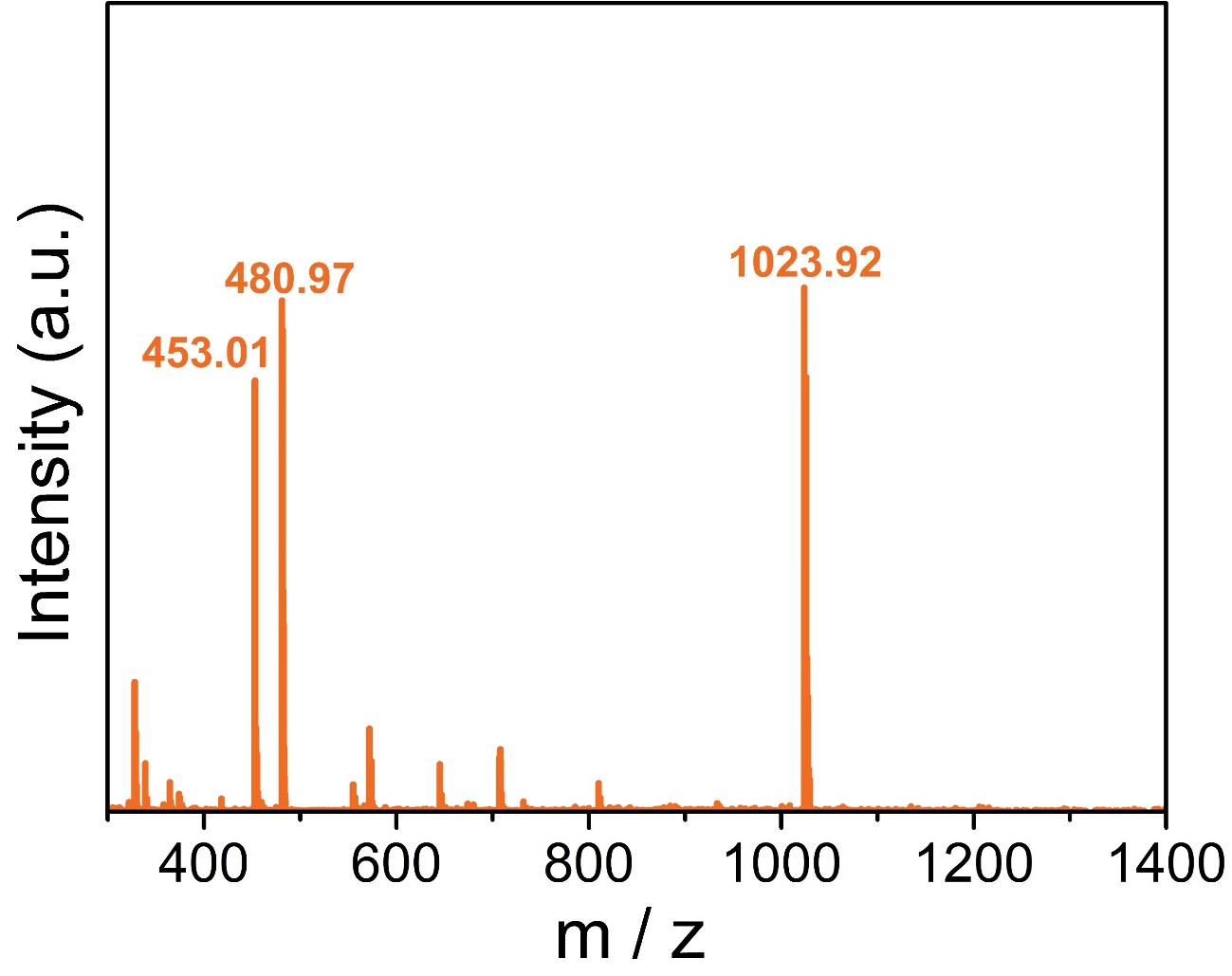


**Figure S34**. LC-MS of [NiL^2^]^2+^ in CH_3_OH. m/z = 453.01 [L^2^+H^+^]^+^; m/z = 480.97 [NiL^2^]^2+^; m/z = 1023.92 [[NiL^2^]^2+^+NO_3_^-^]^+^.


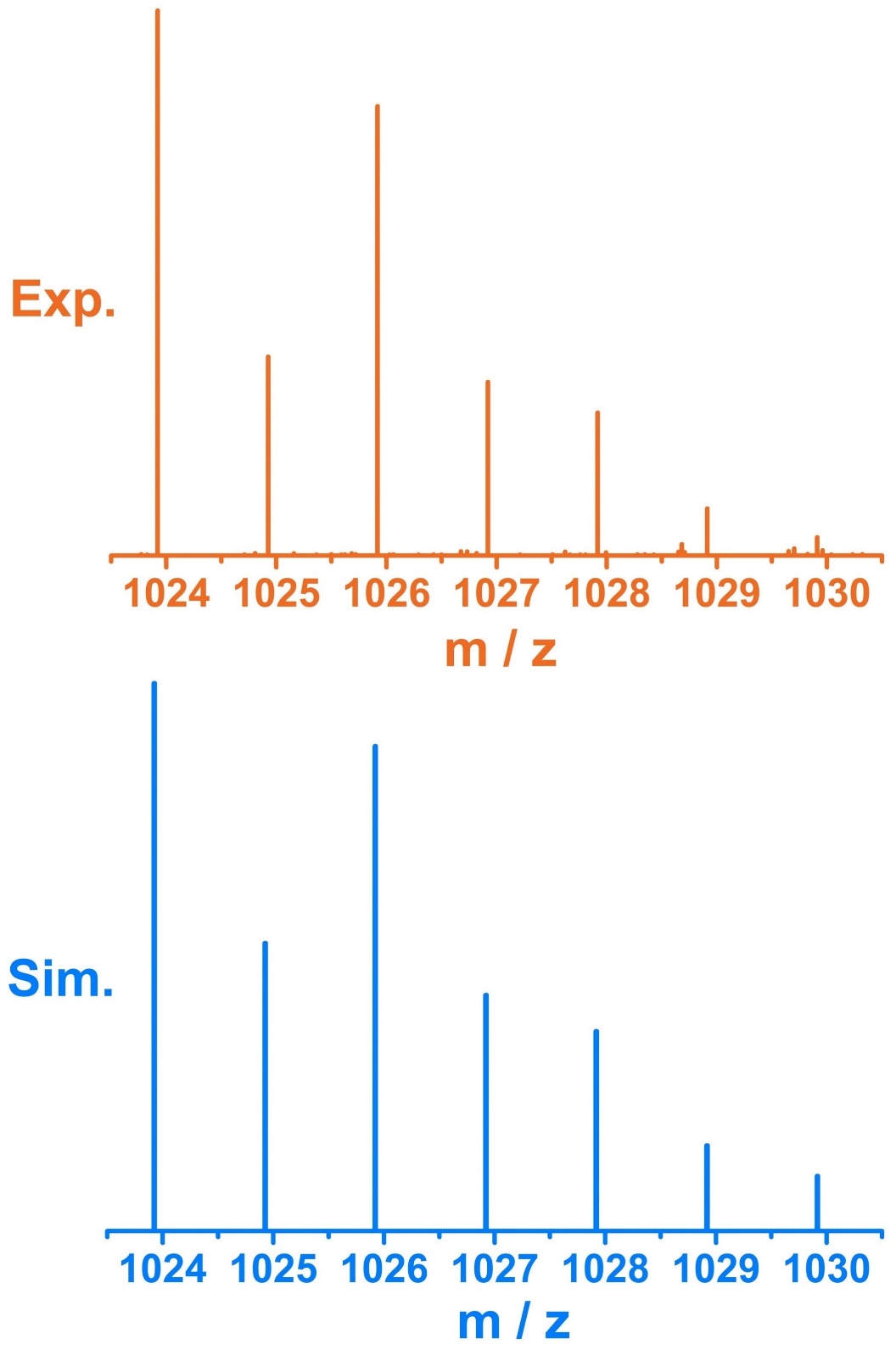


**Figure S35**. Experimental and simulated LC-MS for [NiL^2^]^2+^.


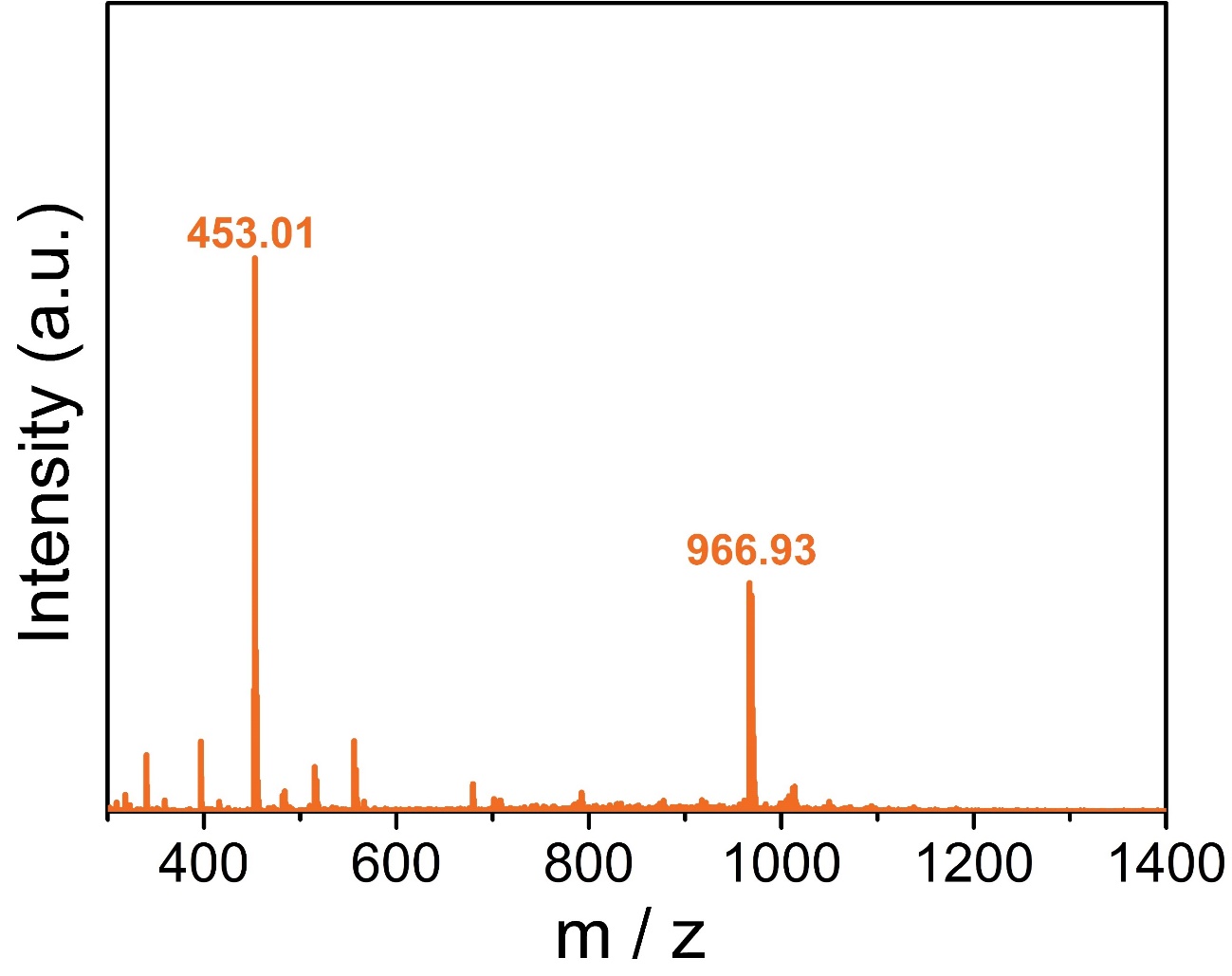


**Figure S36**. LC-MS of [CuL^2^]^+^ in CH_3_OH. m/z = 453.01 [L^2^+H^+^]^+^; m/z = 966.93 [CuL^2^]^+^.


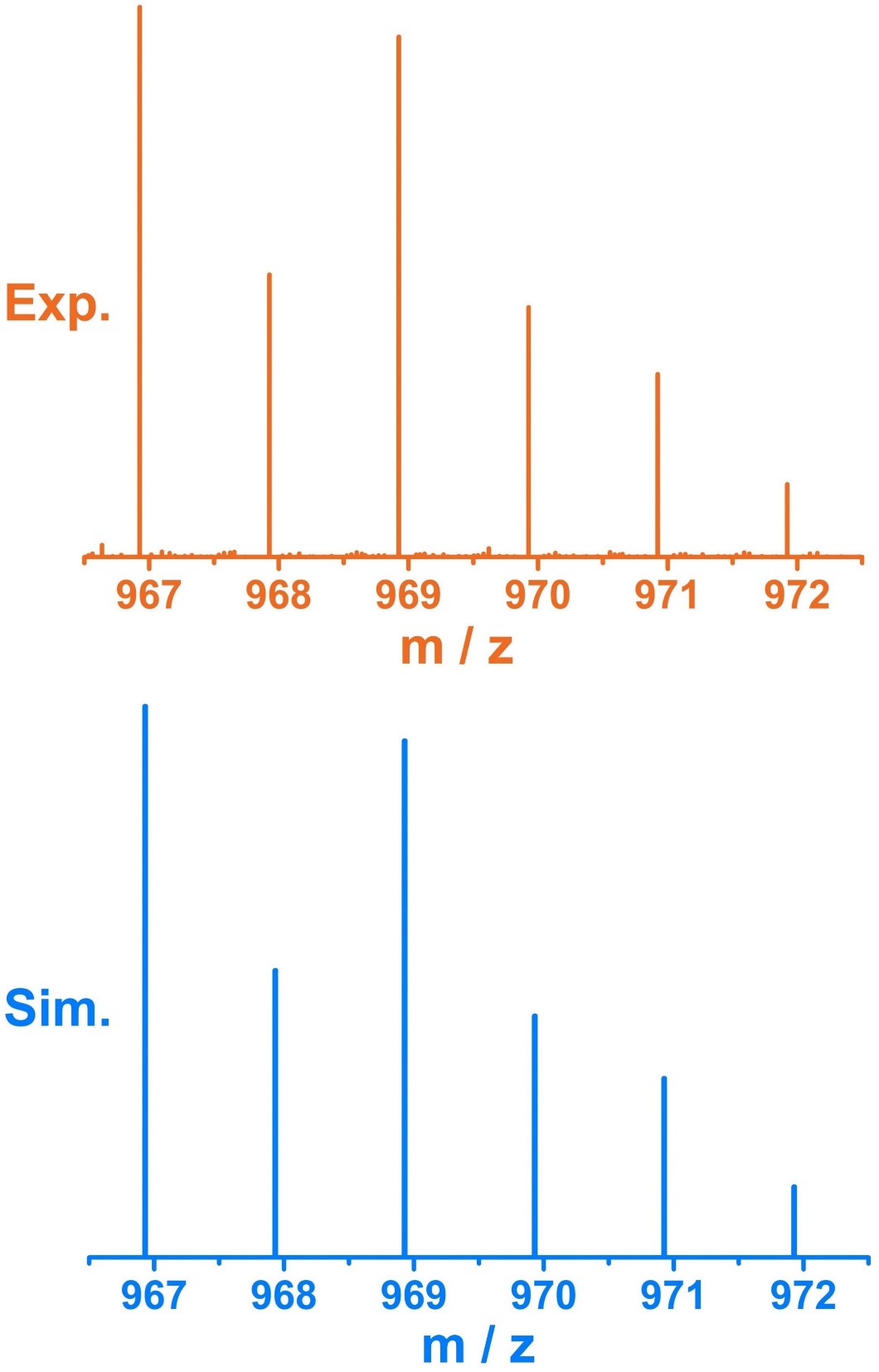


**Figure S37**. Experimental and simulated LC-MS for [CuL^2^]^+^.


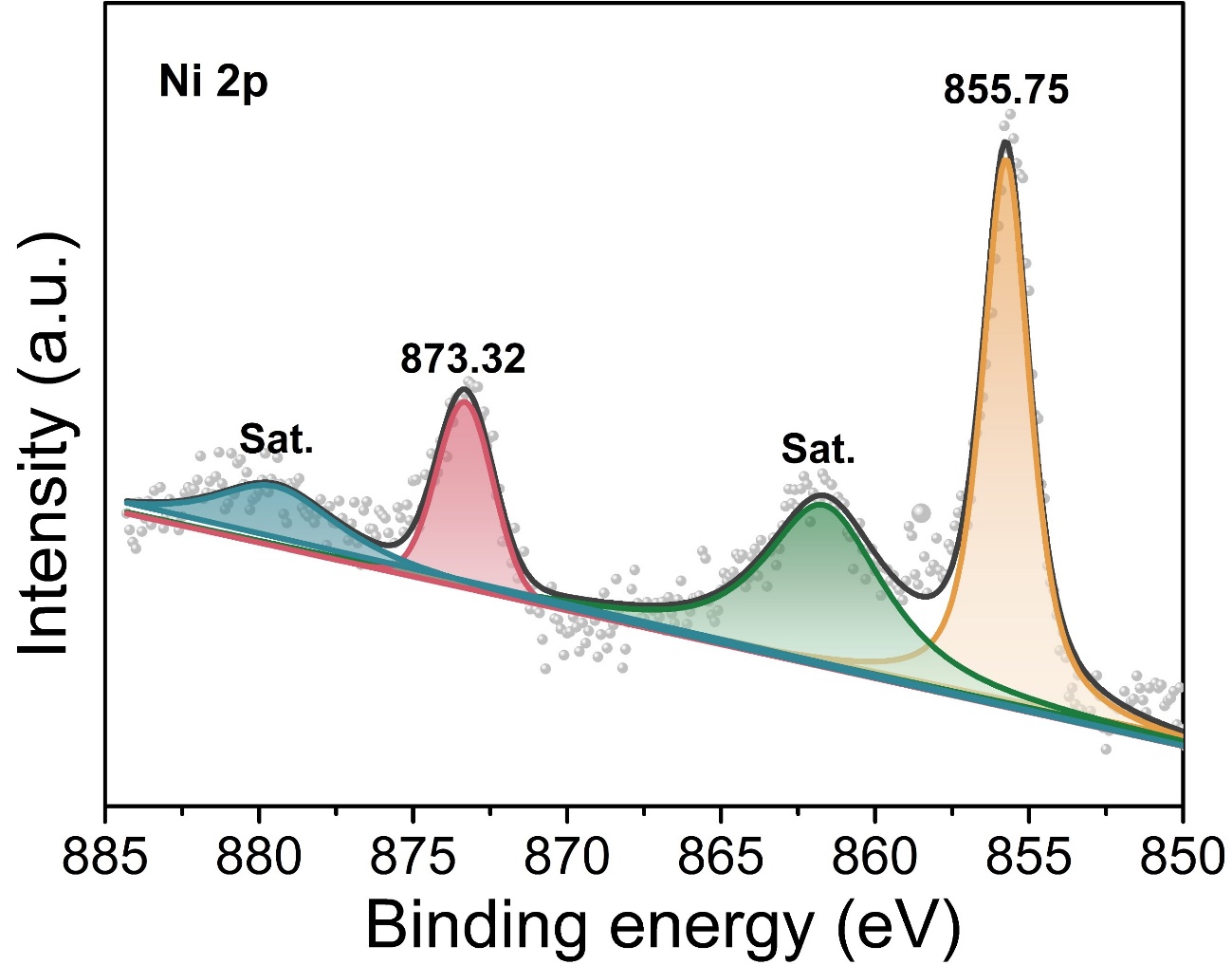


**Figure S38**. Ni 2p XPS spectra of [NiL^2^]^2+^.


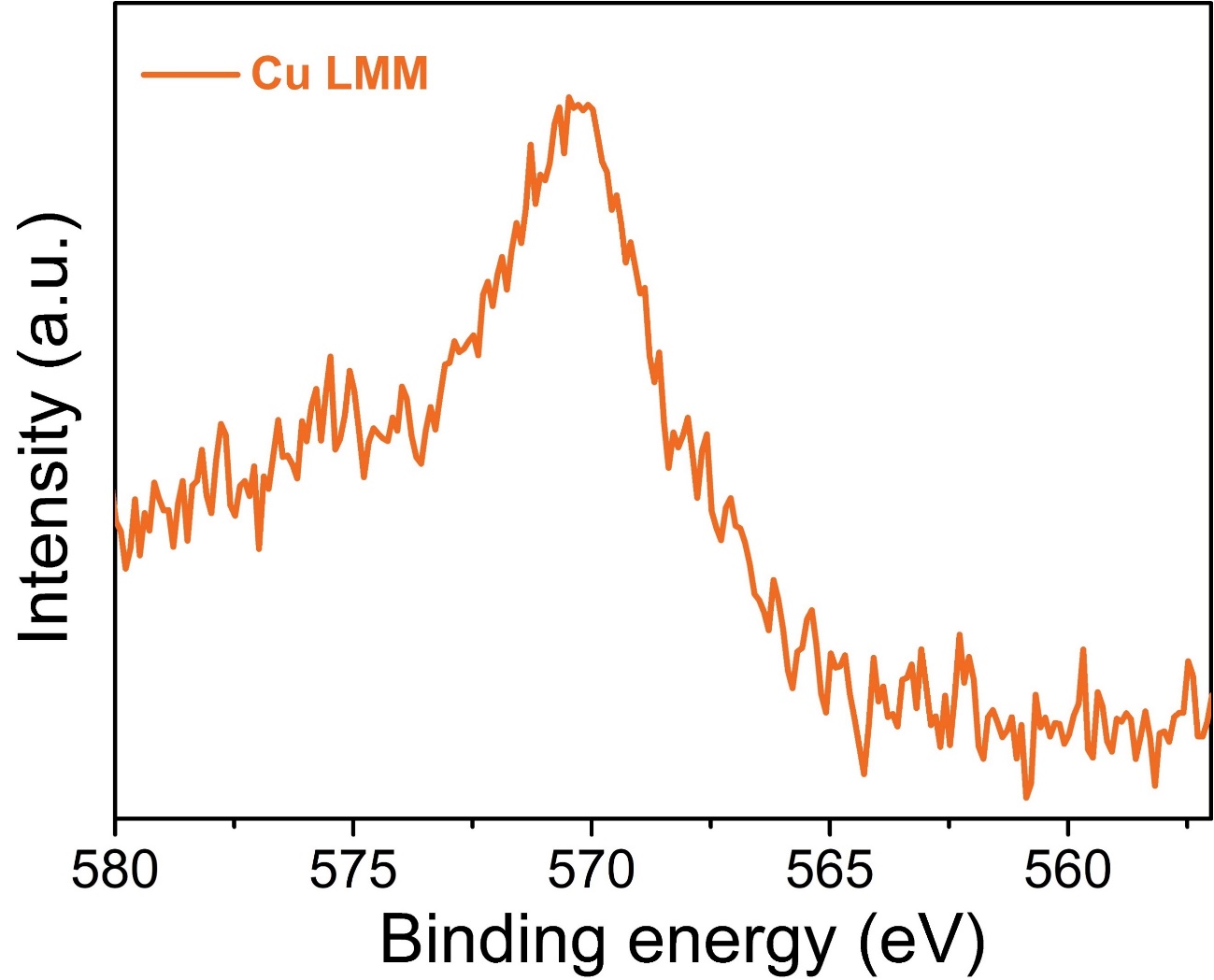


**Figure S39**. Cu LMM Auger spectra of [CuL^2^]^+^.


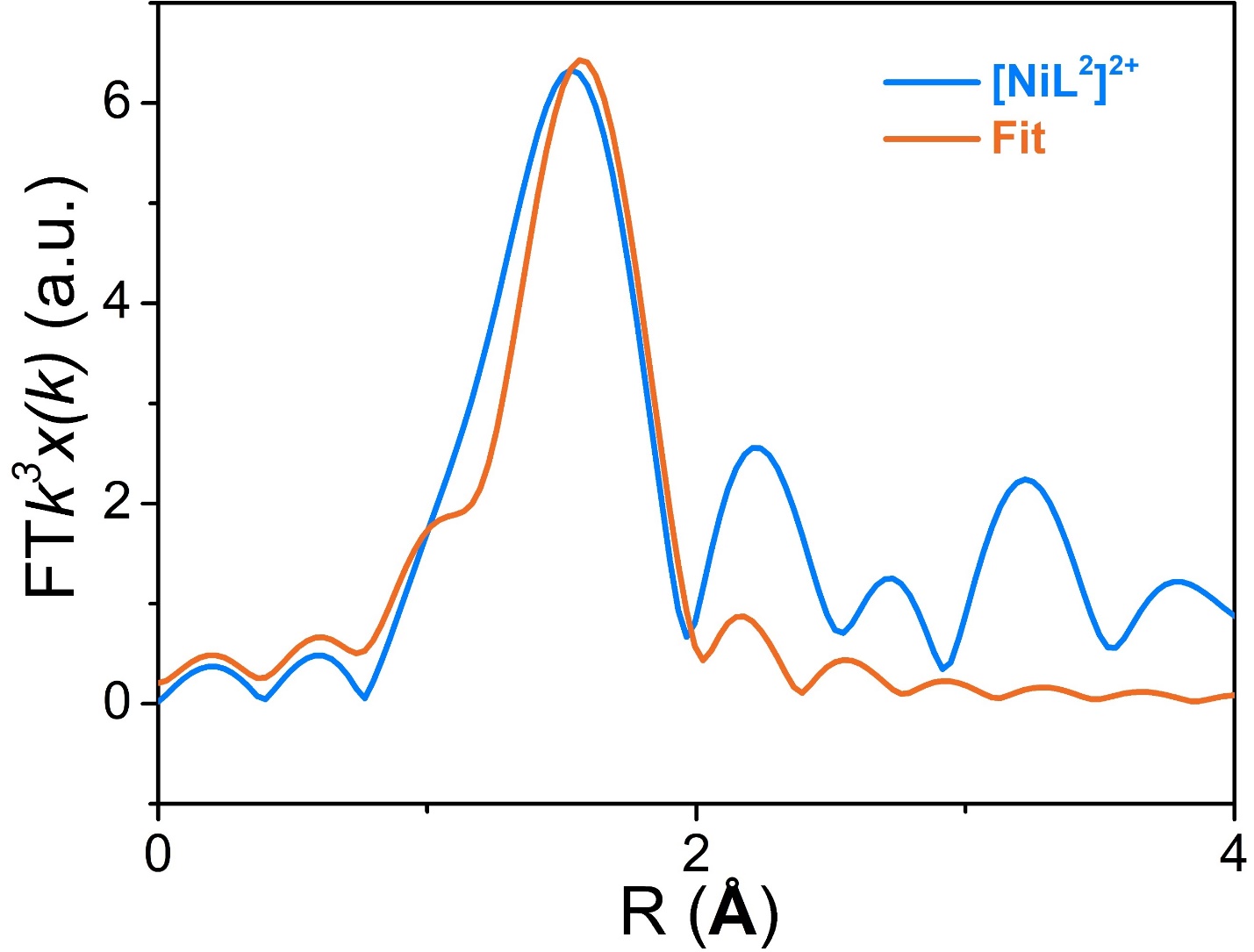


**Figure S40**. Corresponding FT-EXAFS R space fitting result for [NiL^2^]^2+^.


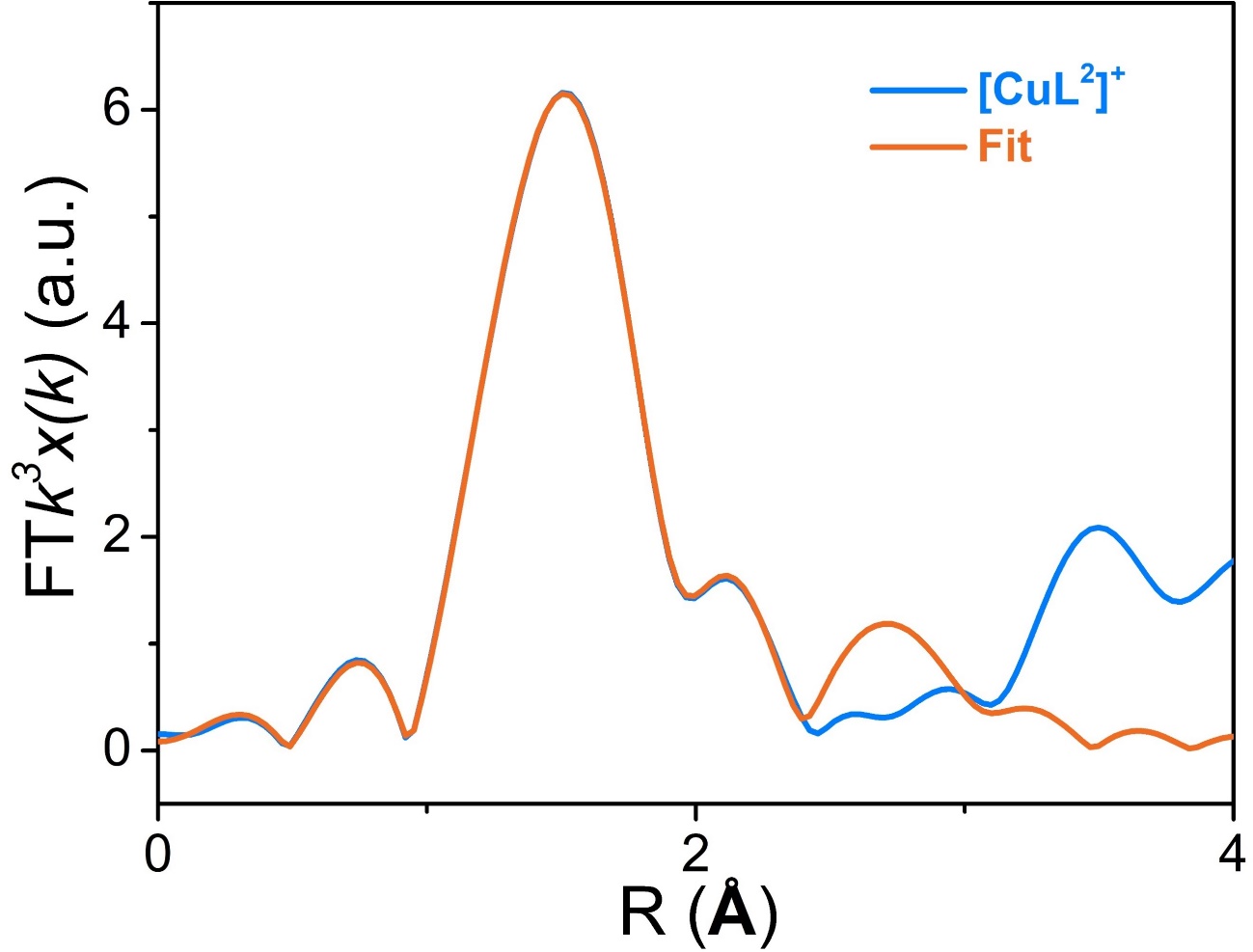


**Figure S41**. Corresponding FT-EXAFS R space fitting result for [CuL^2^]^+^.


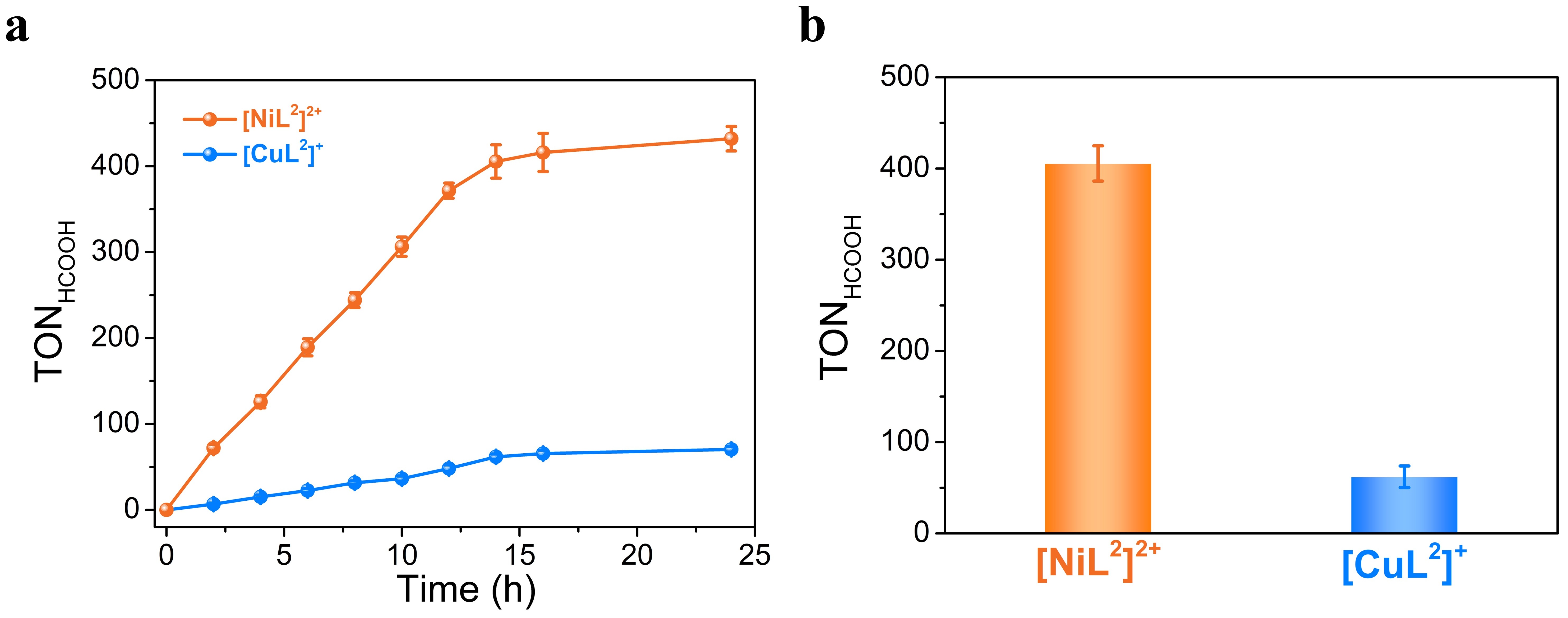


**Figure S42**. (a) Time-dependent HCOOH generation from the photocatalytic CO_2_ reduction coupled with CH_3_OH oxidation over [NiL^2^]^2+^ and [CuL^2^]^+^; (b) Photocatalytic activity of [NiL^2^]^2+^ and [CuL^2^]^+^ for the photocatalytic CO_2_ reduction coupled with CH_3_OH oxidation. Conditions: Catalysts (1 μM), 5 mL CO_2_-saturated CH_3_OH/H_2_O (*v*:*v* = 4:1) solution, 300 W Xe lamp (320 < *λ* < 780 nm; light intensity: 200 mW cm^-2^), 25 ^o^C (The error bars are standard deviations calculated from the results of three parallel experiments).


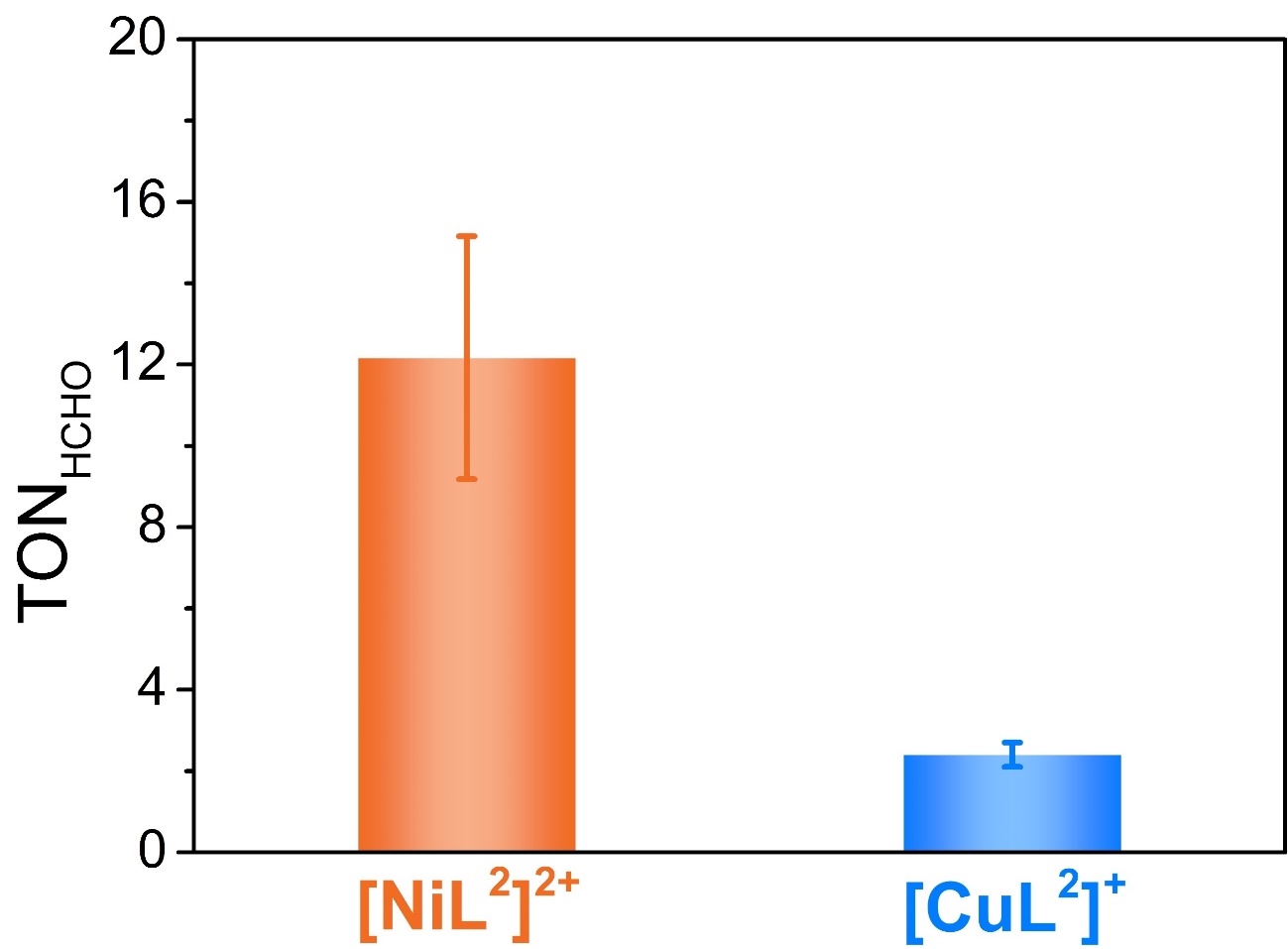


**Figure S43**. Yields of HCHO in photocatalytic CO_2_ reduction coupled with CH_3_OH oxidation by [NiL^2^]^2+^ and [CuL^2^]^+^, respectively. Conditions: [NiL^2^]2+/[CuL^2^]^+^ (1 μM), 5 mL CO_2_-saturated CH_3_OH/H_2_O (*v/v* = 4:1) solution, 300 W Xe lamp (320 < *λ* < 780 nm; light intensity: 200 mW cm^-2^), 14 h, 25 ^o^C (The error bars are standard deviations calculated from the results of three parallel experiments).


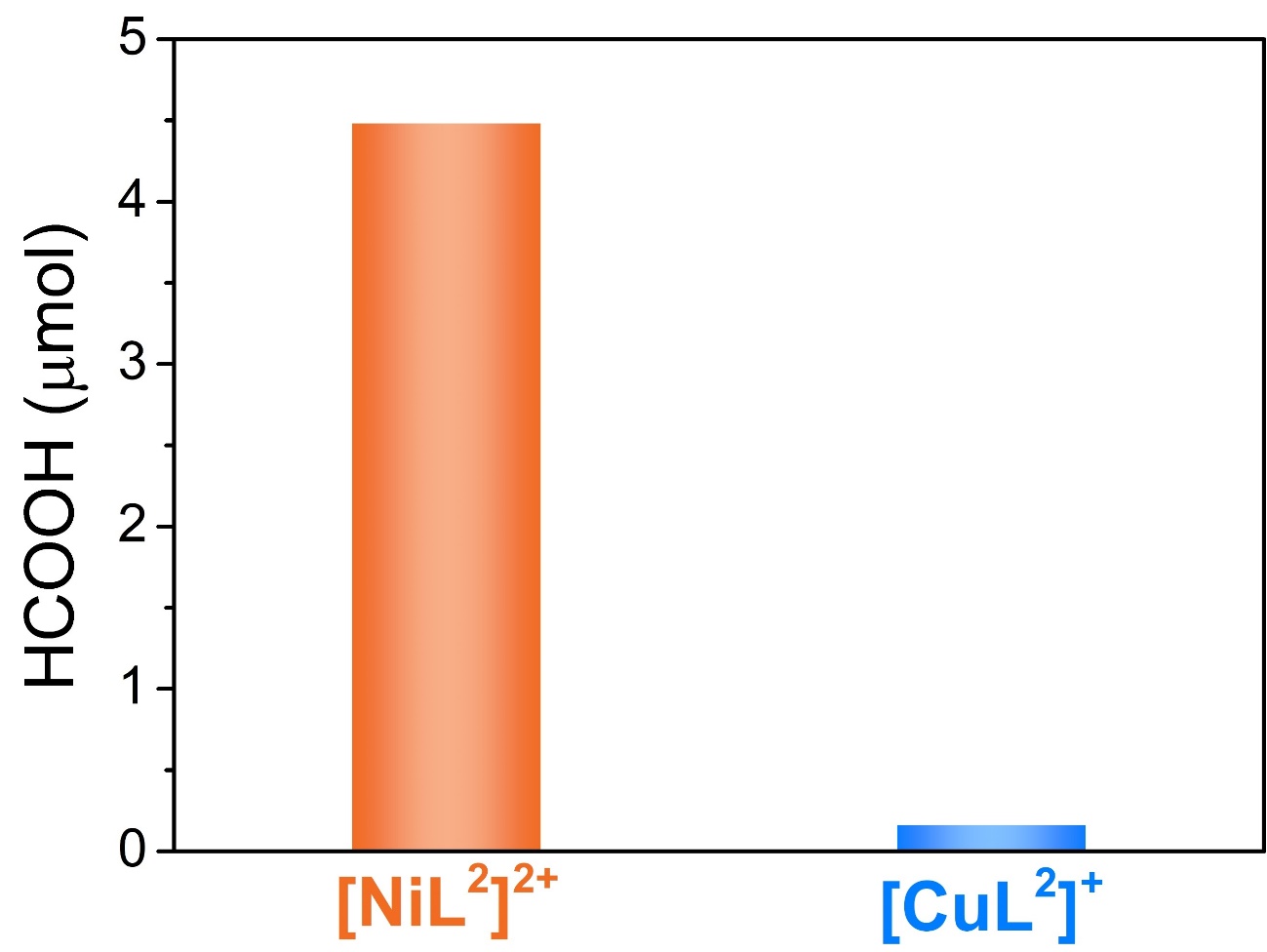


**Figure S44**. Yields of HCOOH in photocatalytic CO_2_ reduction coupled with CH_3_OH oxidation by [NiL^2^]2+/[CuL^2^]^+^ under an LED light (λ = 365 nm) for 10 h. Conditions: [NiL^2^]2+/[CuL^2^]^+^ (1 μM), 5 mL CO_2_-saturated CH_3_OH/H_2_O (*v/v* = 4:1) solution, LED lamp (λ = 365 nm), 10 h, 25 ^o^C.


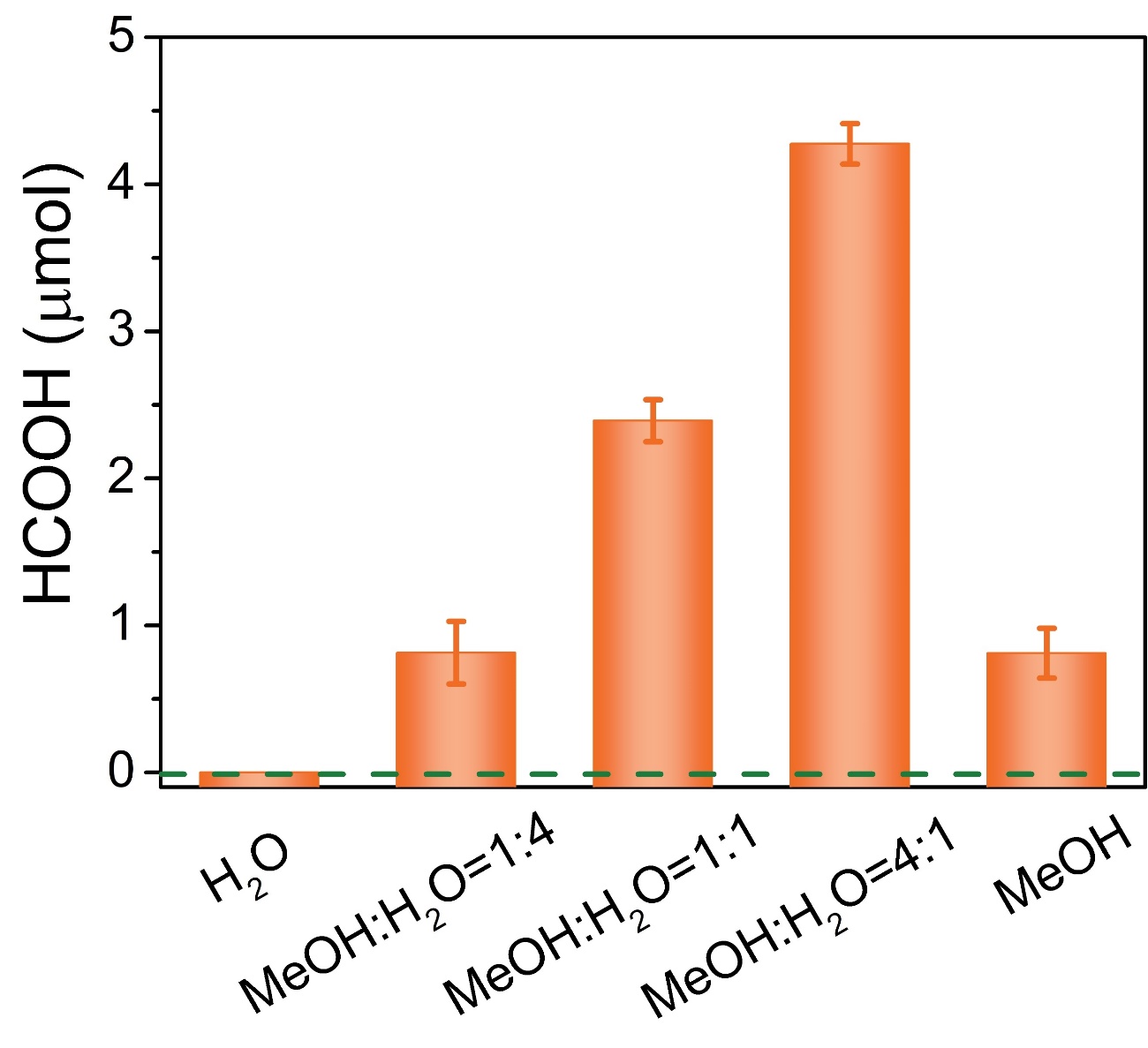


**Figure S45**. Photocatalytic activity of [CoL^2^]^2+^ for HCOOH production under different ratios of CH_3_OH/H_2_O. Conditions: [CoL^2^]^2+^ (1 μM), 5 mL CO_2_-saturated CH_3_OH, CH_3_OH/H_2_O, or H_2_O solution, 300 W Xe lamp (320 < *λ* < 780 nm; light intensity: 200 mW cm^-2^), 14 h, 25 ^o^C (The error bars are standard deviations calculated from the results of three parallel experiments).


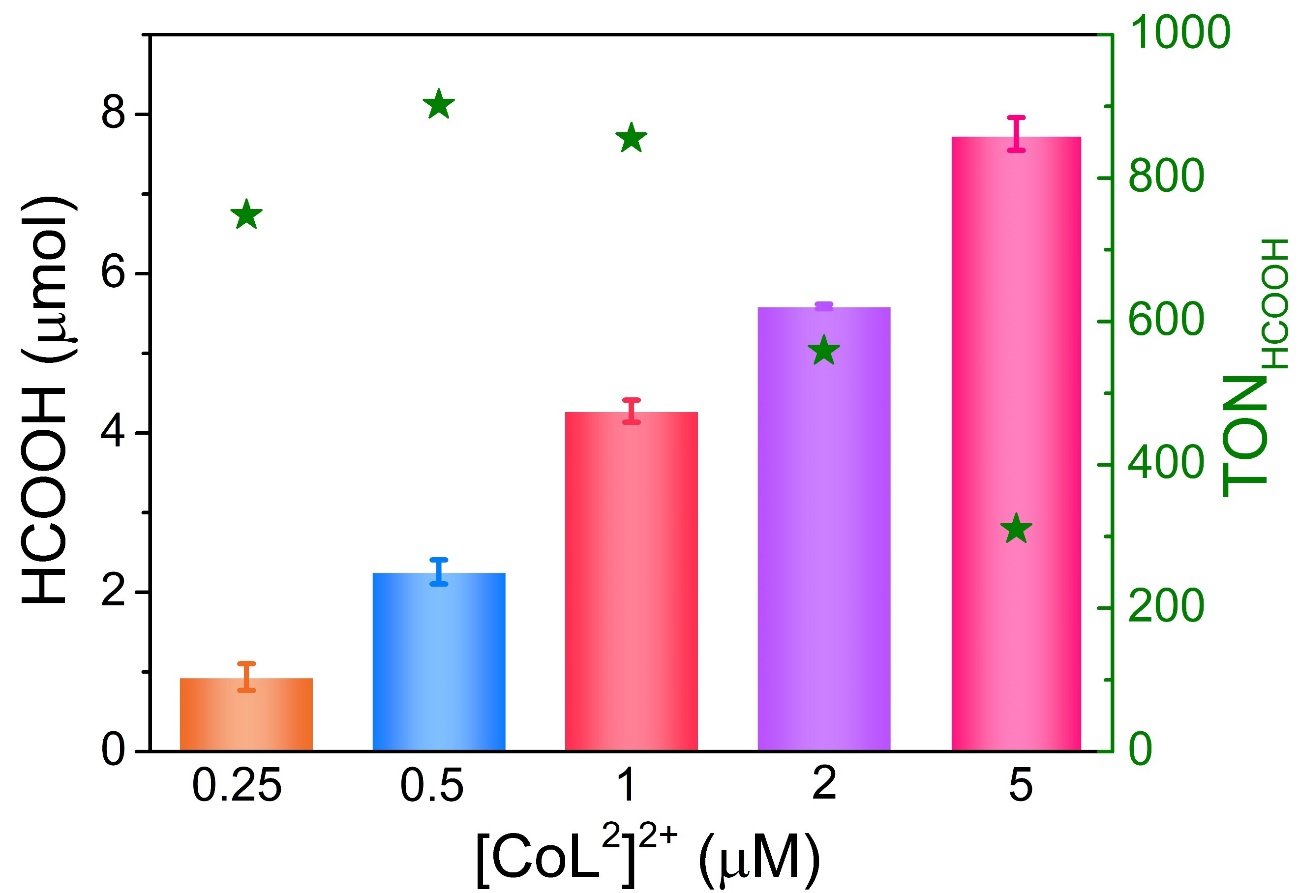


**Figure S46**. Photocatalytic evolution of HCOOH in 5 mL CO_2_-saturated CH_3_OH/H_2_O (*v*/*v* = 4:1) solution catalyzed by [CoL^2^]^2+^ at a concentration of 0.25, 0.5, 1, 2, and 5 μM. Reaction conditions: [CoL^2^]^2+^ (0.25, 0.5, 1, 2, and 5 μM), 5 mL CO_2_-saturated CH_3_OH/H_2_O (*v/v* = 4:1) solution, 300 W Xe lamp (320 < *λ* < 780 nm; light intensity: 200 mW cm^-2^), 14 h, 25 ^o^C (The error bars are standard deviations calculated from the results of three parallel experiments).


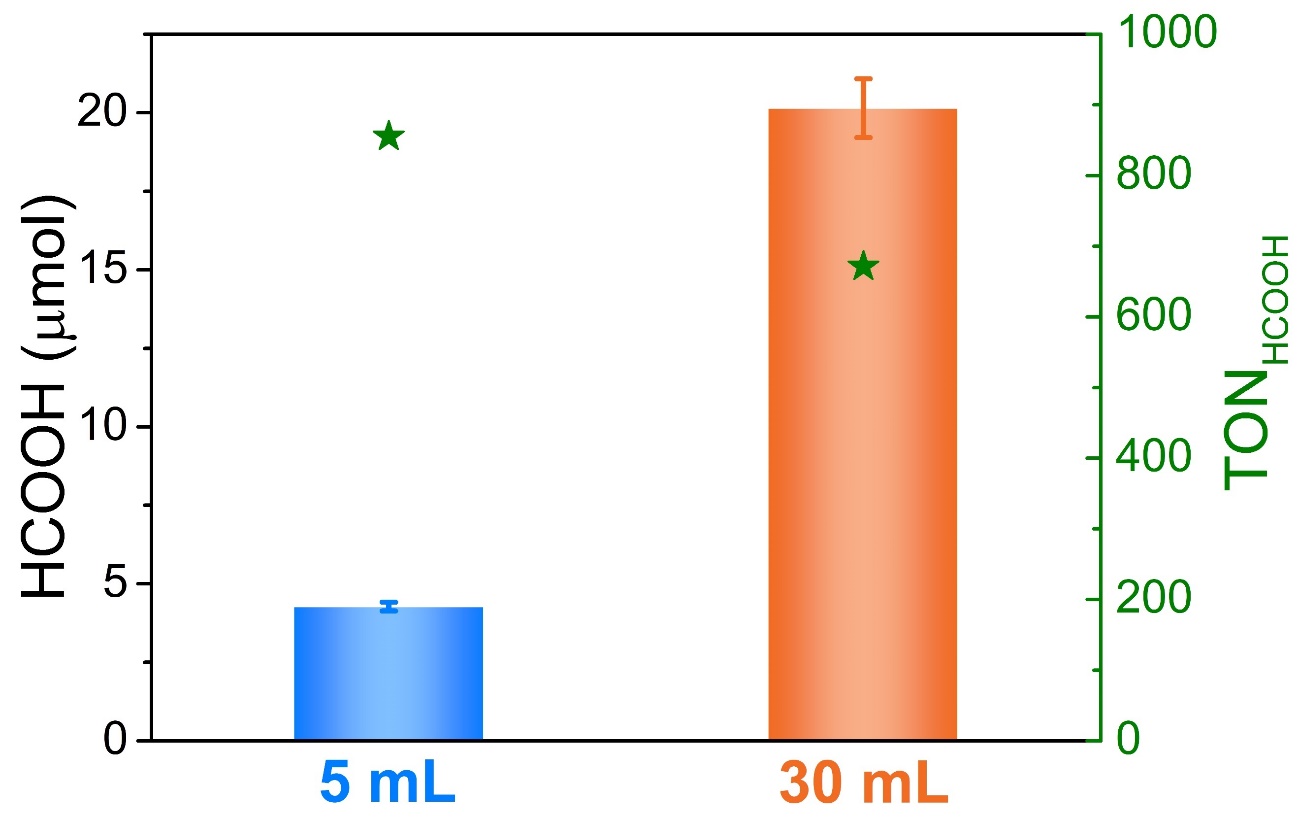


**Figure S47**. Photocatalytic evolution of HCOOH in 5 or 30 mL CO_2_-saturated CH_3_OH/H_2_O (*v*/*v* = 4:1) solution catalyzed by [CoL^2^]^2+^. Reaction conditions: [CoL^2^]^2+^ (1 μM), CO_2_-saturated CH_3_OH/H_2_O (*v/v* = 4:1) solution, 300 W Xe lamp (320 < *λ* < 780 nm; light intensity: 200 mW cm^-2^), 14 h, 25 ^o^C (The error bars are standard deviations calculated from the results of three parallel experiments).


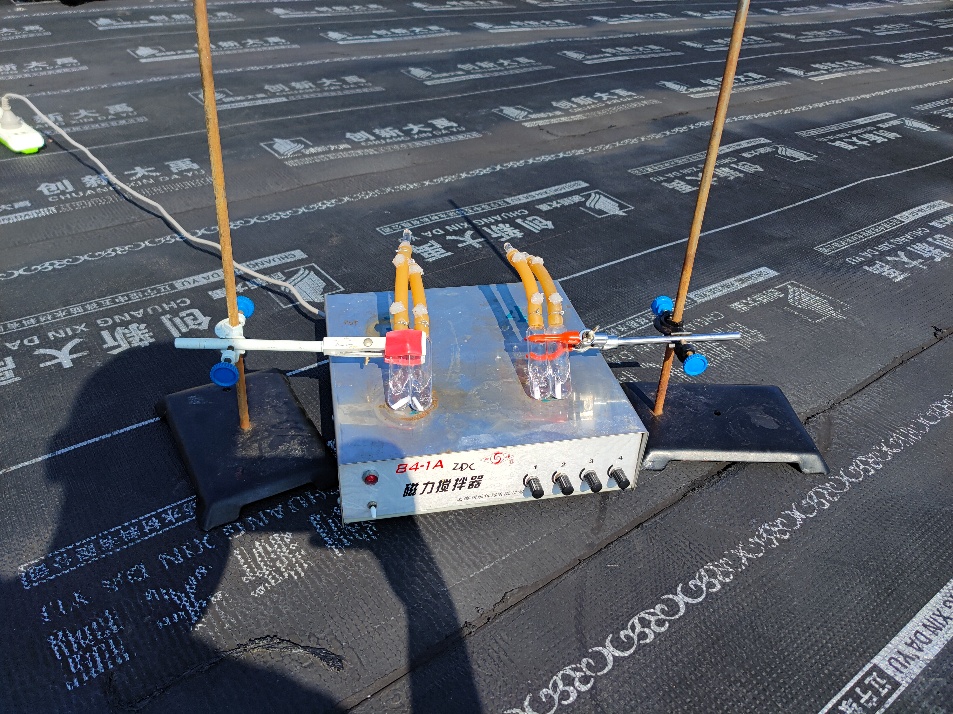


**Figure S48**. Setup used for photocatalytic CO_2_ reduction coupled with CH_3_OH oxidation under sunlight illumination. Conditions: [CoL^2^]^2+^ (1 μM), 5 mL CO_2_-saturated CH_3_OH/H_2_O (*v/v* = 4:1) solution. The experiments were performed with [CoL^2^]^2+^ under sunlight from 9:00 am to 5:00 pm for one week from 9^th^ to 15^th^ August 2023 on the rooftop of our institute.


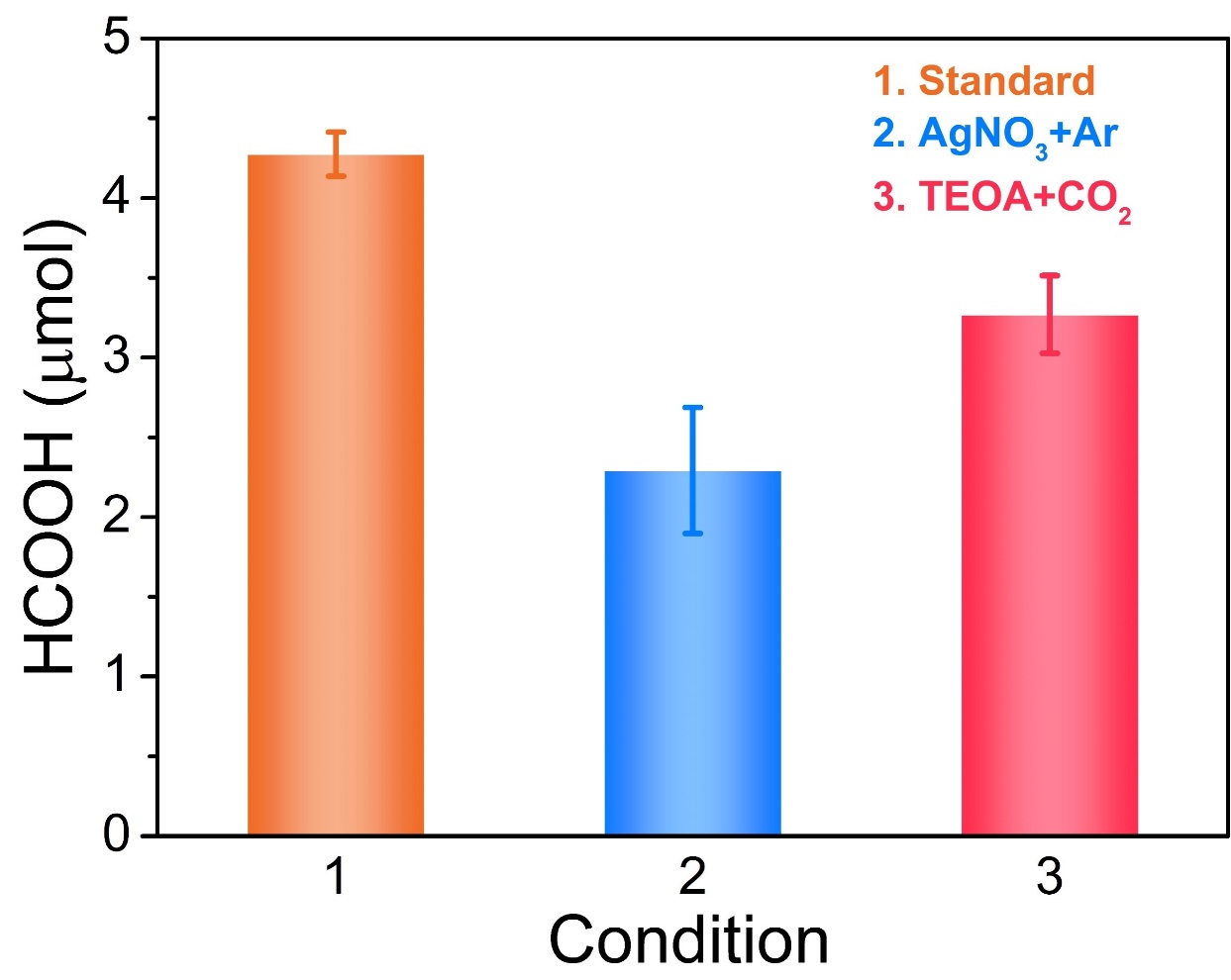


**Figure S49**. Amounts of HCOOH produced on [CoL^2^]^2+^ in different conditions. Conditions: [CoL^2^]^2+^ (1 μM), 300 W Xe lamp (320 < *λ* < 780 nm; light intensity: 200 mW cm^-2^), 14 h, 25 ^o^C. **1,** 5 mL CO_2_-saturated CH_3_OH/H_2_O (*v/v* = 4:1) solution; **2,** 5 mL Ar-saturated CH_3_OH/H_2_O (*v/v* = 4:1) solution containing AgNO_3_ (10 mM); **3,** 5 mL CO_2_-saturated DMF/H_2_O (*v/v* = 4:1) solution containing TEOA (30 mM) (The error bars are standard deviations calculated from the results of three parallel experiments).


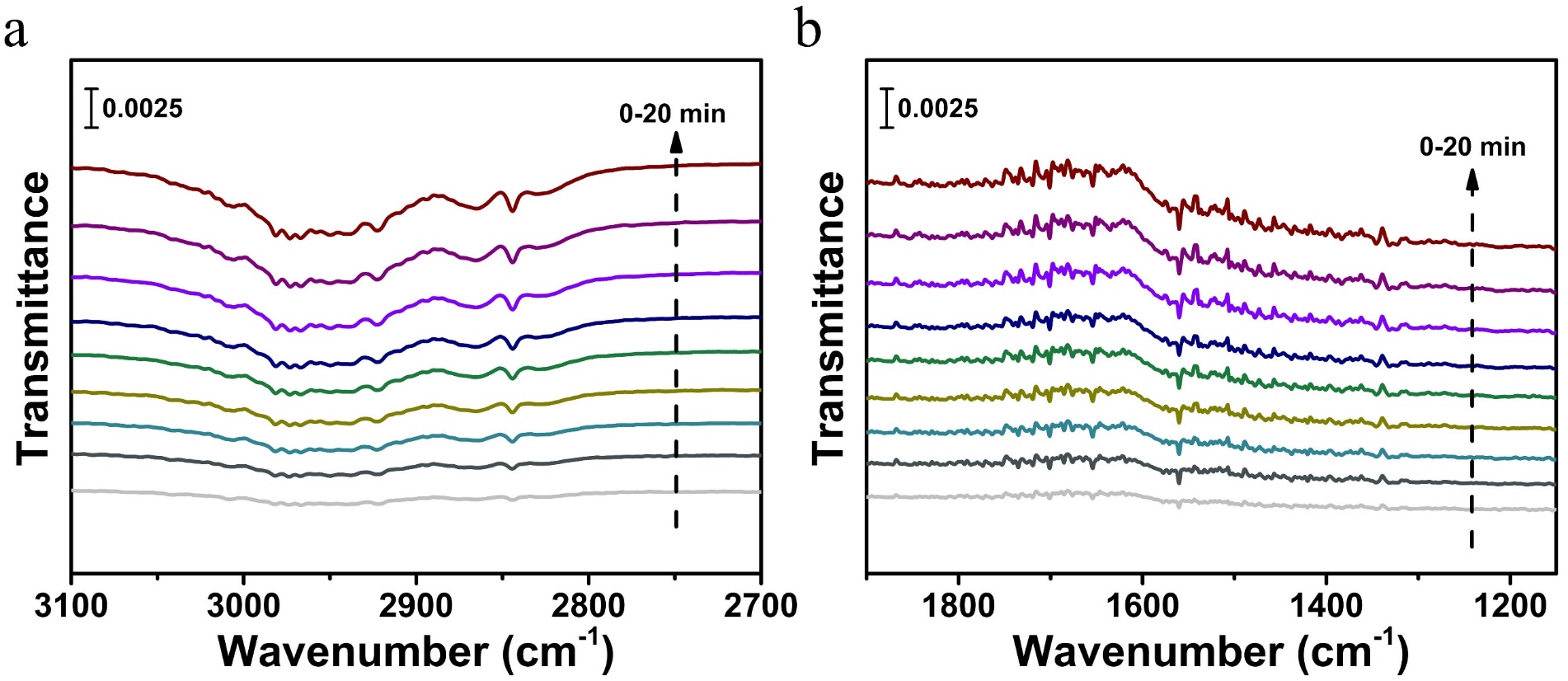


**Figure S50**. Operando FTIR spectra of [CoL^2^]^2+^ in CO_2_ atmosphere without CH_3_OH for intermediates upon increasing illumination times, (a) 2700-3100 cm^-1^ and (b) 1150-1900 cm^-1^.


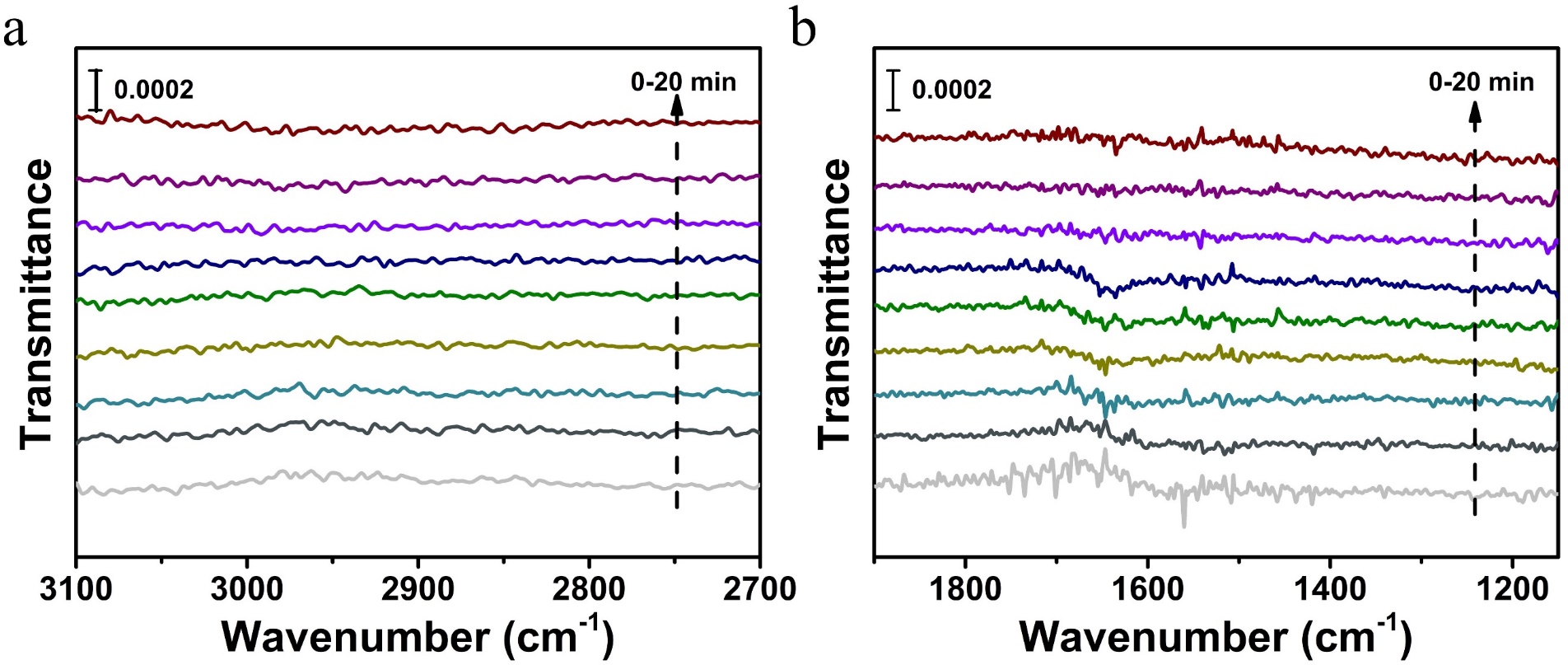


**Figure S51**. Operando FTIR spectra of [CoL^2^]^2+^ in N_2_ atmosphere for intermediates recorded upon increasing illumination times, (a) 2700-3100 cm^-1^ and (b) 1150-1900 cm^-1^.


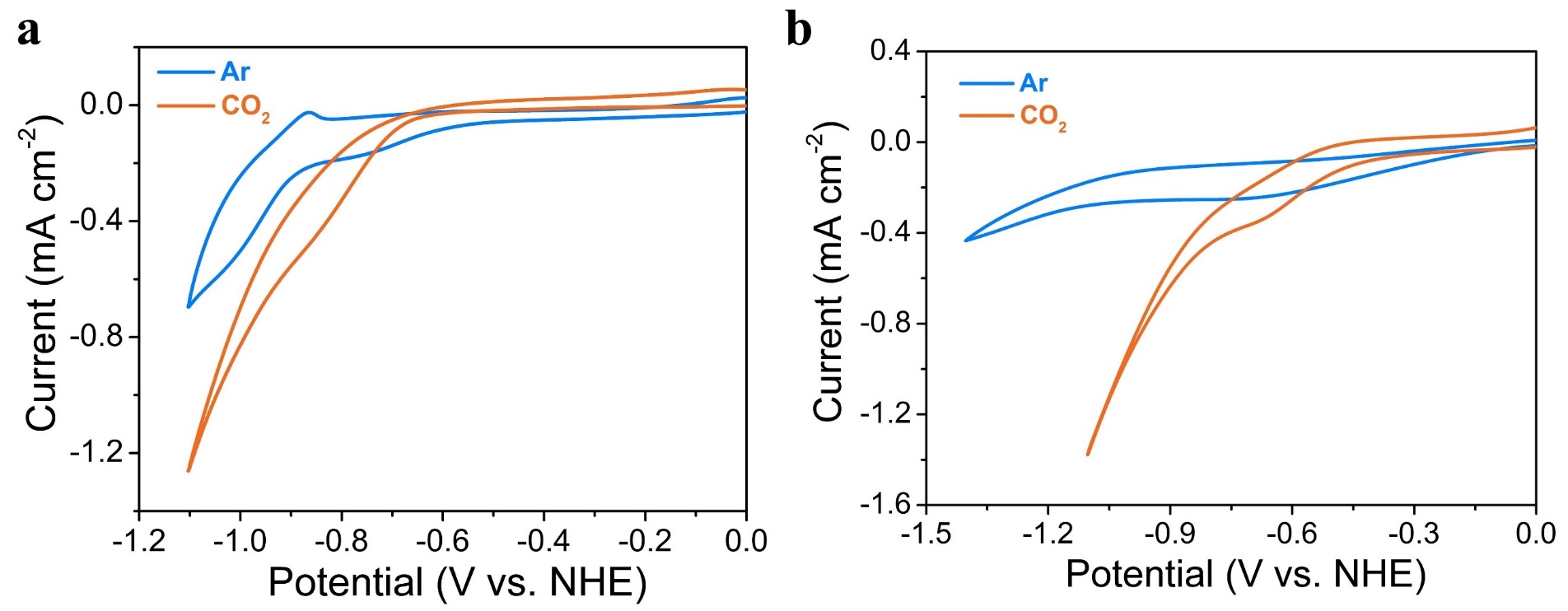


**Figure S52**. CVs of (a) [CoL^1^]^2+^ and (b) [CoL^2^]^2+^ (0.5 mM) in CH_3_OH/H_2_O solution under Ar and CO_2_ atmosphere, respectively.


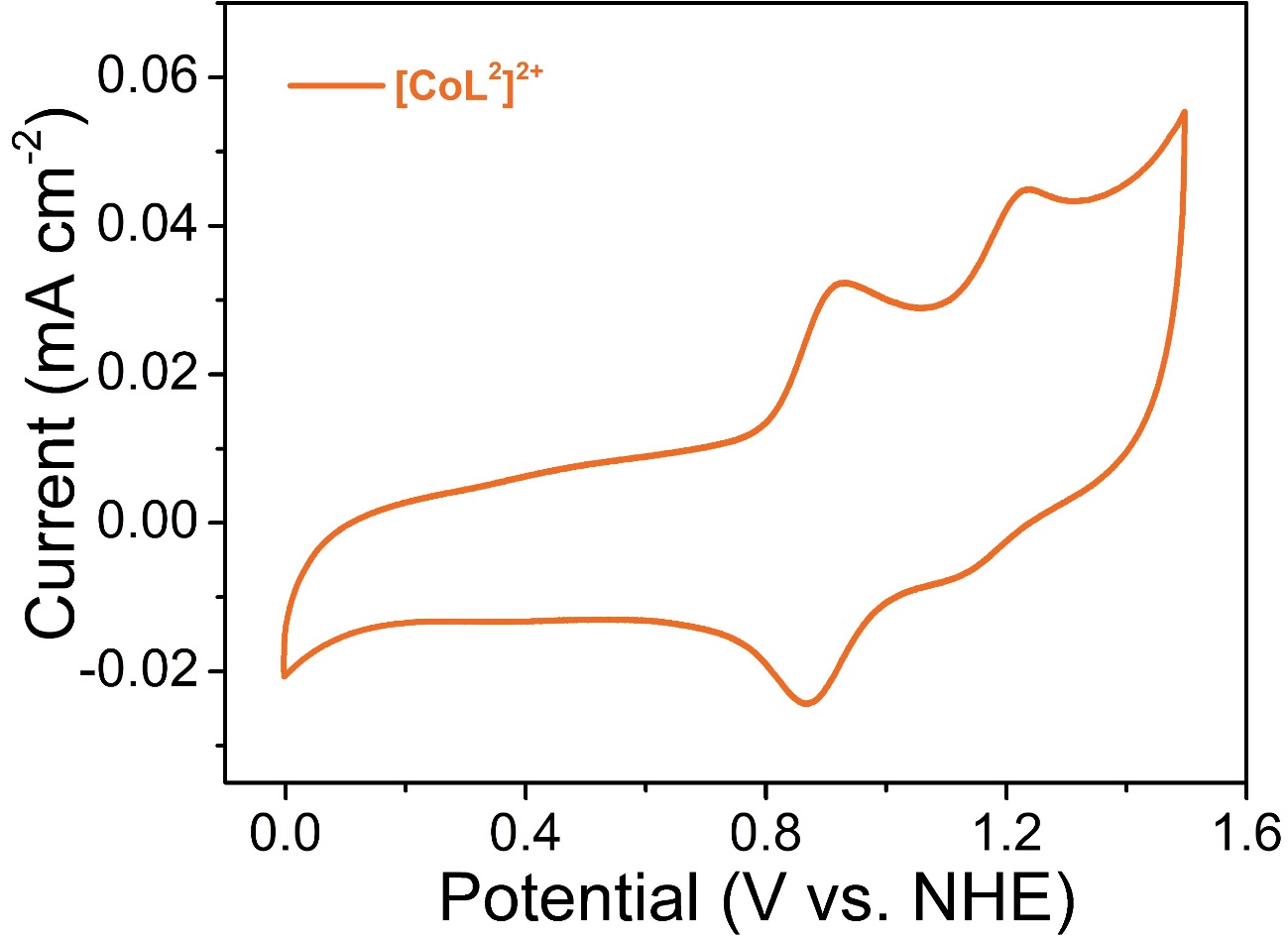


**Figure S53**. CVs of [CoL^2^]^2+^ (0.5 mM) in CH_3_OH/H_2_O solution under Ar atmosphere.


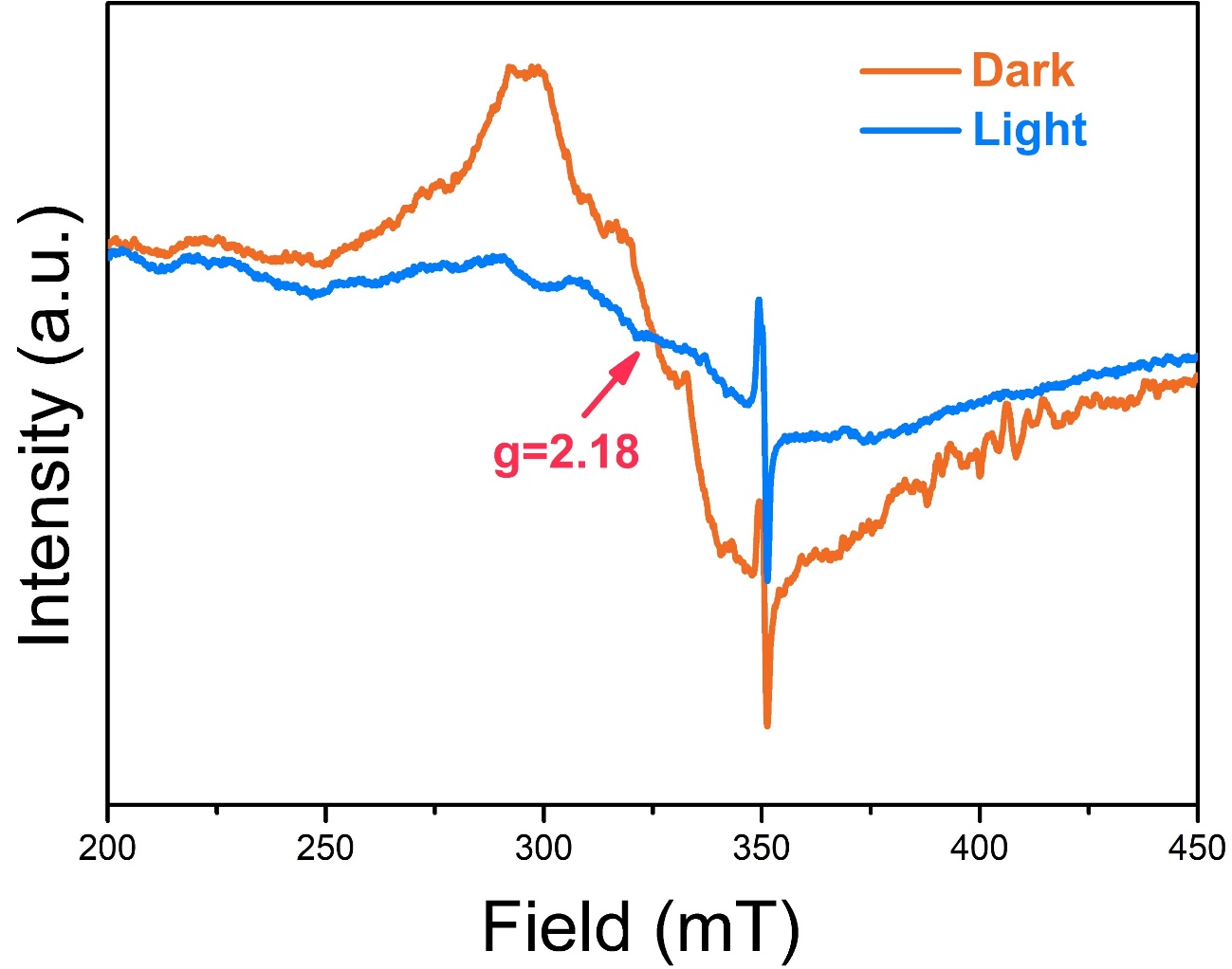


**Figure S54**. EPR spectra of Co species in [CoL^2^]^2+^ before and after illumination by a Xe lamp.


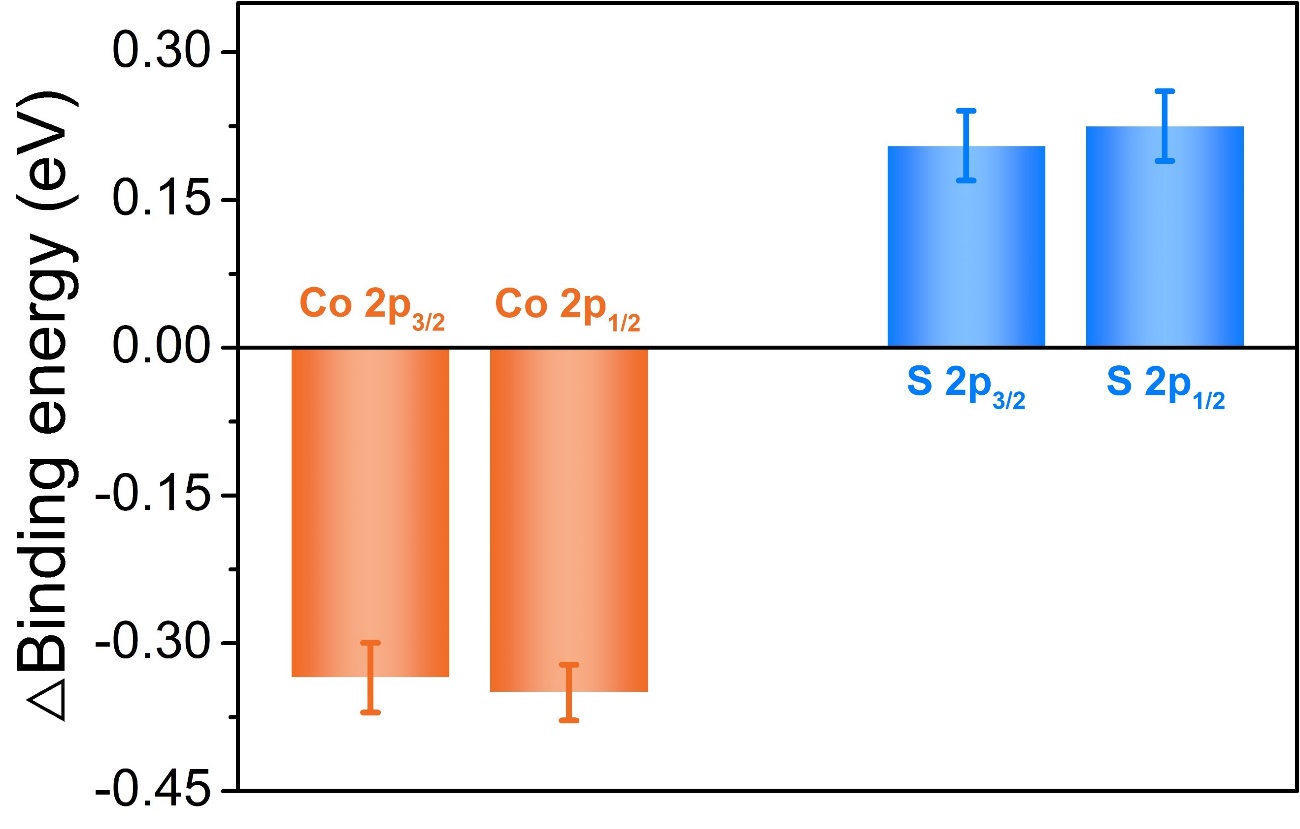


**Figure S55**. Light-induced binding energy shift (*Δ*binding energy) in [CoL^2^]^2+^ (The error bars are standard deviations calculated from the results of three parallel experiments).


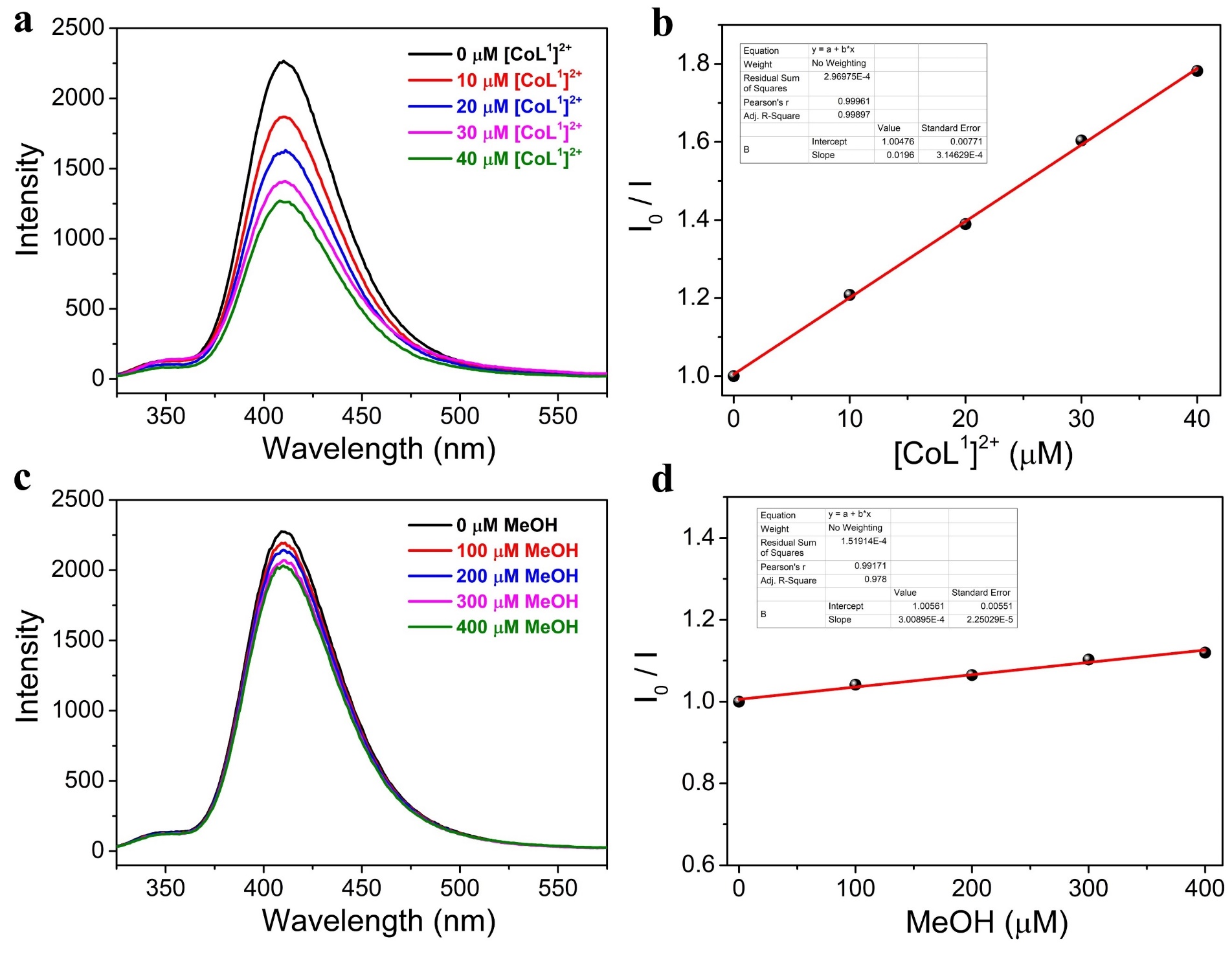


**Figure S56**. Emission intensity of TTF (400 µM) in deaerated CH_3_CN solution upon excitation at 300 nm with increasing contents of (a, c) [CoL^1^]^2+^ or CH_3_OH and (b, d) corresponding Stern-Volmer equations.


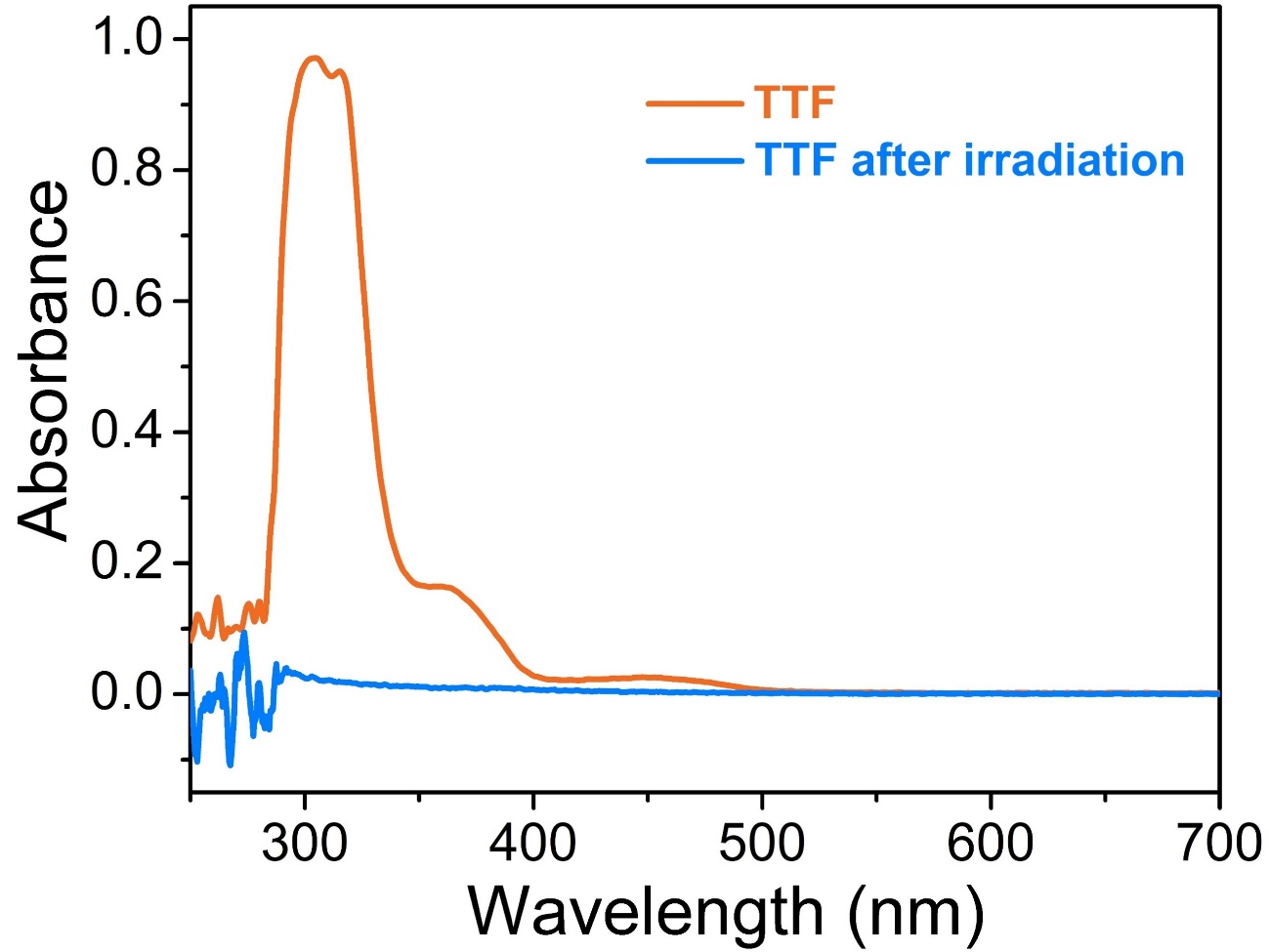


**Figure S57**. UV-vis absorption spectra of TTF (100 μM) before and after illumination for 14 h in CH_3_OH/H_2_O (*v*/*v* = 4:1) solution.


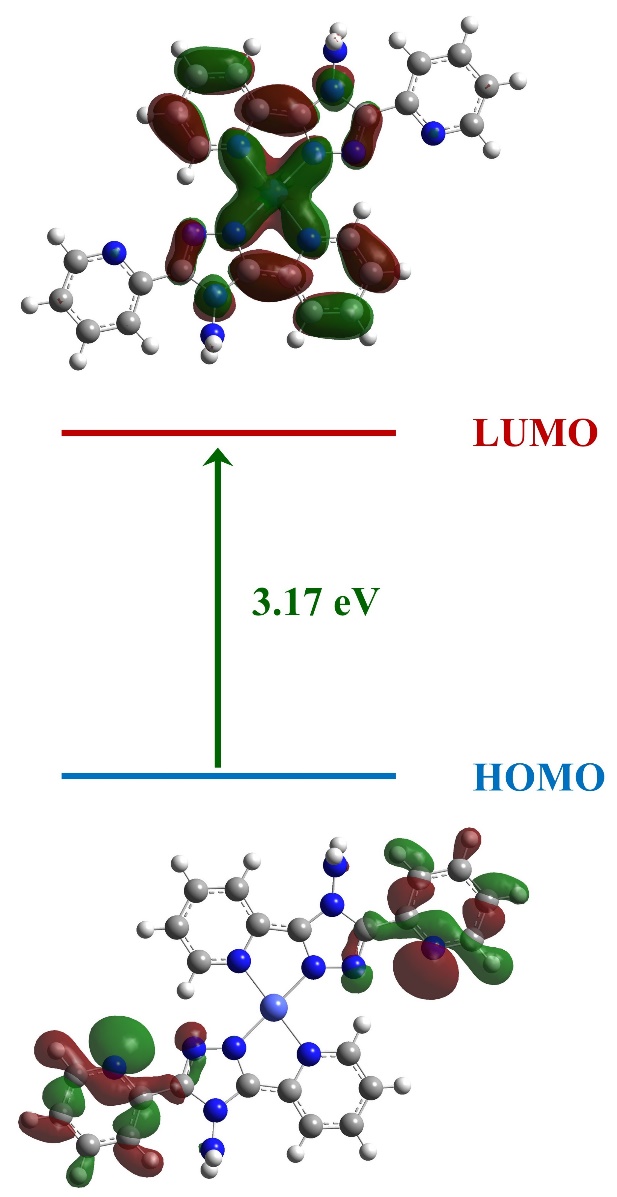


**Figure S58**. Calculated HOMO-LUMO gap of [CoL^1^]^2+^.


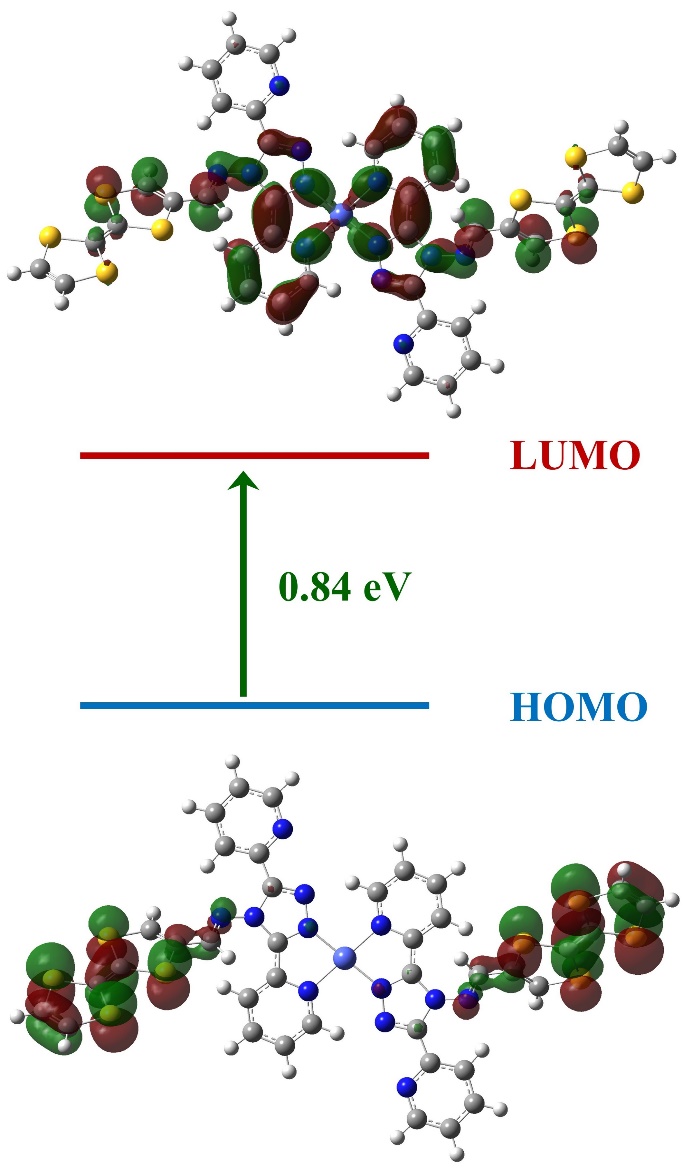


**Figure S59**. Calculated HOMO-LUMO gap of [CoL^2^]^2+^.


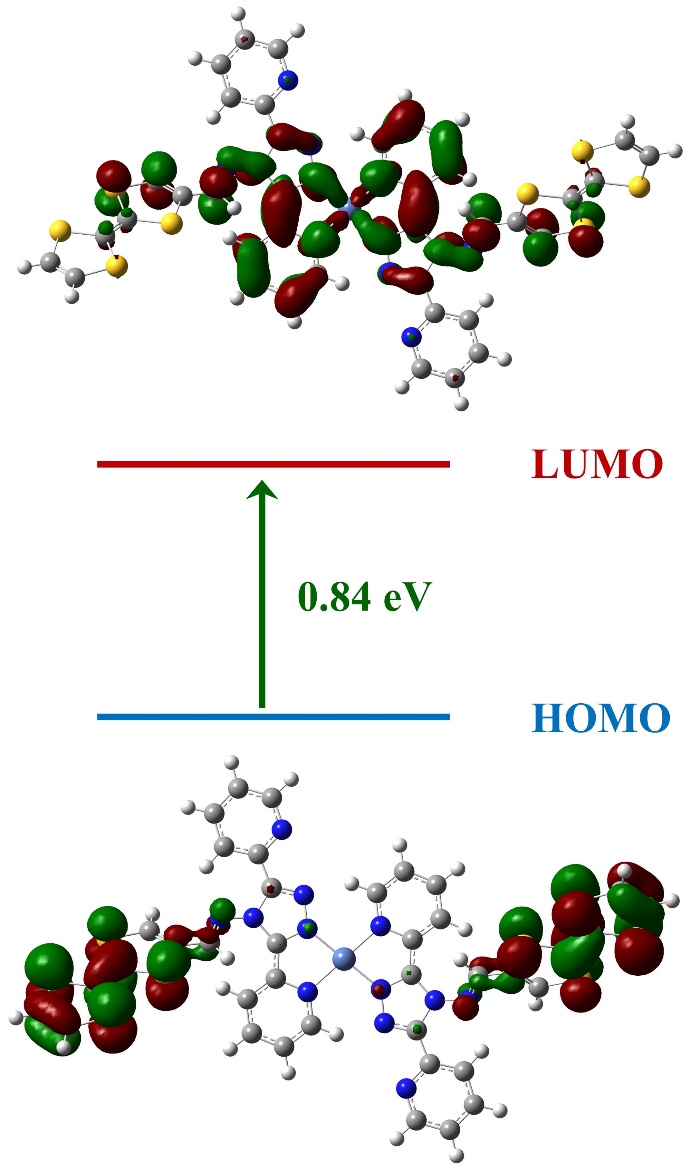


**Figure S60**. Calculated HOMO-LUMO gap of [NiL^2^]^2+^.


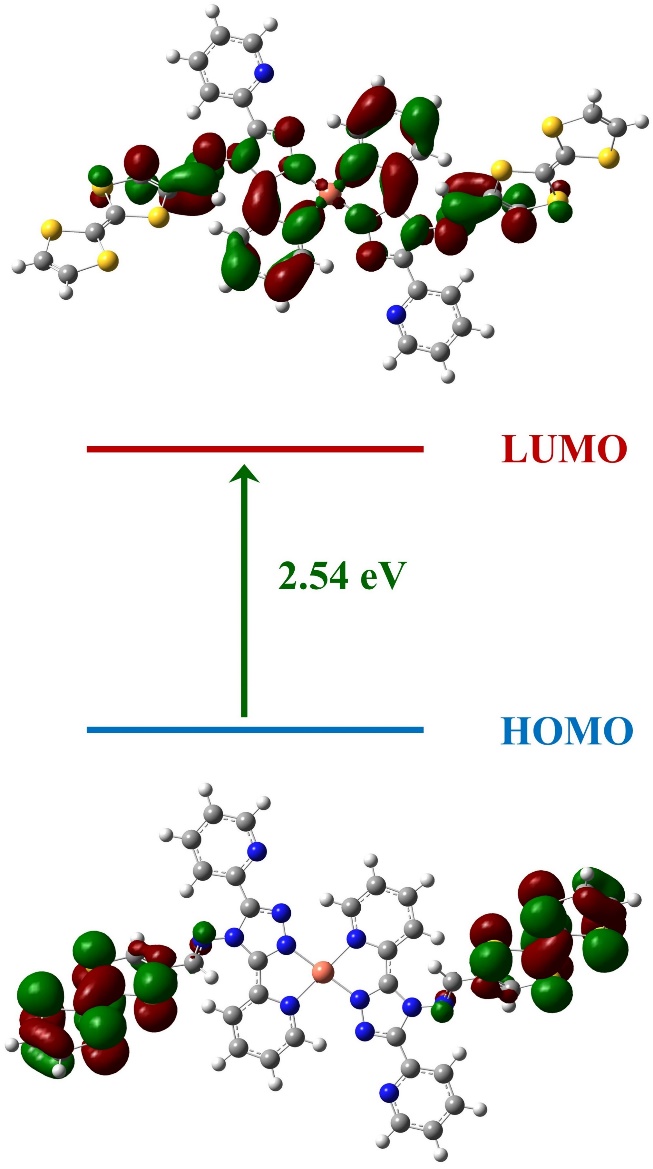


**Figure S61**. Calculated HOMO-LUMO gap of [CuL^2^]^+^.


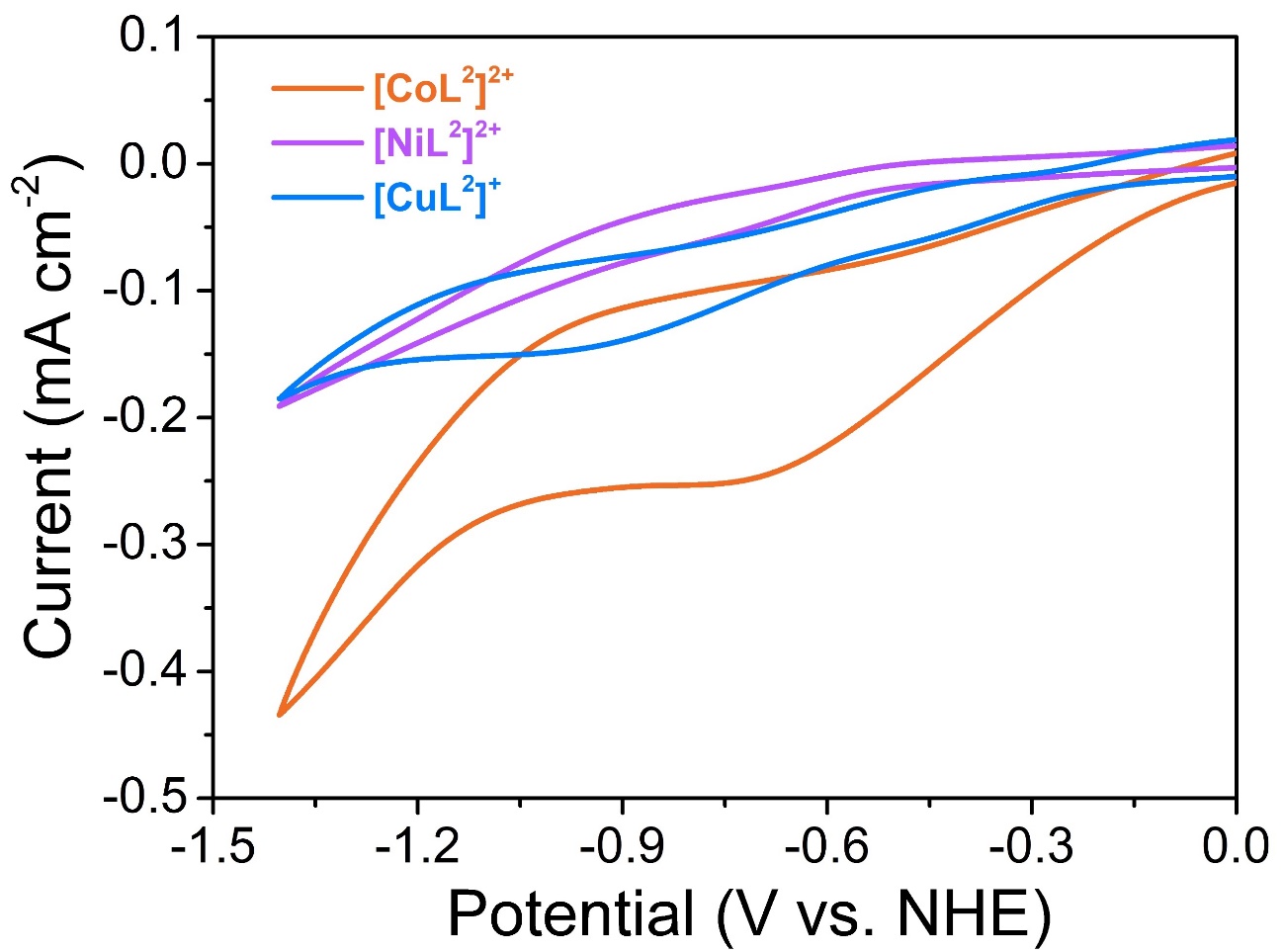


**Figure S62**. CVs of [CoL^2^]^2+^, [NiL^2^]^2+^ and [CuL^2^]^+^ (0.5 mM) in CH_3_OH/H_2_O solution under Ar atmosphere.


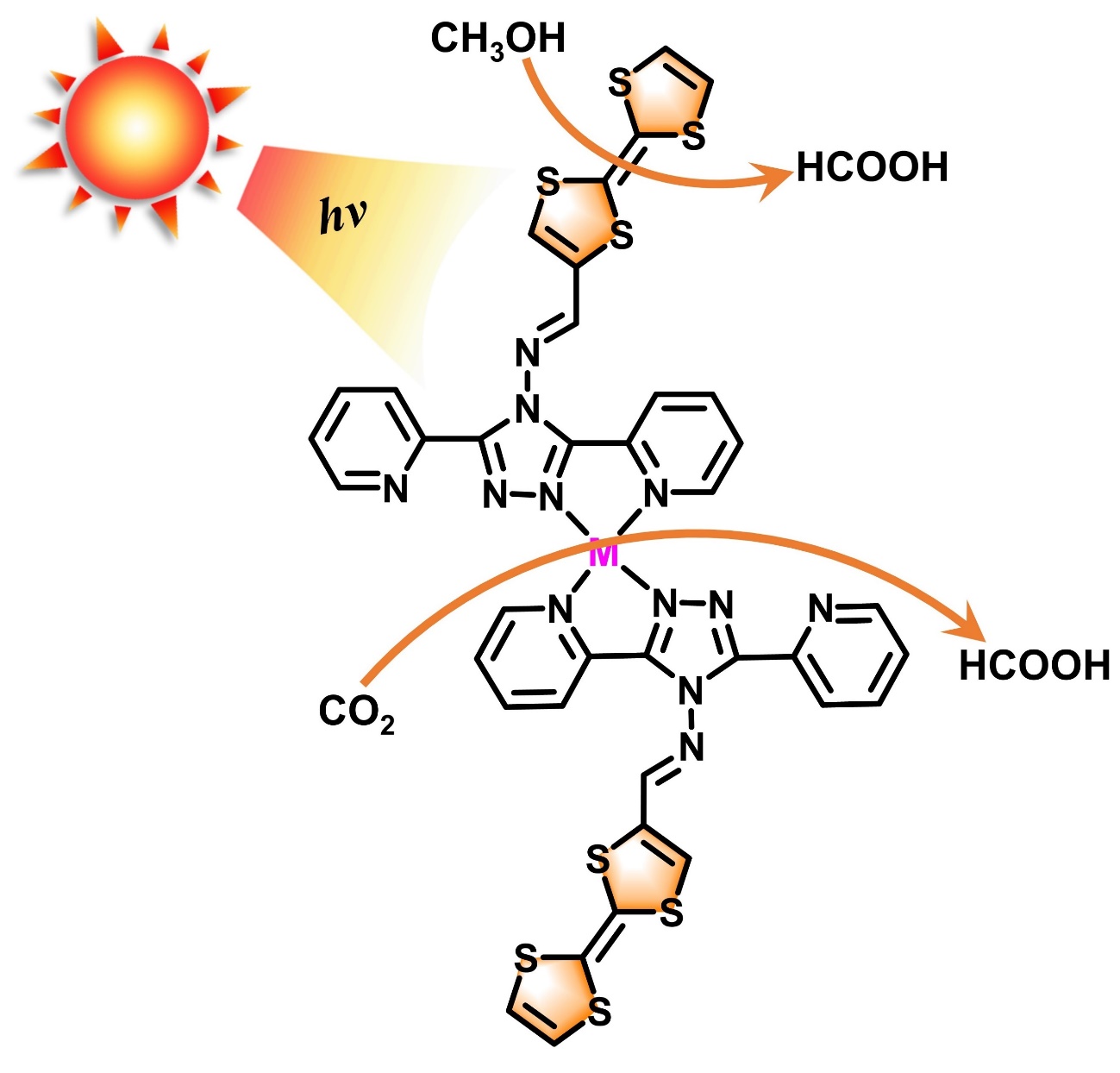


**Figure S63**. Schematic diagram of the photosynthesized HCOOH production through CO_2_ reduction and CH_3_OH oxidation over ML^2^.


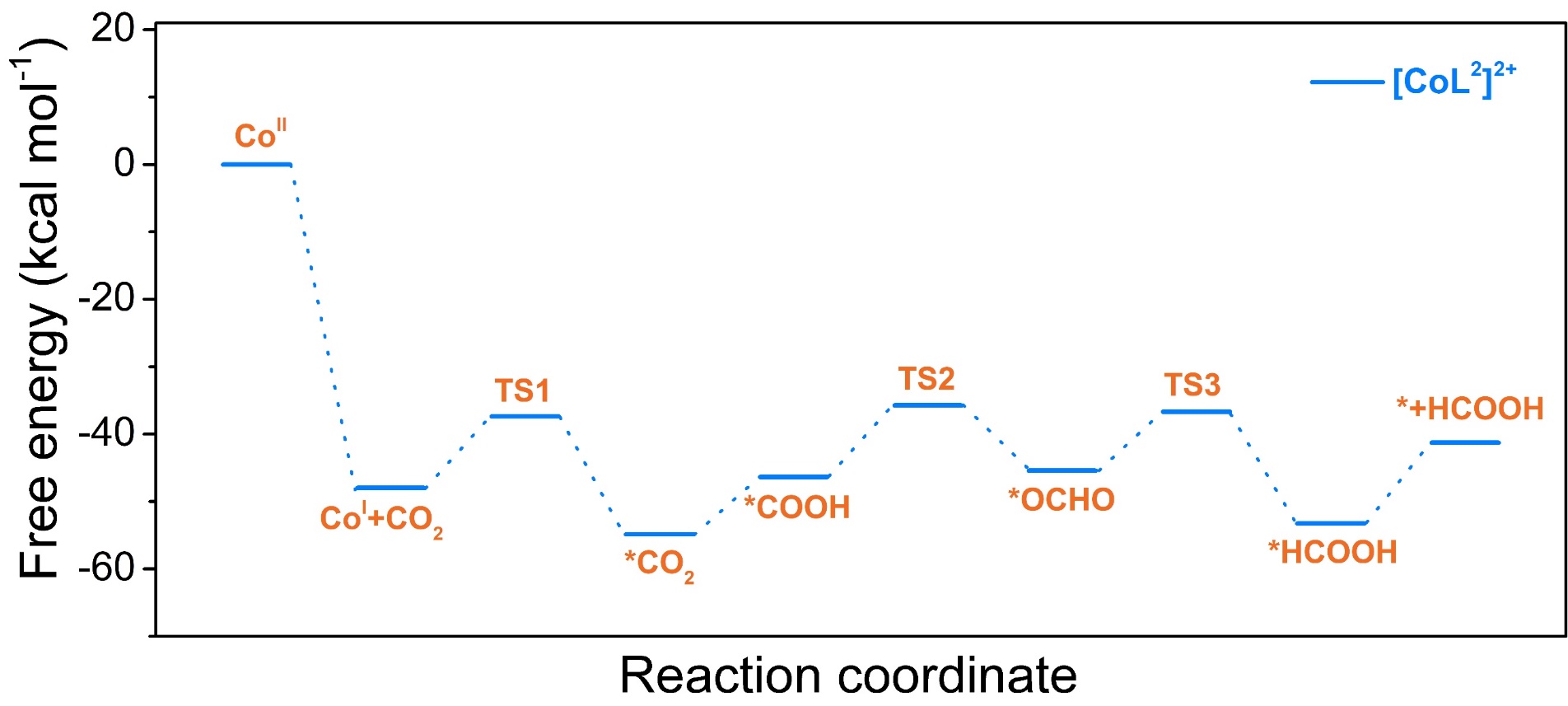


**Figure S64**. Energy diagram (ΔG(298 K) in kcal mol^-1^) for CO_2_ reduction catalyzed by [CoL^2^]^2+^.


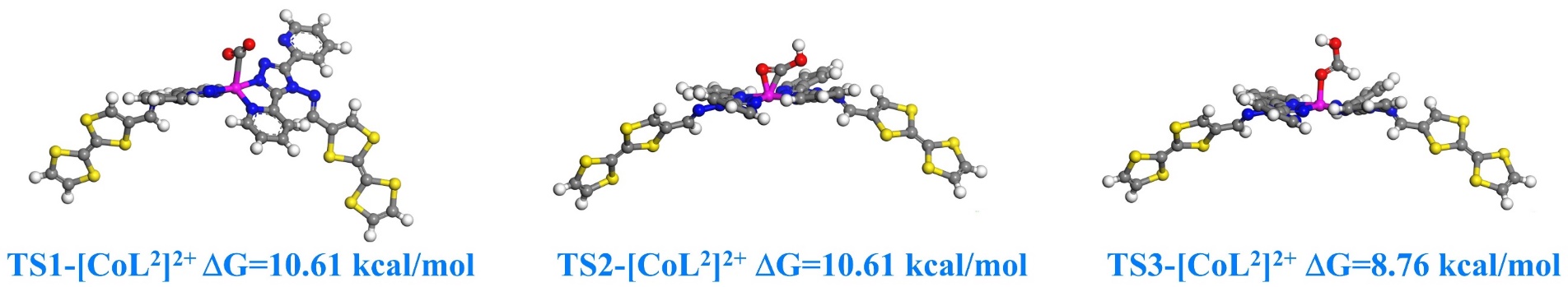


**Figure S65**. Energy barriers of the three transition states for [CoL^2^]^2+^. O red, Co fuchsia, Ni green, Cu orange, N blue, S yellow, C gray, H white.

**Table S1. Self-photosensitizing molecular photocatalytic systems without an additional PS/catalyst for CO_2_ reduction in the literature.**

| **Catalysts** | **[Catalysts]** | **Electron donors** | **Reaction conditions** | **Product (TON)** | **Light source** | **Reference** |
| --- | --- | --- | --- | --- | --- | --- |
| [CoL^2^]^2+^ | 1 µM | CH_3_OH | CH_3_OH/H_2_O  (*v/v* = 4:1) | HCOOH (855) | Xe lamp  (320 < λ < 800 nm) | **This work** |
| [NiL^2^]^2+^ |  |  |  | HCOOH (405) |  |  |
| [CuL^2^]^+^ |  |  |  | HCOOH (62) |  |  |
| [Ir(tpy)(ppy)Cl]^+^ | 0.5 mM | TEOA | CH_3_CN/TEOA  (*v/v* = 5:1) | CO (38) | Xe lamp  (410 ≤ λ ≤ 750 nm) | *Angew. Chem. Int. Ed.* **2013**, *52*, 988-992 |
| RuP-4 | 30 µM | BIH | DMA | CO (404) | Xe lamp  (λ ≥ 400 nm) | *Angew. Chem. Int. Ed.* **2024***, 63,* e202407448 |
| Fe-p-TMA | 2 µM | BIH+TEA | CH_3_CN | CO (33) | solar simulator, 1 sun  (λ > 420 nm) | *Chem. Commun.* **2017**, *53*, 2830-2833 |
| Mes-IrPCY2 | 20 µM | BIH | DMA/H_2_O (*v/v* = 9:1) | CO (2080) | Xe lamp  (λ ≥ 400 nm) | *J. Am. Chem. Soc.* **2020**, *142*, 10261-10266 |
| ZnP-phen=Re | 50 µM | BIH+PhOH | DMA | CO (900) | laser diode  (λ = 420 nm) | *J. Am. Chem. Soc.* **2020**, *142*, 705-709 |
| [Fe_2_Na_3_(PP)_6_][TBA]_3_ | 1 µM | BIH | DMF | CO (2625) | blue LED  (λ = 450 nm) | *J. Am. Chem. Soc.* **2022**, *144*, 4305-4309 |
| CuPP | 0.1 mM | BIH | DMF | CO (4.4) | white LED  (λ > 400 nm) | *Nat. Commun.* **2021**, *12*, 1835 |
| FeTPP | 50 µM | TEA | CH_3_CN | CO (17) | Xe lamp  (λ > 280 nm) | *ChemCatChem* **2014***, 6*, 3200-3207 |
| FeTDHPP | 50 µM | TEA | CH_3_CN | CO (28) | Xe lamp  (λ > 280 nm) |  |
| FFePorphyrin | 50 µM | TEA | CH_3_CN | CO (23) | Xe lamp  (λ > 280 nm) |  |
| {[Mn(bpy)(CO)_3_]}_2_ | 1 mM | PhOH | CH_3_CN | CO (0.275) | LED  (λ = 395 nm) | *Dalton Trans.* **2019***, 48*, 1226-1236 |
| Re(I)(bpy)(CO)_3_Cl | 0.5 mM | TEOA | DMF/TEOA (*v/v* = 5:1) | CO (15) | Hg lamp  (λ = 365 nm) | *J. Am. Chem. Soc.* **2008**, *130*, 2023-2031 |
| RuP | 40 µM | BIH | DMA/H_2_O (*v/v* = 39:1) | CO (58) | Xe lamp  (420 ≤ λ ≤ 750) | *J. Am. Chem. Soc.* **2018**, *140*, 16899-16903 |
| Re(N, S-NHC) | 0.5 mM | BIH | DMF/TEOA (*v/v* = 5:1) | CO (102) | white LED  (λ ≥ 400 nm) | *Dalton Trans.* **2016**, *45*, 14524-14529 |
|  | 0.5 mM | BIH | DMF/TEOA (*v/v* = 5:1) | CO (153) | white LED  (λ > 480 nm) |  |
| Re(Py-NHC-PhCF_3_) | 0.1 mM | BIH | CH_3_CN/TEA (*v/v* = 20:1) | CO (32) | Xe lamp  (λ > 300 nm) | *Inorg. Chem.* **2016**, *55*, 682-690 |
| Ir(9-anthryl-tpy) | 0.5 mM | TEOA | CH_3_CN/TEOA (*v/v* = 5:1) | CO (265) | blue LED  (λ = 450 nm) | *ACS Catal.* **2017**, *7*, 154-160 |
| Bis-[Ir(tpy)(mppy)Cl]PF_6_ | 0.17 mM | TEOA | CH_3_CN/TEOA (*v/v* = 5:1) | CO (81) | blue LED  (λ = 450 nm) | *Dalton Trans.* **2014**, *43*, 13259-13269 |
| Tri-[Ir(tpy)(bpy)L]PF_6_ | 0.34 mM | TEOA | CH_3_CN/TEOA (*v/v* = 5:1) | CO (60) | blue LED  (λ = 450 nm) | *Dalton Trans.* **2015**, *44*, 6466-6472 |
| [Os(bpy)(CO)_2_Cl_2_] | 0.5 mM | TEOA | DMF | CO (11.5) | laser diode  (λ = 405 nm) | *Chem. Eur. J.* **2011**, *17*, 4313-4322 |
| [Os(dmbpy)(CO)_2_Cl_2_] | 0.5 mM | TEOA | DMF | CO (19.5) | laser diode  (λ = 405 nm) |  |

**Table S2**. Energies of each system relative to its corresponding ground state energy (*ΔE=E(system) – E(ground)*).

|  | *ΔE* (eV) |
| --- | --- |
| [Co^I^L^2^]^+^ (S=0) | 0 |
| [Co^I^L^2^]^+^ (S=1) | 0.41 |

S = 0: low spin state; S = 1: high spin state

**Cartesian Coordinates**

| **[CoL^2^]^2+^** | | | |
| --- | --- | --- | --- |
| N | 5.01780000 | -1.64570000 | -0.83910000 |
| C | 5.42650000 | -0.68070000 | -0.00410000 |
| C | 6.82800000 | -0.52730000 | 0.28460000 |
| C | 7.87160000 | -1.13880000 | -0.35350000 |
| S | 9.56550000 | -0.81730000 | 0.18440000 |
| C | 9.08420000 | 0.41330000 | 1.47170000 |
| S | 7.26600000 | 0.60770000 | 1.67540000 |
| C | 9.98480000 | 1.09880000 | 2.20540000 |
| S | 11.81120000 | 0.89430000 | 2.01460000 |
| C | 12.22160000 | 2.16850000 | 3.24560000 |
| C | 11.21850000 | 2.79220000 | 3.89080000 |
| S | 9.50440000 | 2.32810000 | 3.49770000 |
| C | -2.42840000 | -3.53850000 | -1.67590000 |
| C | -3.73250000 | -2.99630000 | -1.66030000 |
| C | -3.90580000 | -1.61780000 | -1.50660000 |
| C | -2.76620000 | -0.78720000 | -1.35350000 |
| N | -1.47510000 | -1.33060000 | -1.41640000 |
| C | -1.32980000 | -2.67430000 | -1.56430000 |
| C | -2.72110000 | 0.63520000 | -1.21370000 |
| N | -3.66820000 | 1.66880000 | -1.15050000 |
| C | -2.93580000 | 2.87730000 | -1.33120000 |
| N | -1.63120000 | 2.58870000 | -1.44160000 |
| N | -1.48800000 | 1.22600000 | -1.35610000 |
| Co | -0.00040000 | 0.00140000 | -1.39190000 |
| C | 2.42750000 | 3.54150000 | -1.67400000 |
| C | 3.73160000 | 2.99930000 | -1.65980000 |
| C | 3.90500000 | 1.62060000 | -1.50750000 |
| C | 2.76550000 | 0.78990000 | -1.35430000 |
| N | 1.47430000 | 1.33350000 | -1.41570000 |
| C | 1.32900000 | 2.67720000 | -1.56240000 |
| C | 2.72050000 | -0.63260000 | -1.21530000 |
| N | 3.66760000 | -1.66620000 | -1.15310000 |
| C | 2.93510000 | -2.87460000 | -1.33380000 |
| N | 1.63040000 | -2.58600000 | -1.44280000 |
| N | 1.48720000 | -1.22330000 | -1.35630000 |
| C | -3.46760000 | 4.24600000 | -1.37430000 |
| C | -4.85120000 | 4.54050000 | -1.44150000 |
| C | -5.25370000 | 5.88600000 | -1.50090000 |
| C | -4.28100000 | 6.90060000 | -1.49360000 |
| C | -2.92460000 | 6.51730000 | -1.42610000 |
| N | -2.51810000 | 5.23300000 | -1.36750000 |
| C | 3.46690000 | -4.24320000 | -1.37790000 |
| C | 4.85040000 | -4.53760000 | -1.44710000 |
| C | 5.25290000 | -5.88310000 | -1.50750000 |
| C | 4.28030000 | -6.89780000 | -1.49920000 |
| C | 2.92390000 | -6.51460000 | -1.42970000 |
| N | 2.51740000 | -5.23030000 | -1.37010000 |
| N | -5.01820000 | 1.64820000 | -0.83560000 |
| C | -5.42640000 | 0.68270000 | -0.00090000 |
| C | -6.82780000 | 0.52890000 | 0.28820000 |
| C | -7.87160000 | 1.14000000 | -0.34970000 |
| S | -9.56530000 | 0.81810000 | 0.18870000 |
| C | -9.08350000 | -0.41450000 | 1.47380000 |
| S | -7.26520000 | -0.60580000 | 1.67950000 |
| C | -9.98370000 | -1.10330000 | 2.20480000 |
| S | -11.81020000 | -0.90180000 | 2.01220000 |
| C | -12.21970000 | -2.18200000 | 3.23730000 |
| C | -11.21630000 | -2.80530000 | 3.88230000 |
| S | -9.50270000 | -2.33470000 | 3.49500000 |
| H | 4.71730000 | -0.02980000 | 0.50990000 |
| H | 7.74810000 | -1.81040000 | -1.19300000 |
| H | 13.27670000 | 2.35740000 | 3.40120000 |
| H | 11.34310000 | 3.55960000 | 4.64490000 |
| H | -2.25450000 | -4.60270000 | -1.78920000 |
| H | -4.60060000 | -3.63970000 | -1.77480000 |
| H | -4.89740000 | -1.18540000 | -1.52810000 |
| H | -0.31050000 | -3.04650000 | -1.59270000 |
| H | 2.25360000 | 4.60590000 | -1.78620000 |
| H | 4.59970000 | 3.64270000 | -1.77440000 |
| H | 4.89660000 | 1.18820000 | -1.53020000 |
| H | 0.30970000 | 3.04950000 | -1.59010000 |
| H | -5.56800000 | 3.73110000 | -1.43500000 |
| H | -6.31090000 | 6.13310000 | -1.55070000 |
| H | -4.55320000 | 7.95090000 | -1.53710000 |
| H | -2.13530000 | 7.26560000 | -1.41710000 |
| H | 5.56710000 | -3.72810000 | -1.44130000 |
| H | 6.31010000 | -6.13010000 | -1.55890000 |
| H | 4.55250000 | -7.94810000 | -1.54340000 |
| H | 2.13480000 | -7.26300000 | -1.41990000 |
| H | -4.71690000 | 0.03150000 | 0.51230000 |
| H | -7.74850000 | 1.81160000 | -1.18930000 |
| H | -13.27460000 | -2.37440000 | 3.38990000 |
| H | -11.34040000 | -3.57580000 | 4.63330000 |

| **[CoL^2^]^2+^+CO_2_** | | | |
| --- | --- | --- | --- |
| N | -5.07180000 | 1.46710000 | -0.82650000 |
| C | -5.45960000 | 0.59930000 | 0.10580000 |
| C | -6.86050000 | 0.32900000 | 0.30280000 |
| C | -7.88640000 | 0.78660000 | -0.47590000 |
| S | -9.58230000 | 0.33980000 | -0.07220000 |
| C | -9.13180000 | -0.58840000 | 1.46230000 |
| S | -7.31480000 | -0.75410000 | 1.72660000 |
| C | -10.05100000 | -1.10230000 | 2.30650000 |
| S | -11.87370000 | -0.94310000 | 2.04020000 |
| C | -12.31190000 | -1.75100000 | 3.61100000 |
| C | -11.32390000 | -2.22390000 | 4.39360000 |
| S | -9.60090000 | -2.03000000 | 3.84040000 |
| C | 2.35150000 | 3.58280000 | -1.42460000 |
| C | 3.65830000 | 3.06550000 | -1.45920000 |
| C | 3.84970000 | 1.68230000 | -1.32120000 |
| C | 2.72500000 | 0.85440000 | -1.14890000 |
| N | 1.43550000 | 1.36240000 | -1.15620000 |
| C | 1.26570000 | 2.70150000 | -1.28540000 |
| C | 2.70640000 | -0.58300000 | -1.02830000 |
| N | 3.68270000 | -1.57330000 | -0.99860000 |
| C | 2.99750000 | -2.80170000 | -1.09120000 |
| N | 1.67840000 | -2.56420000 | -1.13590000 |
| N | 1.49990000 | -1.19420000 | -1.08970000 |
| Co | 0.00000000 | -0.00020000 | -1.20950000 |
| C | -2.35150000 | -3.58320000 | -1.42390000 |
| C | -3.65830000 | -3.06600000 | -1.45860000 |
| C | -3.84970000 | -1.68270000 | -1.32100000 |
| C | -2.72500000 | -0.85480000 | -1.14880000 |
| N | -1.43550000 | -1.36280000 | -1.15600000 |
| C | -1.26560000 | -2.70190000 | -1.28480000 |
| C | -2.70640000 | 0.58270000 | -1.02840000 |
| N | -3.68260000 | 1.57300000 | -0.99890000 |
| C | -2.99740000 | 2.80130000 | -1.09160000 |
| N | -1.67840000 | 2.56380000 | -1.13630000 |
| N | -1.49990000 | 1.19380000 | -1.08990000 |
| C | 3.59410000 | -4.14530000 | -1.12050000 |
| C | 4.94950000 | -4.36910000 | -1.45830000 |
| C | 5.42280000 | -5.69180000 | -1.50440000 |
| C | 4.54190000 | -6.74910000 | -1.21440000 |
| C | 3.20640000 | -6.43280000 | -0.88670000 |
| N | 2.73410000 | -5.16900000 | -0.83780000 |
| C | -3.59400000 | 4.14500000 | -1.12110000 |
| C | -4.94940000 | 4.36890000 | -1.45880000 |
| C | -5.42260000 | 5.69160000 | -1.50510000 |
| C | -4.54160000 | 6.74880000 | -1.21530000 |
| C | -3.20610000 | 6.43250000 | -0.88770000 |
| N | -2.73380000 | 5.16860000 | -0.83860000 |
| N | 5.07180000 | -1.46740000 | -0.82610000 |
| C | 5.45960000 | -0.59940000 | 0.10600000 |
| C | 6.86050000 | -0.32910000 | 0.30310000 |
| C | 7.88640000 | -0.78680000 | -0.47550000 |
| S | 9.58220000 | -0.34000000 | -0.07190000 |
| C | 9.13180000 | 0.58870000 | 1.46240000 |
| S | 7.31470000 | 0.75420000 | 1.72680000 |
| C | 10.05090000 | 1.10290000 | 2.30630000 |
| S | 11.87360000 | 0.94390000 | 2.04000000 |
| C | 12.31180000 | 1.75260000 | 3.61040000 |
| C | 11.32380000 | 2.22560000 | 4.39290000 |
| S | 9.60070000 | 2.03110000 | 3.84000000 |
| H | -4.73790000 | 0.08500000 | 0.74520000 |
| H | -7.73380000 | 1.39790000 | -1.35650000 |
| H | -13.36980000 | -1.81680000 | 3.83510000 |
| H | -11.46750000 | -2.72710000 | 5.34210000 |
| H | 2.15800000 | 4.64540000 | -1.52480000 |
| H | 4.51360000 | 3.72060000 | -1.59820000 |
| H | 4.84360000 | 1.25900000 | -1.37980000 |
| H | 0.24110000 | 3.05490000 | -1.28400000 |
| H | -2.15800000 | -4.64590000 | -1.52380000 |
| H | -4.51360000 | -3.72110000 | -1.59760000 |
| H | -4.84350000 | -1.25940000 | -1.37980000 |
| H | -0.24110000 | -3.05530000 | -1.28330000 |
| H | 5.59640000 | -3.52750000 | -1.66760000 |
| H | 6.45940000 | -5.88930000 | -1.76340000 |
| H | 4.86950000 | -7.78400000 | -1.23820000 |
| H | 2.48970000 | -7.21640000 | -0.65360000 |
| H | -5.59640000 | 3.52730000 | -1.66800000 |
| H | -6.45920000 | 5.88910000 | -1.76400000 |
| H | -4.86920000 | 7.78380000 | -1.23920000 |
| H | -2.48940000 | 7.21610000 | -0.65480000 |
| H | 4.73780000 | -0.08490000 | 0.74530000 |
| H | 7.73380000 | -1.39820000 | -1.35610000 |
| H | 13.36970000 | 1.81880000 | 3.83440000 |
| H | 11.46730000 | 2.72920000 | 5.34120000 |
| C | -0.00010000 | -0.00040000 | -3.35570000 |
| O | -1.17900000 | -0.05980000 | -3.73410000 |
| O | 1.17880000 | 0.05890000 | -3.73410000 |

| **[CoL^2^]^2+^-COOH** | | | |
| --- | --- | --- | --- |
| N | -5.08380000 | 1.41570000 | -0.74530000 |
| C | -5.49670000 | 0.45340000 | 0.05710000 |
| C | -6.91020000 | 0.31430000 | 0.34760000 |
| C | -7.92350000 | 1.04400000 | -0.19400000 |
| S | -9.62560000 | 0.71310000 | 0.29410000 |
| C | -9.17150000 | -0.56640000 | 1.54980000 |
| S | -7.37890000 | -0.99760000 | 1.55580000 |
| C | -10.07180000 | -1.13570000 | 2.37850000 |
| S | -11.87060000 | -0.70890000 | 2.36760000 |
| C | -12.26850000 | -1.76170000 | 3.79800000 |
| C | -11.28890000 | -2.50350000 | 4.34800000 |
| S | -9.61760000 | -2.41490000 | 3.63280000 |
| C | 2.23080000 | 3.64780000 | -1.32480000 |
| C | 3.55860000 | 3.16360000 | -1.42620000 |
| C | 3.78680000 | 1.79230000 | -1.32830000 |
| C | 2.69030000 | 0.92030000 | -1.10880000 |
| N | 1.37610000 | 1.39490000 | -1.07510000 |
| C | 1.17810000 | 2.74270000 | -1.18000000 |
| C | 2.71580000 | -0.49730000 | -0.98220000 |
| N | 3.71500000 | -1.48140000 | -1.00700000 |
| C | 3.03960000 | -2.71750000 | -1.12390000 |
| N | 1.72590000 | -2.51120000 | -1.11990000 |
| N | 1.51110000 | -1.13870000 | -1.00760000 |
| Co | -0.00160000 | 0.00130000 | -1.22830000 |
| C | -2.23020000 | -3.63910000 | -1.42710000 |
| C | -3.55990000 | -3.15560000 | -1.51410000 |
| C | -3.79100000 | -1.78790000 | -1.38160000 |
| C | -2.69590000 | -0.91820000 | -1.14310000 |
| N | -1.37940000 | -1.39060000 | -1.12490000 |
| C | -1.17960000 | -2.73580000 | -1.26100000 |
| C | -2.72220000 | 0.49550000 | -0.99710000 |
| N | -3.72060000 | 1.48170000 | -1.02010000 |
| C | -3.04240000 | 2.71720000 | -1.13750000 |
| N | -1.72930000 | 2.50860000 | -1.14000000 |
| N | -1.51540000 | 1.13580000 | -1.02920000 |
| C | 3.66630000 | -4.04590000 | -1.22560000 |
| C | 4.96530000 | -4.22490000 | -1.75500000 |
| C | 5.46940000 | -5.53060000 | -1.87750000 |
| C | 4.67320000 | -6.61590000 | -1.46810000 |
| C | 3.39030000 | -6.34390000 | -0.94950000 |
| N | 2.88960000 | -5.09470000 | -0.82690000 |
| C | -3.66490000 | 4.04810000 | -1.23250000 |
| C | -4.97340000 | 4.23300000 | -1.73610000 |
| C | -5.47260000 | 5.54120000 | -1.85290000 |
| C | -4.66240000 | 6.62350000 | -1.46370000 |
| C | -3.37090000 | 6.34580000 | -0.96990000 |
| N | -2.87480000 | 5.09440000 | -0.85320000 |
| N | 5.08210000 | -1.41140000 | -0.74630000 |
| C | 5.49440000 | -0.46210000 | 0.07090000 |
| C | 6.91010000 | -0.31170000 | 0.34570000 |
| C | 7.92440000 | -1.00750000 | -0.23680000 |
| S | 9.62870000 | -0.67050000 | 0.23670000 |
| C | 9.17780000 | 0.56300000 | 1.53900000 |
| S | 7.37890000 | 0.96440000 | 1.59130000 |
| C | 10.08440000 | 1.12160000 | 2.36800000 |
| S | 11.88920000 | 0.72480000 | 2.31130000 |
| C | 12.29820000 | 1.74460000 | 3.76240000 |
| C | 11.31750000 | 2.45330000 | 4.35240000 |
| S | 9.63360000 | 2.35480000 | 3.66880000 |
| H | -4.80570000 | -0.23730000 | 0.54340000 |
| H | -7.76470000 | 1.82320000 | -0.92870000 |
| H | -13.29820000 | -1.74160000 | 4.13420000 |
| H | -11.41220000 | -3.16960000 | 5.19350000 |
| H | 2.00860000 | 4.70770000 | -1.38760000 |
| H | 4.38770000 | 3.84520000 | -1.59430000 |
| H | 4.78440000 | 1.39310000 | -1.46050000 |
| H | 0.14640000 | 3.07040000 | -1.16010000 |
| H | -2.00590000 | -4.69690000 | -1.51360000 |
| H | -4.38780000 | -3.83520000 | -1.69550000 |
| H | -4.79020000 | -1.38810000 | -1.49870000 |
| H | -0.14780000 | -3.06310000 | -1.24090000 |
| H | 5.54910000 | -3.36350000 | -2.05090000 |
| H | 6.46270000 | -5.69550000 | -2.28600000 |
| H | 5.02720000 | -7.63970000 | -1.54420000 |
| H | 2.74020000 | -7.14980000 | -0.61810000 |
| H | -5.56860000 | 3.37370000 | -2.01500000 |
| H | -6.47350000 | 5.71020000 | -2.24100000 |
| H | -5.01230000 | 7.64890000 | -1.53600000 |
| H | -2.70990000 | 7.14920000 | -0.65430000 |
| H | 4.80190000 | 0.21110000 | 0.57920000 |
| H | 7.76410000 | -1.76100000 | -0.99770000 |
| H | 13.33470000 | 1.73330000 | 4.07710000 |
| H | 11.44650000 | 3.09780000 | 5.21360000 |
| C | 0.08410000 | 0.00950000 | -3.14660000 |
| O | -1.17540000 | 0.08180000 | -3.77280000 |
| O | 1.11280000 | -0.04150000 | -3.84030000 |
| H | -1.06270000 | 0.07340000 | -4.75650000 |

| **[CoL^2^]^2+^-HCOOH** | | | |
| --- | --- | --- | --- |
| N | -4.99570000 | 1.68290000 | -0.51080000 |
| C | -5.37140000 | 0.69560000 | 0.32060000 |
| C | -6.76430000 | 0.48990000 | 0.59670000 |
| C | -7.82100000 | 1.08750000 | -0.03770000 |
| S | -9.51060000 | 0.69950000 | 0.47530000 |
| C | -9.00220000 | -0.56910000 | 1.71750000 |
| S | -7.17980000 | -0.69390000 | 1.95370000 |
| C | -9.88690000 | -1.32580000 | 2.40070000 |
| S | -11.71930000 | -1.19260000 | 2.17980000 |
| C | -12.09420000 | -2.55610000 | 3.32510000 |
| C | -11.07700000 | -3.16460000 | 3.96390000 |
| S | -9.37830000 | -2.59260000 | 3.64950000 |
| C | 2.46960000 | 3.55920000 | -0.90550000 |
| C | 3.76750000 | 2.99970000 | -0.94700000 |
| C | 3.92040000 | 1.61120000 | -0.98920000 |
| C | 2.76590000 | 0.78580000 | -0.96850000 |
| N | 1.48520000 | 1.35060000 | -0.97970000 |
| C | 1.35980000 | 2.70270000 | -0.93990000 |
| C | 2.70200000 | -0.64120000 | -1.00780000 |
| N | 3.64370000 | -1.67850000 | -1.05730000 |
| C | 2.90980000 | -2.85320000 | -1.37940000 |
| N | 1.60590000 | -2.54610000 | -1.46580000 |
| N | 1.46950000 | -1.20310000 | -1.22180000 |
| C | -2.46970000 | -3.44510000 | -1.66600000 |
| C | -3.76600000 | -2.89860000 | -1.55700000 |
| C | -3.92160000 | -1.53030000 | -1.30650000 |
| C | -2.76760000 | -0.72350000 | -1.14880000 |
| N | -1.48820000 | -1.26860000 | -1.29390000 |
| C | -1.35840000 | -2.59610000 | -1.54140000 |
| C | -2.70570000 | 0.69110000 | -0.93280000 |
| N | -3.65200000 | 1.72360000 | -0.83970000 |
| C | -2.92760000 | 2.93240000 | -1.02550000 |
| N | -1.61970000 | 2.64340000 | -1.16100000 |
| N | -1.47670000 | 1.28080000 | -1.07730000 |
| C | 3.44130000 | -4.20780000 | -1.56670000 |
| C | 4.82790000 | -4.49140000 | -1.63150000 |
| C | 5.23670000 | -5.82150000 | -1.83030000 |
| C | 4.26690000 | -6.83110000 | -1.96200000 |
| C | 2.90740000 | -6.45820000 | -1.88700000 |
| N | 2.49380000 | -5.18910000 | -1.69470000 |
| C | -3.46320000 | 4.29620000 | -1.05300000 |
| C | -4.84970000 | 4.58520000 | -1.00240000 |
| C | -5.26050000 | 5.92880000 | -1.04330000 |
| C | -4.29430000 | 6.94610000 | -1.13740000 |
| C | -2.93500000 | 6.56730000 | -1.18700000 |
| N | -2.51840000 | 5.28550000 | -1.14620000 |
| N | 4.99310000 | -1.68700000 | -0.73850000 |
| C | 5.37930000 | -0.84110000 | 0.22720000 |
| C | 6.77700000 | -0.68570000 | 0.52220000 |
| C | 7.82550000 | -1.18820000 | -0.19960000 |
| S | 9.51980000 | -0.89020000 | 0.35540000 |
| C | 9.02480000 | 0.20950000 | 1.75450000 |
| S | 7.20640000 | 0.27930000 | 2.03860000 |
| C | 9.91560000 | 0.88720000 | 2.50890000 |
| S | 11.74410000 | 0.80400000 | 2.23940000 |
| C | 12.12500000 | 2.05170000 | 3.50850000 |
| C | 11.11510000 | 2.56550000 | 4.23580000 |
| S | 9.42030000 | 1.98600000 | 3.91210000 |
| H | -4.63730000 | 0.06450000 | 0.82520000 |
| H | -7.70400000 | 1.78540000 | -0.85670000 |
| H | -13.14150000 | -2.80800000 | 3.44140000 |
| H | -11.18430000 | -3.97890000 | 4.67040000 |
| H | 2.31170000 | 4.63140000 | -0.86850000 |
| H | 4.64560000 | 3.63970000 | -0.95470000 |
| H | 4.90580000 | 1.16970000 | -1.05820000 |
| H | 0.34530000 | 3.08790000 | -0.95040000 |
| H | -2.30990000 | -4.50010000 | -1.85930000 |
| H | -4.64390000 | -3.52820000 | -1.67380000 |
| H | -4.90860000 | -1.09020000 | -1.25510000 |
| H | -0.34270000 | -2.96590000 | -1.63850000 |
| H | 5.54040000 | -3.68600000 | -1.51570000 |
| H | 6.29580000 | -6.06080000 | -1.87930000 |
| H | 4.54330000 | -7.87000000 | -2.11550000 |
| H | 2.12110000 | -7.20360000 | -1.98240000 |
| H | -5.55790000 | 3.77150000 | -0.92370000 |
| H | -6.31880000 | 6.17250000 | -1.00110000 |
| H | -4.57300000 | 7.99500000 | -1.17140000 |
| H | -2.15190000 | 7.31850000 | -1.26120000 |
| H | 4.65480000 | -0.28600000 | 0.82600000 |
| H | 7.69990000 | -1.75920000 | -1.11050000 |
| H | 13.16990000 | 2.31450000 | 3.62160000 |
| H | 11.22640000 | 3.30320000 | 5.02130000 |
| O | 0.07260000 | 0.38750000 | -3.45570000 |
| C | -0.17380000 | 1.49760000 | -3.95770000 |
| O | -0.06470000 | 1.75720000 | -5.29070000 |
| Co | 0.00130000 | 0.04650000 | -1.23440000 |
| H | 0.23140000 | 0.96860000 | -5.80410000 |
| H | -0.50510000 | 2.37680000 | -3.40100000 |

| **[CoL^2^]^2+^-OCOH** | | | |
| --- | --- | --- | --- |
| N | 5.03020000 | -1.41000000 | -0.67330000 |
| C | 5.47400000 | -0.49260000 | 0.15680000 |
| C | 6.90180000 | -0.32530000 | 0.35840000 |
| C | 7.88660000 | -0.97050000 | -0.32280000 |
| S | 9.60890000 | -0.63050000 | 0.06170000 |
| C | 9.22290000 | 0.52680000 | 1.45360000 |
| S | 7.42400000 | 0.89100000 | 1.63990000 |
| C | 10.17190000 | 1.05740000 | 2.25280000 |
| S | 11.97450000 | 0.69720000 | 2.06240000 |
| C | 12.46000000 | 1.65010000 | 3.53520000 |
| C | 11.50810000 | 2.30870000 | 4.22240000 |
| S | 9.78550000 | 2.21120000 | 3.64300000 |
| C | -2.31220000 | -3.55390000 | -1.50670000 |
| C | -3.63130000 | -3.05340000 | -1.61740000 |
| C | -3.85320000 | -1.68640000 | -1.44560000 |
| C | -2.75600000 | -0.84070000 | -1.14640000 |
| N | -1.45050000 | -1.33030000 | -1.08420000 |
| C | -1.25800000 | -2.66820000 | -1.26600000 |
| C | -2.76890000 | 0.57380000 | -0.95790000 |
| N | -3.75950000 | 1.56730000 | -0.93950000 |
| C | -3.07220000 | 2.80050000 | -0.97170000 |
| N | -1.75940000 | 2.58170000 | -0.96350000 |
| N | -1.55700000 | 1.19970000 | -0.92990000 |
| C | 2.23250000 | 3.65010000 | -1.31810000 |
| C | 3.55090000 | 3.14830000 | -1.38620000 |
| C | 3.76200000 | 1.77640000 | -1.22710000 |
| C | 2.65410000 | 0.93280000 | -0.99140000 |
| N | 1.35400000 | 1.42270000 | -0.97280000 |
| C | 1.16710000 | 2.76220000 | -1.13420000 |
| C | 2.65850000 | -0.49220000 | -0.82590000 |
| N | 3.64410000 | -1.48040000 | -0.84980000 |
| C | 2.96350000 | -2.71010000 | -0.92220000 |
| N | 1.65050000 | -2.49440000 | -0.88750000 |
| N | 1.45400000 | -1.11600000 | -0.81120000 |
| C | -3.68690000 | 4.13920000 | -1.00450000 |
| C | -4.97810000 | 4.36050000 | -1.53680000 |
| C | -5.46790000 | 5.67630000 | -1.59260000 |
| C | -4.66560000 | 6.72890000 | -1.11550000 |
| C | -3.39190000 | 6.41530000 | -0.59800000 |
| N | -2.90500000 | 5.15590000 | -0.53960000 |
| C | 3.59430000 | -4.03780000 | -1.01560000 |
| C | 4.80370000 | -4.23080000 | -1.72090000 |
| C | 5.31720000 | -5.53340000 | -1.83240000 |
| C | 4.61720000 | -6.59940000 | -1.23700000 |
| C | 3.41970000 | -6.31300000 | -0.55070000 |
| N | 2.91100000 | -5.06480000 | -0.43640000 |
| N | -5.13380000 | 1.49140000 | -0.70550000 |
| C | -5.55960000 | 0.50550000 | 0.05830000 |
| C | -6.97970000 | 0.34320000 | 0.30480000 |
| C | -7.98400000 | 1.06960000 | -0.25670000 |
| S | -9.69480000 | 0.71360000 | 0.17340000 |
| C | -9.26560000 | -0.58770000 | 1.41650000 |
| S | -7.46730000 | -0.99230000 | 1.47810000 |
| C | -10.18590000 | -1.18850000 | 2.19960000 |
| S | -11.98930000 | -0.78840000 | 2.13360000 |
| C | -12.42390000 | -1.89030000 | 3.51550000 |
| C | -11.45280000 | -2.62950000 | 4.08370000 |
| S | -9.75610000 | -2.48840000 | 3.44070000 |
| H | 4.80040000 | 0.15130000 | 0.72700000 |
| H | 7.68770000 | -1.68330000 | -1.11360000 |
| H | 13.51490000 | 1.64320000 | 3.78180000 |
| H | 11.68170000 | 2.91140000 | 5.10560000 |
| H | -2.09630000 | -4.61010000 | -1.62810000 |
| H | -4.46030000 | -3.71680000 | -1.84800000 |
| H | -4.84450000 | -1.27180000 | -1.58030000 |
| H | -0.23130000 | -3.00670000 | -1.21040000 |
| H | 2.02320000 | 4.70870000 | -1.43000000 |
| H | 4.39010000 | 3.81170000 | -1.57480000 |
| H | 4.75630000 | 1.36290000 | -1.33430000 |
| H | 0.13910000 | 3.10150000 | -1.11710000 |
| H | -5.56820000 | 3.52300000 | -1.88430000 |
| H | -6.45490000 | 5.87390000 | -2.00170000 |
| H | -5.00850000 | 7.75900000 | -1.13940000 |
| H | -2.73790000 | 7.19450000 | -0.21490000 |
| H | 5.31110000 | -3.38420000 | -2.16570000 |
| H | 6.24020000 | -5.71200000 | -2.37710000 |
| H | 4.98060000 | -7.62080000 | -1.29980000 |
| H | 2.84630000 | -7.10400000 | -0.07390000 |
| H | -4.87570000 | -0.19360000 | 0.54290000 |
| H | -7.81120000 | 1.86070000 | -0.97560000 |
| H | -13.46650000 | -1.89880000 | 3.80980000 |
| H | -11.59670000 | -3.32200000 | 4.90430000 |
| O | 0.04080000 | 0.01870000 | -3.10690000 |
| C | 1.18120000 | -0.21470000 | -3.70160000 |
| O | 0.99420000 | -0.20000000 | -5.08950000 |
| Co | -0.04360000 | 0.04350000 | -1.13750000 |
| H | 0.03990000 | -0.01680000 | -5.32570000 |

| **[CoL^2^]^2+^-TS1** | | | |
| --- | --- | --- | --- |
| N | 5.29140000 | 1.30550000 | -0.73280000 |
| C | 5.30950000 | -0.00900000 | -0.39220000 |
| C | 6.56720000 | -0.64890000 | -0.00710000 |
| C | 7.76910000 | 0.01490000 | 0.07530000 |
| S | 9.19910000 | -0.85990000 | 0.54650000 |
| C | 8.34200000 | -2.44460000 | 0.73660000 |
| S | 6.56790000 | -2.39630000 | 0.38950000 |
| C | 8.99460000 | -3.58000000 | 1.11350000 |
| S | 10.76030000 | -3.62100000 | 1.45730000 |
| C | 10.76820000 | -5.35440000 | 1.84580000 |
| C | 9.60780000 | -6.03260000 | 1.77720000 |
| S | 8.13980000 | -5.15180000 | 1.30200000 |
| C | -1.36090000 | 0.25380000 | -3.53970000 |
| C | -2.77090000 | 0.09670000 | -3.29000000 |
| C | -3.28660000 | 0.49720000 | -2.06490000 |
| C | -2.42260000 | 1.03650000 | -1.06500000 |
| N | -0.99720000 | 1.19520000 | -1.28900000 |
| C | -0.53910000 | 0.78910000 | -2.57020000 |
| C | -2.75280000 | 1.57070000 | 0.22660000 |
| N | -3.90670000 | 1.70890000 | 1.03500000 |
| C | -3.49300000 | 2.47140000 | 2.18690000 |
| N | -2.15100000 | 2.77000000 | 2.14740000 |
| N | -1.69860000 | 2.19250000 | 0.89610000 |
| Co | -0.01980000 | 2.02130000 | -0.00700000 |
| C | 1.34790000 | 0.03530000 | 3.49680000 |
| C | 2.76510000 | -0.05520000 | 3.25430000 |
| C | 3.28470000 | 0.41970000 | 2.06020000 |
| C | 2.41240000 | 0.97790000 | 1.06970000 |
| N | 0.98710000 | 1.08000000 | 1.30190000 |
| C | 0.52370000 | 0.59900000 | 2.54020000 |
| C | 2.72800000 | 1.55410000 | -0.20330000 |
| N | 3.88240000 | 1.70870000 | -1.01570000 |
| C | 3.45890000 | 2.45610000 | -2.17410000 |
| N | 2.11540000 | 2.73510000 | -2.13930000 |
| N | 1.66400000 | 2.16980000 | -0.88210000 |
| C | -4.38720000 | 2.87270000 | 3.32480000 |
| C | -5.79450000 | 2.72390000 | 3.24980000 |
| C | -6.57710000 | 3.13570000 | 4.34430000 |
| C | -5.92990000 | 3.67980000 | 5.46980000 |
| C | -4.51940000 | 3.79150000 | 5.46190000 |
| N | -3.69160000 | 3.40510000 | 4.42060000 |
| C | 4.34750000 | 2.86320000 | -3.31430000 |
| C | 5.75760000 | 2.74370000 | -3.23540000 |
| C | 6.53460000 | 3.16060000 | -4.33170000 |
| C | 5.87990000 | 3.68130000 | -5.46380000 |
| C | 4.46760000 | 3.76400000 | -5.46030000 |
| N | 3.64480000 | 3.37060000 | -4.41770000 |
| N | -5.31300000 | 1.29890000 | 0.73540000 |
| C | -5.31650000 | -0.01880000 | 0.40280000 |
| C | -6.56230000 | -0.67150000 | 0.00540000 |
| C | -7.76950000 | -0.01710000 | -0.09240000 |
| S | -9.18540000 | -0.90390000 | -0.57690000 |
| C | -8.31460000 | -2.48200000 | -0.75450000 |
| S | -6.54510000 | -2.41990000 | -0.38610000 |
| C | -8.95470000 | -3.62320000 | -1.13790000 |
| S | -10.71490000 | -3.67830000 | -1.50300000 |
| C | -10.70460000 | -5.41200000 | -1.89000000 |
| C | -9.53980000 | -6.08090000 | -1.80620000 |
| S | -8.08520000 | -5.18750000 | -1.31340000 |
| H | 4.39170000 | -0.62760000 | -0.39010000 |
| H | 7.85980000 | 1.08740000 | -0.14300000 |
| H | 11.72650000 | -5.80520000 | 2.12790000 |
| H | 9.50860000 | -7.10180000 | 1.99670000 |
| H | -0.93120000 | -0.03760000 | -4.50630000 |
| H | -3.42720000 | -0.31550000 | -4.06780000 |
| H | -4.36000000 | 0.42550000 | -1.84920000 |
| H | 0.53400000 | 0.95670000 | -2.74220000 |
| H | 0.91850000 | -0.32070000 | 4.44090000 |
| H | 3.42670000 | -0.48100000 | 4.02050000 |
| H | 4.36330000 | 0.39570000 | 1.86210000 |
| H | -0.55760000 | 0.72260000 | 2.70130000 |
| H | -6.23070000 | 2.28890000 | 2.33820000 |
| H | -7.67120000 | 3.03500000 | 4.31740000 |
| H | -6.50240000 | 4.01440000 | 6.34580000 |
| H | -3.99660000 | 4.21400000 | 6.33680000 |
| H | 6.19890000 | 2.32400000 | -2.31910000 |
| H | 7.63040000 | 3.08180000 | -4.30110000 |
| H | 6.44780000 | 4.01930000 | -6.34150000 |
| H | 3.93890000 | 4.16730000 | -6.34060000 |
| H | -4.39310000 | -0.62940000 | 0.41630000 |
| H | -7.87150000 | 1.05520000 | 0.12240000 |
| H | -11.65590000 | -5.87030000 | -2.18350000 |
| H | -9.42890000 | -7.14940000 | -2.02330000 |
| C | -0.02910000 | 4.54050000 | -0.00350000 |
| O | 0.48650000 | 3.92080000 | 0.98640000 |
| O | -0.41190000 | 5.46650000 | -0.70860000 |

| **[CoL^2^]^2+^-TS2** | | | |
| --- | --- | --- | --- |
| N | -5.16500000 | 1.36860000 | -0.77990000 |
| C | -5.57790000 | 0.40630000 | 0.02250000 |
| C | -6.99140000 | 0.26730000 | 0.31310000 |
| C | -8.00470000 | 0.99690000 | -0.22850000 |
| S | -9.70680000 | 0.66610000 | 0.25950000 |
| C | -9.25270000 | -0.61350000 | 1.51520000 |
| S | -7.46010000 | -1.04470000 | 1.52130000 |
| C | -10.15300000 | -1.18280000 | 2.34390000 |
| S | -11.95180000 | -0.75590000 | 2.33300000 |
| C | -12.34970000 | -1.80880000 | 3.76350000 |
| C | -11.37010000 | -2.55060000 | 4.31350000 |
| S | -9.69880000 | -2.46200000 | 3.59820000 |
| C | 2.14960000 | 3.60080000 | -1.35940000 |
| C | 3.47740000 | 3.11650000 | -1.46080000 |
| C | 3.70570000 | 1.74530000 | -1.36280000 |
| C | 2.60910000 | 0.87330000 | -1.14340000 |
| N | 1.29490000 | 1.34790000 | -1.10970000 |
| C | 1.09690000 | 2.69570000 | -1.21460000 |
| C | 2.63460000 | -0.54430000 | -1.01680000 |
| N | 3.63380000 | -1.52840000 | -1.04160000 |
| C | 2.95840000 | -2.76450000 | -1.15840000 |
| N | 1.64480000 | -2.55830000 | -1.15450000 |
| N | 1.42990000 | -1.18580000 | -1.04220000 |
| C | -2.31140000 | -3.68610000 | -1.46170000 |
| C | -3.64110000 | -3.20260000 | -1.54860000 |
| C | -3.87220000 | -1.83490000 | -1.41610000 |
| C | -2.77710000 | -0.96530000 | -1.17770000 |
| N | -1.46060000 | -1.43770000 | -1.15950000 |
| C | -1.26080000 | -2.78290000 | -1.29560000 |
| C | -2.80330000 | 0.44840000 | -1.03160000 |
| N | -3.80170000 | 1.43470000 | -1.05470000 |
| C | -3.12360000 | 2.67010000 | -1.17210000 |
| N | -1.81050000 | 2.46160000 | -1.17460000 |
| N | -1.59660000 | 1.08880000 | -1.06370000 |
| C | 3.58520000 | -4.09300000 | -1.26020000 |
| C | 4.88410000 | -4.27190000 | -1.78960000 |
| C | 5.38820000 | -5.57770000 | -1.91210000 |
| C | 4.59200000 | -6.66300000 | -1.50270000 |
| C | 3.30910000 | -6.39100000 | -0.98410000 |
| N | 2.80840000 | -5.14170000 | -0.86150000 |
| C | -3.74610000 | 4.00100000 | -1.26700000 |
| C | -5.05460000 | 4.18590000 | -1.77060000 |
| C | -5.55380000 | 5.49420000 | -1.88750000 |
| C | -4.74360000 | 6.57640000 | -1.49830000 |
| C | -3.45210000 | 6.29880000 | -1.00450000 |
| N | -2.95600000 | 5.04730000 | -0.88770000 |
| N | 5.00090000 | -1.45840000 | -0.78090000 |
| C | 5.41320000 | -0.50920000 | 0.03630000 |
| C | 6.82890000 | -0.35870000 | 0.31110000 |
| C | 7.84320000 | -1.05450000 | -0.27140000 |
| S | 9.54750000 | -0.71750000 | 0.20210000 |
| C | 9.09660000 | 0.51600000 | 1.50440000 |
| S | 7.29770000 | 0.91740000 | 1.55670000 |
| C | 10.00320000 | 1.07460000 | 2.33350000 |
| S | 11.80800000 | 0.67780000 | 2.27670000 |
| C | 12.21700000 | 1.69750000 | 3.72780000 |
| C | 11.23630000 | 2.40630000 | 4.31790000 |
| S | 9.55250000 | 2.30780000 | 3.63420000 |
| H | -4.88690000 | -0.28430000 | 0.50880000 |
| H | -7.84580000 | 1.77610000 | -0.96320000 |
| H | -13.37930000 | -1.78860000 | 4.09960000 |
| H | -11.49330000 | -3.21660000 | 5.15890000 |
| H | 1.92740000 | 4.66070000 | -1.42210000 |
| H | 4.30650000 | 3.79820000 | -1.62890000 |
| H | 4.70320000 | 1.34610000 | -1.49510000 |
| H | 0.06530000 | 3.02340000 | -1.19470000 |
| H | -2.08710000 | -4.74400000 | -1.54820000 |
| H | -4.46900000 | -3.88230000 | -1.73010000 |
| H | -4.87140000 | -1.43510000 | -1.53330000 |
| H | -0.22900000 | -3.11020000 | -1.27550000 |
| H | 5.46800000 | -3.41050000 | -2.08540000 |
| H | 6.38150000 | -5.74260000 | -2.32060000 |
| H | 4.94600000 | -7.68670000 | -1.57880000 |
| H | 2.65910000 | -7.19690000 | -0.65270000 |
| H | -5.64980000 | 3.32670000 | -2.04960000 |
| H | -6.55460000 | 5.66320000 | -2.27550000 |
| H | -5.09350000 | 7.60190000 | -1.57050000 |
| H | -2.79110000 | 7.10210000 | -0.68890000 |
| H | 4.72070000 | 0.16410000 | 0.54460000 |
| H | 7.68290000 | -1.80800000 | -1.03220000 |
| H | 13.25350000 | 1.68620000 | 4.04260000 |
| H | 11.36540000 | 3.05070000 | 5.17900000 |
| O | 0.23030000 | 0.13460000 | -2.92370000 |
| C | -0.79180000 | -0.45750000 | -2.89910000 |
| Co | -0.08280000 | -0.04580000 | -1.26280000 |
| O | -1.91090000 | -1.10620000 | -3.50880000 |
| H | -1.78240000 | -1.14070000 | -4.45950000 |

| **[CoL^2^]^2+^-TS3** | | | |
| --- | --- | --- | --- |
| N | 5.02600000 | -1.40940000 | -0.66460000 |
| C | 5.46950000 | -0.49140000 | 0.16500000 |
| C | 6.89730000 | -0.32390000 | 0.36690000 |
| C | 7.88230000 | -0.96950000 | -0.31350000 |
| S | 9.60440000 | -0.62910000 | 0.07130000 |
| C | 9.21790000 | 0.52920000 | 1.46230000 |
| S | 7.41890000 | 0.89340000 | 1.64770000 |
| C | 10.16670000 | 1.06040000 | 2.26140000 |
| S | 11.96930000 | 0.70020000 | 2.07180000 |
| C | 12.45430000 | 1.65420000 | 3.54410000 |
| C | 11.50210000 | 2.31320000 | 4.23050000 |
| S | 9.77970000 | 2.21520000 | 3.65060000 |
| C | -2.31600000 | -3.55430000 | -1.49890000 |
| C | -3.63520000 | -3.05400000 | -1.61040000 |
| C | -3.85710000 | -1.68690000 | -1.43960000 |
| C | -2.76010000 | -0.84090000 | -1.14060000 |
| N | -1.45450000 | -1.33040000 | -1.07770000 |
| C | -1.26200000 | -2.66830000 | -1.25840000 |
| C | -2.77310000 | 0.57380000 | -0.95330000 |
| N | -3.76370000 | 1.56720000 | -0.93580000 |
| C | -3.07650000 | 2.80050000 | -0.96880000 |
| N | -1.76370000 | 2.58170000 | -0.96000000 |
| N | -1.56130000 | 1.19970000 | -0.92530000 |
| C | 2.22820000 | 3.65000000 | -1.31400000 |
| C | 3.54670000 | 3.14830000 | -1.38130000 |
| C | 3.75780000 | 1.77650000 | -1.22120000 |
| C | 2.64990000 | 0.93300000 | -0.98520000 |
| N | 1.34970000 | 1.42280000 | -0.96740000 |
| C | 1.16280000 | 2.76230000 | -1.12980000 |
| C | 2.65430000 | -0.49180000 | -0.81860000 |
| N | 3.64000000 | -1.48000000 | -0.84140000 |
| C | 2.95950000 | -2.70980000 | -0.91330000 |
| N | 1.64640000 | -2.49420000 | -0.87910000 |
| N | 1.44980000 | -1.11570000 | -0.80380000 |
| C | -3.69130000 | 4.13910000 | -1.00270000 |
| C | -4.98230000 | 4.36000000 | -1.53560000 |
| C | -5.47210000 | 5.67570000 | -1.59260000 |
| C | -4.67010000 | 6.72870000 | -1.11600000 |
| C | -3.39650000 | 6.41550000 | -0.59790000 |
| N | -2.90960000 | 5.15620000 | -0.53830000 |
| C | 3.59030000 | -4.03760000 | -1.00550000 |
| C | 4.80000000 | -4.23100000 | -1.71020000 |
| C | 5.31360000 | -5.53360000 | -1.82060000 |
| C | 4.61340000 | -6.59930000 | -1.22460000 |
| C | 3.41570000 | -6.31240000 | -0.53890000 |
| N | 2.90700000 | -5.06420000 | -0.42570000 |
| N | -5.13810000 | 1.49150000 | -0.70220000 |
| C | -5.56420000 | 0.50610000 | 0.06210000 |
| C | -6.98430000 | 0.34390000 | 0.30830000 |
| C | -7.98850000 | 1.06990000 | -0.25410000 |
| S | -9.69930000 | 0.71400000 | 0.17570000 |
| C | -9.27050000 | -0.58630000 | 1.41990000 |
| S | -7.47230000 | -0.99080000 | 1.48240000 |
| C | -10.19110000 | -1.18660000 | 2.20310000 |
| S | -11.99450000 | -0.78660000 | 2.13620000 |
| C | -12.42950000 | -1.88750000 | 3.51890000 |
| C | -11.45850000 | -2.62620000 | 4.08790000 |
| S | -9.76160000 | -2.48550000 | 3.44530000 |
| H | 4.79560000 | 0.15290000 | 0.73440000 |
| H | 7.68370000 | -1.68290000 | -1.10390000 |
| H | 13.50900000 | 1.64740000 | 3.79110000 |
| H | 11.67530000 | 2.91660000 | 5.11340000 |
| H | -2.10010000 | -4.61060000 | -1.61940000 |
| H | -4.46400000 | -3.71750000 | -1.84070000 |
| H | -4.84840000 | -1.27230000 | -1.57490000 |
| H | -0.23520000 | -3.00680000 | -1.20230000 |
| H | 2.01900000 | 4.70850000 | -1.42680000 |
| H | 4.38600000 | 3.81160000 | -1.57010000 |
| H | 4.75220000 | 1.36300000 | -1.32770000 |
| H | 0.13480000 | 3.10150000 | -1.11330000 |
| H | -5.57220000 | 3.52210000 | -1.88270000 |
| H | -6.45910000 | 5.87300000 | -2.00210000 |
| H | -5.01290000 | 7.75870000 | -1.14070000 |
| H | -2.74270000 | 7.19510000 | -0.21510000 |
| H | 5.30750000 | -3.38470000 | -2.15540000 |
| H | 6.23680000 | -5.71260000 | -2.36480000 |
| H | 4.97700000 | -7.62070000 | -1.28660000 |
| H | 2.84220000 | -7.10300000 | -0.06170000 |
| H | -4.88030000 | -0.19260000 | 0.54740000 |
| H | -7.81540000 | 1.86040000 | -0.97350000 |
| H | -13.47210000 | -1.89580000 | 3.81270000 |
| H | -11.60270000 | -3.31810000 | 4.90890000 |
| O | 0.03730000 | 0.01720000 | -3.10090000 |
| C | 1.17800000 | -0.21660000 | -3.69500000 |
| O | 0.99140000 | -0.20290000 | -5.08300000 |
| Co | -0.04770000 | 0.04350000 | -1.13160000 |
| H | 0.03720000 | -0.01990000 | -5.31970000 |
| H | 2.30700000 | -0.42830000 | -3.09270000 |
|  | | | |

**References**

(S1) F. Bentiss, M. Lagrenée, M. Traisnel, B. Mernari, H. Elattari, *J. Heterocyclic Chem.* **1999**, *36*, 149-152.

(S2) J.-F. Geldard, F. Lions, *J. Org. Chem.* **1965**, *30*, 318-319.

(S3) M. Souto, J. Calbo, I. Ratera, E. Ortí, J. Veciana, *Chem. Eur. J.* **2017**, *23*, 11067-11075.

(S4) N.-G. White, H.-L.-C. Feltham, C. Gandolfi, M. Albrechtb, S. Brooker, *Dalton Trans.* **2010**, *39*, 3751-3758.

(S5) M.-J. Frisch, G.-W. Trucks, H.-B. Schlegel, Gaussian 09, revision C.01, Gaussian, Inc.,Wallingford CT, 2010.
